# Harms of massage in persons with cancer or receiving treatment for cancer: A systematic review and meta-analysis

**DOI:** 10.1371/journal.pone.0351878

**Published:** 2026-08-05

**Authors:** Nicolas Kjerulf, Jan Christensen, Mikael Rørth, Anders Larsen, Kira Bloomquist

**Affiliations:** 1 Physiotherapy Branch, Institute for Therapy and Midwifery, Faculty of Health Science, University College Copenhagen, Copenhagen, Denmark; 2 Department of Occupational Therapy and Physiotherapy, Copenhagen University Hospital, Rigshospitalet, Copenhagen, Denmark; 3 Center for Health Research (UCSF), Copenhagen University Hospital, Rigshospitalet, Copenhagen, Denmark; Shahrood University of Technology, IRAN, ISLAMIC REPUBLIC OF

## Abstract

**Introduction:**

Massage is widely used in cancer survivors. However, formal assessment of potential harms is lacking. The aim of this study is to assess harms related to massage.

**Materials and methods:**

This systematic review and meta-analysis included studies that compared massage with control conditions, in people with- and/or receiving treatment for cancer. The primary outcome was adverse events. Ten databases and trials registries were searched up to 22 October 2024. Quality of adverse events reporting was assessed as adherence to the CONSORT statement extension for reporting of harms. Studies reporting on adverse events were assessed for risk of bias using the PEDro scale (randomized controlled/ cross-over trials) and ROBINS-I (quasi-experimental and cohort) and were eligible for meta-analysis (random-effect). The study was registered at PROSPERO (CRD42023352993).

**Results:**

Sixty-three intervention studies were included, of which 53% (n = 34 studies) did not report on adverse events. Twenty-nine studies (n = 2699) reported on adverse events explicity and/or as study discontinuation. Of these, no studies assessed deep massage, with most interventions using light massage. Meta-analyses indicated no evidence of a higher risk of adverse events related to massage compared to usual care/attention control. Three cohort studies (n = 1406) were included, two of which (both with critical risk of bias) found significant associations between massage over tumor site and negative survival outcomes, in patients with osteosarcoma. Overall, the quality of adverse events reporting was poor, and certainty of evidence considered very low.

**Conclusion:**

Results indicate that light to moderate intensity massage in people living with- and/or receiving treatment for cancer can be performed without increased risk of harms. Contraindications for massage should be applied as per the general population. Due to uncertainty as to whether massage over tumor in osteosarcoma constitutes an increased risk of negative survival outcomes, massage directly on tumor is discouraged, though definitive evidence is lacking.

## Introduction

Cancer is a group of diseases characterized by abnormal cell proliferation [1]. Globally, cancer is the leading cause of mortality, accounting for nearly one in six deaths [2]. Encouragingly, though survival rates vary worldwide, advances in treatment have contributed to increased survival in numerous cancer types [2,3]. However, while treatment modalities such as surgery, radiotherapy, chemotherapy and targeted- or immunotherapies are increasingly effective in terms of survival, they are also associated with a range of treatment-specific adverse side- and late-effects that can negatively impact the lives of cancer survivors [4].

There are numerous treatment-specific and general side effects related to cancer treatment such as pain, lymphedema, nausea/vomiting and fatigue [5]. To alleviate these side effects, many patients seek complimentary or alternative medicine (CAM), including massage [6–10]. For example, a recent cross-sectional study including a mixed oncology patient population (n = 171) found that up to 64% used some form of CAM concurrent to receiving treatment, of which 25% was massage [11].

Massage is defined as “a systematic and scientific manipulation of the soft tissues of the body for the purpose of obtaining or maintaining health” [12]. Clinical indications for massage in persons with cancer include symptom relief as part of palliative care, and treatment of- or relief from the side effects of concurrent cancer treatments, with studies indicating beneficial effects on, for example, pain [13,14], fatigue [15] and lymphedema [16]. Indeed, the potential for positive effects related to massage has led some supportive care programs to offer massage as an adjunct to cancer treatment [17–19].

Yet, despite the positive effects of massage in cancer survivors, uncertainty regarding the safety of massage exists [20–24] and clinical massage textbooks cite cancer as an absolute or relative contraindication for massage [12,25,26]. This is due to concerns that 1) massage on cancerous tissue can potentially cause local damage (e.g., fracture to bone with metastasis, or internal hemorrhaging [27], 2) massage can increase the risk of cancer metastasis or recurrence [20,28–30], and 3) potential adverse effects of cancer treatment are contraindications to massage (e.g., thrombocytopenia (increasing the risk of internal bleeding), weakened skin from surgery, and radiation-induced skin injury (increasing the risk of further skin damage and/or -infection) [12,25–27,31]).

To our knowledge, just one systematic review has explored the safety of massage in cancer populations. This systematic review from 2014 (11 original studies, n = 1074) included non-pharmacological interventions for treating pain in patients with advanced cancer, of which five studies specifically assessed massage [32]. The authors were not able to draw any conclusions as to the possible harms of massage due, in part, to the limited number of studies.

To address this knowledge gap, it is the aim of this review to assess harms related to delivery of massage, in people living with- and/or receiving treatment for cancer.

## Materials and methods

This study is reported in accordance with the PRISMA statement (see supplementary materials S1 File) [33] and its extensions for reporting harms and searches [34,35]. A review protocol was registered at PROSPERO (CRD42023352993) on February 7, 2023, and last revision with justification performed on September 27, 2025. Raw data are available as supplementary files S14 and S15.

### Information sources

Preliminary searches and identification of relevant papers were performed to identify search terms and subject headings. An information specialist (AL) developed a search string consisting of four blocks. Search terms included Thesaurus terms and keywords for cancer, massage, contraindication and adverse events. No language or publication date restrictions were imposed. Full search strategies are available (S2 File). Systematic searches for eligible trials were performed up to 22 October 2024, in the following databases and registries; MEDLINE (via PubMed), EMBASE (via Ovid), CINAHL (via EBSCO), Cochrane Central Register of Controlled Trials (CENTRAL) and Web of Science (Sci-EXPANDED/SSCI), PEDro, Scopus and AMED, ClinicalTrials.gov and ISRCTN (United Kingdom). Pearl growing was performed by using backward and forward citation searches of eligible trials with Citationchaser [36,37]. Corresponding authors were contacted via standardized e-mails (two attempts separated by two weeks) if: (1) further information was required to assess eligibility at the full-text level, (2) if conference abstracts, registered trials or protocols with a completed or unsure status were identified, but no peer-reviewed publication could be found.

### Eligibility criteria

Studies were included as per the following eligibility criteria: (1) *Trial characteristics*: peer-reviewed randomized controlled trials (RCTs), quasi-experimental, case-control and cohort studies (containing exposure and reference group); (2) *Population characteristics*: cancer survivors with “active” cancer defined as 1) receiving (neo)adjuvant treatment, or were within six weeks of cancer-related surgery 2) or were living with recurrent, regionally advanced or metastatic cancer, or hematological cancer not in complete remission with or without treatment. Studies including participants only receiving adjuvant endocrine treatment concurrent to massage delivery were excluded; 3) *Intervention characteristics:* massage was defined as Swedish massage (techniques such as effleurage (stroking), petrissage (kneading), friction, tapotement (percussion) or vibration) or other massage techniques that involve applying pressure to soft tissue in excess of light lotioning. Studies were excluded that evaluated acupressure, reflexology, aromatherapy massage, self‐lymphatic drainage or self-massage, or pneumatic compression (or comparable intervention)*; 4) Comparators*: usual care alone or usual care plus other intervention of a character that did not involve manipulation of tissue. Thus, studies with comparators such as acupuncture or another massage type were excluded. In addition, studies were included that compared massage plus a complementary intervention against complementary intervention alone, thus allowing for isolation of massage (e.g., massage plus psychosocial support versus psychosocial support alone).

### Study selection and data collection

The selection of studies was performed independently by two authors (NK, KB) on the basis of *a priori* defined eligibility criteria using Covidence [38]. Search results from the databases were merged and duplicates identified before title and abstract screening. Two authors (NK, KB) independently extracted data using Covidence, into predefined data fields. The quality of adverse events reporting [39] was extracted into predefined data fields in Excel. Any inconsistencies between reviewers were discussed and, if relevant, resolved by a third reviewer (MR).

### Data items

Extracted variables included *study characteristics* (design, aim of study, number of groups, setting, country, and funding source), *participant characteristics* (total number of participants, cancer diagnosis, stage and treatment received, age and sex), *massage intervention characteristics* (region, type, intensity, frequency, time and who delivered massage), and *outcomes of interest* (any adverse events, lost to follow-up, discontinuation or withdrawal due to adverse event, outcomes related to cancer proliferation or metastases). Adverse events were defined as “any unfavorable and unintended sign (including an abnormal laboratory finding), symptom, or disease temporally associated with the use of a medical treatment or procedure that may or may not be considered related to the medical treatment or procedure” [39]. In studies where severity / seriousness was not reported, adverse events were classified according to Common Terminology Criteria for Adverse Events (CTCAE) v5.0 where possible (e.g., discontinuation due to death reported in flow chart was given a grade 5 in severity). Mean and standard deviation (SD), median and range, or count and percentage post intervention/last follow-up were extracted.

### Quality of adverse events reporting

The quality of adverse events reporting was assessed in all intervention studies in adherence to the CONSORT statement extension for reporting of harms [40] using a 16-item scoring system adapted from previous studies [41]. An item was given a score of ‘1’ if reported and a score of ‘0’ if it was unclearly or not reported. Only studies reporting explicitly on adverse events and/or reporting on discontinuations or withdrawals due to adverse events were included for further analysis (risk of bias assessment and synthesis of results).

### Risk of bias in individual studies

In studies reporting on adverse events, the risk of bias was assessed independently by two authors (NK, KB), with inconsistency between reviewers discussed, and if necessary, resolved by a third reviewer (MR or JC). As studies with different study designs were included, assessment of risk of bias was conducted using two different tools. The PEDro scale was used to assess RCTs (including cross-over trials) [42,43]. This 11-item scale, specifically designed for physical therapy interventions (which includes massage), addresses external validity, risk of bias (internal validity) and interpretability. It has been found to detect potential bias with fair to good reliability and is considered a valid measure of methodological quality. The first item does not contribute to the total score because it is related to external validity and is therefore not used to calculate the overall score for each study. Further, in “hands-on” therapies achieving a score of 10 is rarely possible due to the inability to blind the therapist delivering a massage and the person receiving. As such, scores above eight are not likely. As recommended by the Cochrane group [44], the Risk Of Bias In Non-randomized Studies of Interventions (ROBINS-I) tool was used in quasi-experimental and cohort studies [45]. The principal effect of interest was considered the effect of group allocation and target randomized trial and confounders were specified (S3 File).

### Statistical analyses and data synthesis

Continuous variables are presented as mean values ± standard deviations (SD) or median [range]. Categorical and binary variables are presented as frequency (percentages). Studies fulfilling the following criteria were included for meta-analysis: 1) intervention studies (RCT, cross-over and quasi-experimental) reporting on adverse events (either explicitly or as reason for discontinuation/withdrawal) per arm (massage vs. control), 2) had usual care or attention control interventions as control/comparator. Studies with comparators that potentially affected local tissue or circulatory/lymphatic system (e.g., exercise and touch therapies) were excluded. In multi-arm studies with more than one eligible comparator, the comparator arms were pooled. Clinical heterogeneity (e.g., populations, massage characteristics) between studies was assessed prior to conducting meta-analysis. Statistical heterogeneity between studies included in meta-analysis was assessed using the I² statistic. Statistical heterogeneity was interpreted using standard cut-offs; values of 0–40% may indicate low heterogeneity, 30–60% moderate heterogeneity, 50–90% substantial heterogeneity, and 75–100% considerable heterogeneity [46]. All adverse event outcomes were included in the analyses independent of their reported relatedness to the massage intervention. Numbers of adverse events at last follow-up were used for analysis. Effect estimates and 95% confidence intervals (CIs) for adverse events were pooled as risk ratios (RR). A random-effects model (DerSimonian and Laird method) was applied to account for expected heterogeneity across studies. Trials with zero events in both arms were excluded from the primary analyses. As this produces a potential overestimation of adverse event risk, sensitivity analyses were conducted, including studies where no events were observed and imputing 0.5 events [47]. Sensitivity analyses were also performed to assess the influence of quality in adverse event reporting (score ≥5). A separate meta-analysis was performed in studies reporting grade 3–5 adverse events. All meta-analyses were performed using Stata (StataCorp. 2023. *Stata 18*. Statistical software. College Station, TX: StataCorp LLC). Results are displayed in forest plots.

#### Additional analyses.

Sub-group analyses were performed to assess the effect of 1) adverse event reporting type (explicit, discontinued/withdrawal), 2) cancer stage (advanced stage, mixed/other stage, hematology), 3) cancer treatment (chemotherapy, stem-cell, radiotherapy, post-surgery, not specified/mixed, no treatment), and 4) risk of bias (PEDro score classification: < 4 poor, 4–5 fair, 6–8 good, 9–10 excellent), on the relative risk for developing adverse events.

#### Publication bias.

Funnel plots were used to assess publication bias (small study bias) if ≥10 comparisons were made in meta-analyses. Funnel plot asymmetry was assessed by visual inspection and formally tested using the Egger test for continuous outcomes [48].

#### Cohort studies.

Due to inherent differences in study design (harms reported as outcome vs. as an adverse event), data from cohort studies were analyzed separately. As the risk of bias was considered critical in two of the three included cohort studies, no formal meta-analysis was performed [49]. Instead, these studies are presented narratively.

### Certainty of evidence

Two authors (NK, KB) independently assessed the certainty of evidence using the Grading of Recommendations, Assessment, Development, and Evaluations (GRADE) guidelines [50]. Limitations on study design were considered not serious if most or all data came from RCTs, serious if data came from mostly quasi-experimental studies, and very serious if data only came from quasi-experimental or cohort studies. Inconsistency limitations were considered not serious if I^2^ was < 50% with narrow variance of point estimates (RR) across studies and overlap of CIs; serious if I^2^ was between 50–69% and variance of point estimates had some overlap of CIs; and very serious if I^2^ ≥ 70% with wide variance of point estimates across studies and minimal or no overlap of CIs. Limitations in risk of bias were considered not serious if evidence was mostly from studies with a low risk of bias (PEDro ≥ 6, ROBINS-I moderate or less); serious if evidence was mostly from studies with PEDro 4–5 or ROBiNS-I serious rating; and very serious if evidence was mostly from studies with PEDro < 4 or ROBINS-I critical rating. Particular attention was given to item 8 in PEDro as it measure completeness of follow-up, and selected reporting and confounding in ROBINS-I. Limitations on imprecision were considered not serious if data from over ≥385 participants were available per outcome, and if the CI around RR was ≤ 0.5; serious if n < 385 and CI around RR was over 0.5 to 1.0; and very serious if n < 385 and CI around RR > 1.0. Indirectness limitations were considered not serious if most adverse events were reported explicitly or as withdrawal/discontinuations AND was planned/ described in methods; serious if most adverse events were reported explicitly in manus results but not otherwise mentioned; and very serious if most studies only reported adverse events in flow chart, or in text as discontinuation or withdrawal. Limitations in publication bias were considered not serious if no evidence was observed as per funnel plot in analyses with 10 or more studies existed; serious if the funnel plot was unclear; and very serious if funnel plots showed evidence of small study bias. In meta-analyses with <10 studies, publication bias was visually assessed by examining whether studies report different results based on the number of included participants.

## Results

**Fig 1:**
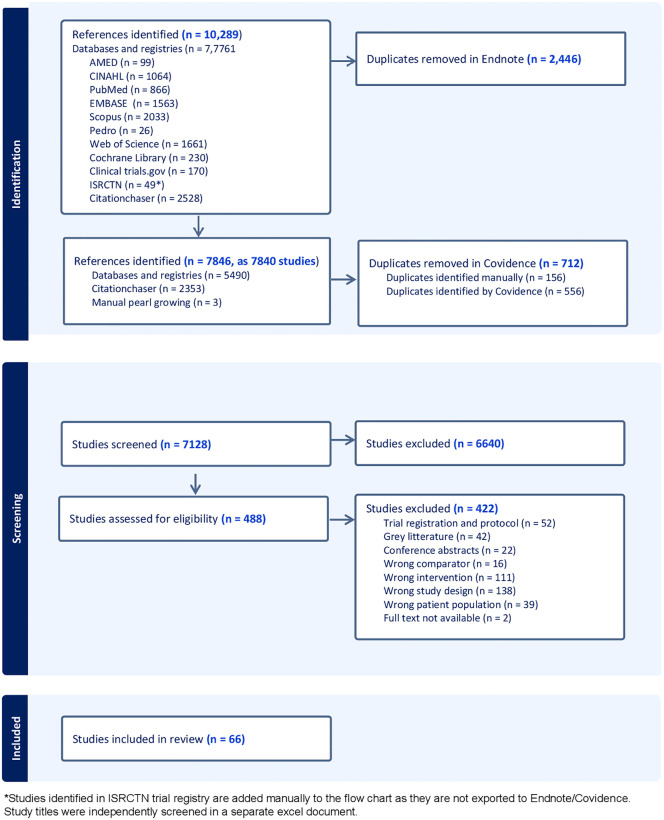
PRISMA flow diagram of included studies.

A total of 7128 articles were screened for eligibility, with 488 articles assessed at the full-text level. Of these, 422 were excluded (reasons for exclusion can be found in S4 File), leaving 66 included studies (Fig 1). Of the 66 studies, 34 intervention studies (RCT, cross-over and quasi-experimental) did not report on adverse events. Twenty-nine intervention studies reported on adverse events (explicitly and/or related to discontinuations and withdrawals) and were included for further analysis [51–82]. Three cohort studies (one of which was two merged articles) assessing the association of massage and survival outcomes (e.g., recurrence and cancer progressivity), were also included [28,29,83,84].

### Study characteristics

Characteristics of all included studies are summarized in Table 1. Overall, studies reporting on adverse events were similar to studies that did not [85–120], except for cancer stage, as more participants with advanced cancer were represented in studies reporting on adverse events vs. those that did not (28% vs. 17%, respectively) (Table 1). Individual study characteristics of intervention studies reporting on adverse events and cohort studies are reported in S5 File.

**Table 1:** Characteristics of included studies.

	All studies (66 studies, n = 6913)	Intervention studies reporting on AEs (29 studies, n = 2699)	Cohort studies (3 studies, n = 1406)
**Study characteristic**	**n (%) / mean±SD**	**n (%) / mean±SD**	**n (%) / mean±SD**
Design (n studies (%))			
Randomized controlled trial	46 (70)	23 (79)	NA
Quasi-experimental	14 (21)	4 (14)	NA
Cross-over	3 (5)	2 (7)	NA
Prospective cohort	1 (2)	NA	1 (33)
Retrospective cohort	2 (3)	NA	2 (67)
Participant characteristics*			
Age adult **	51.8 ± 11.6	52.7 ± 6.5	29.3 ± 19.8^£^
Age pediatric**	9.6 ± 2.3	11.8 ± 2.0	NA
Female ***	5025 (77)	1881 (72)	1221 (87)
Male ***	1538 (23)	717 (28)	185 (13)
Cancer tumor site (n studies (%))^a^			
Lip, oral cavity, or pharynx (2B50-2B5Z)	1 (2)	0	0
Haematopoietic or lymphoid tissues (2A20-2B3Z)	11 (17)	4 (14)	0
Breast (2C30-2C3Z)	19 (29)	6 (21)	1 (33)
Osteosarcoma (XH1XF3)	2 (3)	0	2 (67)
Digestive system (2D80-2D8Z)	2 (3)	2 (7)	0
Mixed tumor sites	28 (42)	15 (52)	0
Not reported	3 (5)	2 (7)	0
Cancer stage (n studies (%))			
Early (stage 1 & 2)	2 (3)	0	0
Advanced (stage 3 & 4)	11 (17)	8 (28)	0
Early and advanced	21 (32)	8 (28)	1 (33)
Not reported	32 (48)	13 (45)	2 (67)
Evidence of disease status (n studies (%))			
No evidence of disease	12 (18)	3 (10)	1 (33)
Evidence of disease	27 (41)	13 (45)	2 (67)
Mixed	7 (11)	4 (14)	0
Unclear	20 (30)	9 (31)	0
Treatment during intervention (n studies (%))^b^			
Chemotherapy	23 (35)	12 (41)	0
Radiotherapy	5 (8)	2 (7)	0
Post-surgery (within 6 weeks)	6 (9)	3 (10)	0
During surgery	1 (2)	0	0
Chemoradiotherapy	1 (2)	1 (3)	0
Stem cell transplantation	1 (2)	1 (3)	0
Yes, mixed treatments	14 (21)	4 (14)	1 (33)
No anti-cancer treatment	3 (5)	1 (3)	2 (67)
Not reported	12 (18)	5 (17)	0

Means and associated standard deviations (SD) are calculated based on means reported in studies.

The sum of percentages does not necessarily add up to 100% due to rounding. Abbreviations: AE; adverse events.

*Based on reported baseline characteristics from respective studies.

**Age adults based on 52 and 28 studies for all studies and intervention studies, respectively. Age pediatric studies based on 8 and 1 studies for all studies and intervention studies, respectively. ***Sex based on 62 studies for all studies, and 28 studies for intervention studies reporting on AEs. ^£^Two populations are mixed children/adults. ^a^Code in parentheses refers to cancer sites as classified by the International Classification of Diseases, 11th Revision.^b^ “Yes” if all participants were receiving given treatment during the intervention period.

Of the 32 studies reporting on adverse events [28, 51–81, 81–84,121], the majority were conducted in three regions: North America (38%, n = 12) [51,53–56,59,67,68,70,72,73,82], the Middle East (31%, n = 10) [52,57,61–66,71,79], and East Asia (22%, n = 7) [29,58,60,77,78,80,83] (S5 File). Almost all (97%, n = 28) were performed in adults. A little over half of the studies included mixed tumor site populations [51,53–55,58–61,66–68,70,73,78,80], with breast cancer representing the largest specific cancer type. Most studies (79%) included participants receiving treatment [51,52,54–58,61–69,71–77,79,80,82,120], of which chemotherapy was the most frequent (Table 1).

Massage characteristics were similar between studies reporting and not reporting on adverse events, with Swedish massage techniques as the most common type (Table 2). Only three studies described massage intensity as “deep” [85,97,120], but none of these studies reported on adverse events. In studies reporting on adverse events over half of studies used light/gentle intensity [51–53,56,57,59,63–65,67,69,72–78,80,82], with a wide range of massage dosage (frequency and duration) (Table 2 and S5 File).

**Table 2:** Massage characteristics of included studies.

	All studies (66 studies, n = 6916)	Intervention studies reporting on AE (29 studies, n = 2699)	Cohort studies (3 studies, n = 1406)
**Massage characteristic**	**n (%) / Median [range]**	**n (%) / Median [range]**	**n (%) / Median [range]**
Massage type (n studies (%))			
Manual lymph drainage	5 (8)	1 (3)	1 (33)
Slow-stroke back	6 (9)	3 (10)	0
Swedish techniques*	35 (53)	16 (55)	0
Abdominal	4 (6)	3 (10)	0
Other (fx Thai, chair)	16 (24)	6 (21)	2 (67)
Massage region			
Whole body or multiple regions	17 (26)	10 (33)	2 (67)
Back, neck and/or upper extremity/breast	23 (35)	7 (23)	0
Distal extremities (Hand/lower arm/foot/lower leg)	12 (18)	5 (17)	0
Abdomen	5 (8)	2 (7)	0
Lower extremity	1 (2)	0	1 (33)
Patient’s preference/Not reported	7 (11)	5 (20)	0
Over cancerous tissue (e.g., tumor site, bone met)^a^	5 (8)	3 (10)	2 (67)
Massage intensity (n studies (%))			
Gentle/light	34 (52)	17 (59)	1 (33)
Gentle/light and moderate	7 (11)	5 (17)	1 (33)
Included deep	3 (5)	0	0
Not clear or not reported	22 (33)	7 (24)	1 (33)
Massage Dosage			
Single massage only (duration range: 5–75 min.)	6 (9)	1 (3)	NR
Frequency (sessions/week)**	2.7 [0.33 to 10]	3.0 [0.33 to 9]	NR
Duration of intervention (weeks)**	2.5 [1 –26]	3,0 [1 –12]	NR
Total number of treatments**	5 [1 –51]	6 [1 –18]	NR
Total treatment time (minutes)**	120 [5–1200]	138 [20–105 0]	NR
Massage delivered by			
Massage therapist	17 (26)	11 (38)	0
Health professional with massage training	28 (42)	8 (28)	1 (33)
Spouse/caregiver (with instruction)	6 (9)	4 (14)	0
Other (fx. Researcher, surgeon)	7 (11)	4 (14)	2 (67)
Mechanical massage chair	1 (2)	1 (3)	0
Not reported	7 (11)	1 (3)	0

Medians and associated ranges are calculated based on medians reported in studies. The sum of percentages does not necessarily add up to 100% due to rounding. Abbreviations: AE, adverse events.

* Including effleurage (stroking), petrissage (kneading), friction, tapotement (percussion) or vibration. ^a^ Counted twice as both reported as region and cancerous area.

**Frequency based on 63 and 29 studies for all studies and intervention studies, respectively. Duration based on 62 and 29 studies for all studies and intervention studies, respectively. Total number of treatments based on 62 and 29 studies for all studies and intervention studies, respectively. Total treatment time based on 60 and 29 studies for all studies and intervention studies, respectively.

### Quality of adverse events reporting

Overall, the quality of adverse event reporting was poor, with over half of the intervention studies (n = 34) not reporting on any aspect of adverse events (S6 File). Of the 29 intervention studies that did report on adverse events, the median score was 1.5 items (range: 1–10). Reporting on seriousness and severity was largely absent: only one study provided explicit information on severity [80], and no studies reported on seriousness. Five studies reported on adverse events per type [54,59,70,78,80] (Table 3 and S6 File).

**Table 3:** Quality of adverse events reporting in intervention studies providing information on adverse events (29 studies).

Items	Item reported, n (%)
Introduction	
1. In the title or abstract, trial states safety or adverse events were assessed.	3 (10)
2. In the introduction, trial states that safety or adverse events were assessed.	2 (7)
Methods	
3a. Trial lists and defines all adverse events that were assessed.	4 (13)
3b. Trial specifies instruments that were used to assess adverse events.	1 (3)
4a. Trial describes how adverse events were collected.	7 (23)
4b. Trial describes when adverse events were collected.	8 (27)
4c. Trial describes attribution method.	1 (3)
5. Trial describes how adverse events were analyzed.	2 (7)
Results	
6a. Trial reports number patients discontinuing or withdrawing due to adverse events per arm	26 (90)
6b. Trial describes adverse events leading to patient discontinuation or withdrawal. *	14 (48)
7. Trial provides denominators used for each analysis of adverse events.	2 (7)
8a. Trial reports adverse events per severity	1 (3)
8.b Trial reports adverse events per type **	5 (17)
8.c Trial reports adverse events per seriousness	0 (0)
8.d Trial describes handling of recurrent events	0 (0)
Discussion	
10. Trials provides a balanced discussion of benefits and harms	4 (14)

*n = 9, not applicable as there were no discontinuations due to adverse events. ** n = 9, not applicable as no adverse events were reported

### Risk of bias in intervention studies

All but one study [51] met external validity conditions (item 1) by specifying the source of participants and eligibility criteria (S7 File). Less than half of studies met criterion for concealed allocation, assessor blinding, and attention-to-treat analysis. In contrast, all studies met criterion for random allocation, reporting between-group differences, point measures, and variability. For quasi-experimental studies, the overall risk of bias was judged as moderate in one study [78], and serious for the remaining three [74,77,79], with confounding and measurement outcome domains considered sources of high bias (S8 and S9 File).

### Adverse events

Fourteen studies reported explicitly on adverse events. Of these, nine found no adverse events in either group [55,56,58,66,67,69,72,73,79]. Of the five studies that did report adverse events, four presented events per intervention group [54,59,70,78] (Table 4 and S5 File). The fifth study, a cross-over trial comparing mechanical chair massage with usual care [80], reported 80 adverse events (15 of those grade 3 or 4), but did not differentiate between groups and attributed all events to chemotherapy. Sixteen studies reported adverse events as reason for trial withdrawal or discontinuation (e.g., flow diagram).

**Table 4:** Adverse events in intervention studies.

Adverse event explicitly reported	Studies reporting (n)	Massage (n)	Comparator (n)	Severity/seriousness (n)	Last follow-up
Respiratory infection [59]	1	1	0	Serious	1,5 wks PI
GI bleed [59]	1	1	0	Serious	1,5 wks PI
Heart failure [59]	1	0	1	Serious	1,5 wks PI
Seizure [59]	1	0	1	Serious	1,5 wks PI
Fracture [59]	1	0	1	Serious	1,5 wks PI
Pain control [59]	1	0	3	Serious	1,5 wks PI
Death [59,70]	2	26 0	33 1	Serious Serious	1,5 wks PI 3 wks PI
Presyncope [54]	1	2	0	NR	“Immediately” after LI
Pain [70]	1	1	0	NR	“After second massage”
Acute renal failure [70]	1	1	0	NR	3 wks PI
Dyspnea & pain [70]	1	1	0	NR	3 wks PI
Shortness of breath [70]	1	1	0	NR	“After second massage”
Temporary skin erythema [78]	1	1	0	NR	Same day as LI
**Adverse event reported as discontinuation or withdrawal**	**Studies reporting(n)**	**Massage(n)**	**Comparator(n)**	**Severity /seriousness (n)**	**Last follow-up**
Medical complications [51]	1	0	1	NR	Same day as LI
Worsening of illness or death [53]	1	6	15	NR	2 months PI
Hospitalization [55]	1	1	0	NR	4 wks PI
Disease progression [58]	1	1	0	NR	1 day PI
“Became too ill” [82]	1	0	2	NR	Same day as LI
Death [50,62,68]	3	0 4 19	1 15* 17	NR NR NR	4 months PI 2 wks PI 30 months PI
Physical distress [58]	1	1	2	NR	1 day PI
Feeling stressed [58]	1	0	1	NR	1 day PI
Increased use of laxatives [60]	1	2	3**	NR	Same day as LI
Shortness of breath [60]	1	1	0	NR	Same day as LI
Fatigue [60]	1	1	0	NR	Same day as LI
Fever & leukocytopenia [62,71,77]	3	2 0 0	0 1 3	NR NR NR	24 hrs PI 3 wks PI Same day as LI
Reduced thrombocytes [71]	1	0	1***	NR	3 wks PI
Deterioration in condition [63,64]	2	1 0	0 1	NR NR	2 wks PI 2 wks PI
Hospitalization and death [65]	1	2	0	NR	Same day as LI
Hospitalization [63,65]	2	0 0	1 2	NR NR	4 wks PI Same day as LI
Lack of cooperation or death [65]	1	0	5****	NR	Same day as LI
Pain [67]	1	0	1	NR	1 day PI
Severe diarrhea [71]	1	1	0	NR	3 wks PI
Radiation dermatitis [71]	1	1	1	NR	3 wks PI
Dizziness [77]	1	0	1	NR	Same day as LI

Abbreviations: PI, post intervention. LI, last intervention,

*Attention control (n = 9), therapeutic touch (n = 6); **Usual care (n = 1), aroma massage (n = 2); ***Reflexology group;

**** “Hospitalization” in standard care, “lack of cooperation or death” in aroma inhalation.

**Fig 2:**
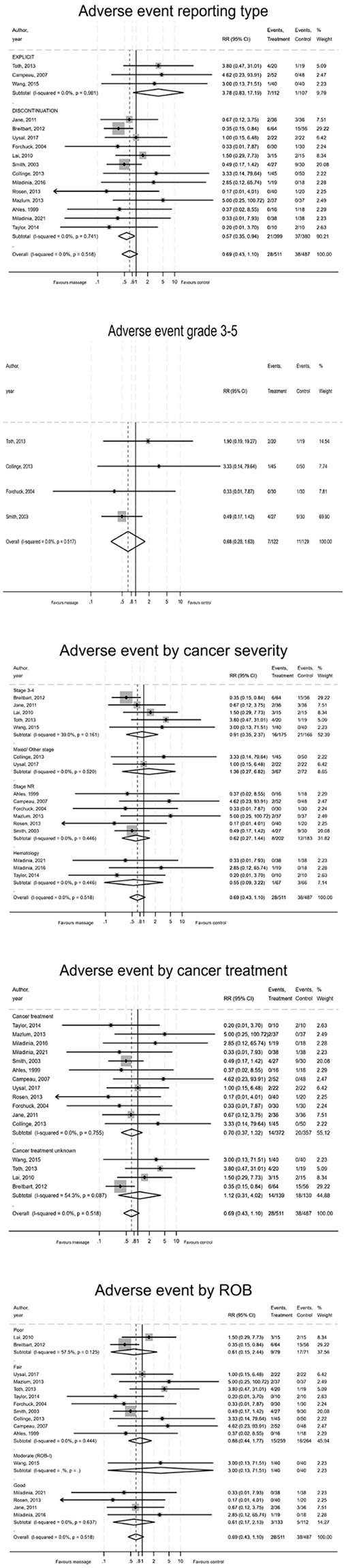
Meta-analyses of massage trials reporting on adverse events. A relative risk (RR) over 1 suggests increased risk of adverse events for those in massage intervention groups. Abbreviations: ROB, risk of bias.

Overall, meta-analysis indicated no statistical evidence of a higher risk of adverse events related to massage compared to control conditions (RR 0.69 (95% CI 0.43 to 1.10), I2 = 0.0%, very low certainty of evidence (S10 File)). This was found irrespective of adverse event reporting type, severity (grade 3–5) of adverse event, cancer stage, cancer treatment, and risk of bias (Fig. 2, Table 5). The interpretation of the findings remained consistent when sensitivity analysis, including studies with no adverse events and quality of adverse event reporting was considered (S11 and S12 File). Certainty of evidence for all outcomes was rated as very low (S10 File). No evidence of publication bias was observed (S13 File).

**Table 5:** Summary table of key outcomes (overall meta-analysis and sub-group analyses) in interventions studies reporting on adverse events.

Adverse event outcomes	K	N	Relative risk [95% CI]	Heterogeneity (I^2^)	Certainty of evidence
Overall	16	998	0.69 [0.43, 1.10]	0%	Very low
Reporting type *					
Explicit	3	219	3.78 [0.83, 17.19]	0%	Very low
Discontinuation	13	779	0.57 [0.35, 0.94	0%	Very low
Serious (grade 3–5) [39]	4	251	0.68 [0.28, 1.63]	0%	Very low
Cancer severity					
Cancer stage 3–4	5	341	0.91 [0.35, 2.37]	39%	Very low
Mixed stages	2	139	1.36 [0.27, 6.82]	0%	Very low
Stage not reported/unknown	6	385	0.62 [0.27, 1.44]	0%	Very low
Hematology	3	133	0.55 [0.09, 3.22]	0%	Very low
Cancer treatment					
Receiving cancer treatment	12	729	0.70 [0.37, 1.32]	0%	Very low
Cancer treatment unknown	4	269	1.12 [0.31, 4.02]	54.3%	Very low
Risk of bias					
Poor (PEDro [42] score <4)	2	150	0.61 [0.15, 2.44]	57.5%	Very low
Fair (PEDro score 4–5)	9	523	0.88 [0.44, 1.77]	0%	Very low
Moderate (ROB-I)	1	80	3.0 [0.13, 71.51]	NA	One study
Good (PEDro score 6–8)	4	245	0.61 [0.17, 2.13]	0%	Very low

*Reporting of adverse event explicitly in the manuscript or as a reason for discontinuation or withdrawal (fx in flow chart)

### Cohort studies

Three cohort studies were included. One retrospective study [83] investigated the relative risk of cancer recurrence in women who received massage as manual lymph drainage (MLD) for breast cancer-related lymphedema (n = 1106). No increased risk of recurrence was observed between the cohort of women receiving MLD vs. those that did not (hazard ratio 0.71 (95% CI 0.39–1.29) during the mean follow-up period of 3.3 ± 1.6 years. Risk of Bias was rated as “serious”, based primarily on the potential for confounding (S8 and S9 File).

The other two cohort studies examined the association of massage delivered before diagnosis of osteosarcoma and progressivity of disease /survival outcomes [28,29,84]. Massage exposure data were collected retrospectively at the time of diagnosis. The time frame for massage received was only specified for 134/200 participants in one study (2.8 wks) [29,84] and not specified in the other [28]. Neither massage intensity nor type of massage were described. Among survival outcomes, Wang et al. (n = 200) found a 5-year survival rate of 53% in patients that self-reported receival of massage over the area later diagnosed with osteosarcoma vs. 98% in patients that had not received massage (p < 0.001), with a recurrence rate of 29% vs. 6%, respectively (p < 0.001) [29,84]. In contrast, Karda et al. (n = 84) found that massage of the area later diagnosed with osteosarcoma did not result in a statistically significant increased risk of lung metastases during the one-year follow-up (RR: 1.538 (95% CI (0.546–4.338), but did find a shorter time to recurrence in the massage vs. no-massage group (4 vs. 12 months respectively, p < 0.0001). At time of diagnosis, both studies reported baseline differences between groups related to prognosis including increased levels of serum lactate dehydrogenase [28,29,84], alkaline phosphatase [28], and a higher rate of lung metastasis (31% vs. 3%, p = 0.003 [29,84] and 45% vs. 31%, p = 0.225) [28] in the massage vs. no-massage groups, respectively. The risk of bias in both studies was rated as “critical”, primarily due to risk of confounding (S8 and S9 File).

## Discussion

Overall, the results of this study indicate that massage can be delivered to persons with- and/or receiving treatment for cancer without increased risk of harms. However, several factors should be taken into consideration when interpreting these results.

First, the quality of adverse event reporting was lacking or poor, with half of the included intervention studies not reporting on any aspect of adverse events. In the twenty-nine studies that did report on adverse events, the median score per study was 1.5 items (out of 16), just one study described how the relatedness of adverse events was assessed (attribution method) [72], and one study explicitly reported on severity [80]. This lack of transparent reporting limits the credibility of the findings.

Second, while the intervention studies reporting on adverse events were conducted in both Western and Eastern countries, by far the most common massage types were Swedish massage techniques. Some countries/regions have massage traditions of a different nature, e.g., Tui Na, Shiatsu, or Thai massage. These massage types may be applied without oil and without asking the patient to disrobe, the intensity may be deep, and they may include other treatment modalities such as stretching and joint mobilization. Thus, the generalization of our findings to these types of massage may be limited.

Further, in line with concerns that potential adverse effects of cancer treatment are contraindications to massage, characteristics of the population of included studies should be noted. Specifically, related to exclusion criteria, more than half of the studies reporting on adverse events excluded participants if they presented with adverse events related to cancer or cancer treatment such as low platelet count, fever, or wounds/ lesions in the target massage area (S5 File). This is important to consider in the generalizability of massage to the larger population of cancer survivors. In addition, almost all participants were adults with a mean age of 53 limiting generalizability to pediatric populations and older frail persons.

Relatedly, aspects of the massage protocols described in the intervention studies should also be considered, especially the intensity of massage. Massage intensity was predominantly light or light to moderate, with only three studies describing massage intensity as “deep” [85,97,120]. However, as none of these studies reported on adverse events, we cannot draw conclusions about the harms associated with deeper intensities. Notably, description of intensity /depth of massage was based on words such as gentle/light, with objective descriptions of intensity/depth lacking in almost all studies. Thus, a potential misclassification of intensity exists. In general, detail regarding massage techniques was limited and should be clearly described in future work, including objective descriptions of massage intensity.

Light intensity massage is used in “oncology massage” as it is practiced and promoted by many schools [12,25,26]. Nonetheless, cancer survivors may seek massage where deep intensities are warranted for better effect (e.g., scar tissue or chronic soft tissue injury) [122–124]. In these cases, it is prudent to ensure that no contraindications to massage (e.g., thrombocytopenia or wounds/lesions) exist before engaging in deep tissue massage (as it seems reasonable to assume that any adverse effect induced by massage would be exacerbated by deeper intensity). Notably, no adverse events related to massage were reported in a RCT (n = 269) exploring the effects of a multimodal intervention, including exercise and massage, in cancer patients receiving chemotherapy [19]. Massage was relaxing, facilitative, or therapeutic, including application on scar tissue with no intensity limitation. Importantly, daily screening including temperature, signs of bruising or bleeding were carried out. This provided the physical therapists delivering the intervention the ability to individualize the intensity of massage based on individual needs of the participant and eventual contraindications.

Finally, related to concerns that massage over cancerous tissue and risk of local damage, three trials did apply light/gentle massage over areas with known cancers. Two RCT’s delivered modified (gentle/light) massage over areas with bone metastases (n = 452) [58,59], and one quasi-experimental study (n = 80) delivered abdominal massage (pressure < 0.5 cm) over end-stage gastro-intestinal cancers [78]. No adverse events related to local injury were reported. No massage interventions were applied over known, superficial cancerous tissue. Either the massage was performed on areas of the body away from the tumor or metastasis site (e.g., foot massage given to people with breast cancer), or modifications were made to avoid massage over the affected area. As such, though we cannot present evidence to support or refute concerns that massage over superficial cancerous tissue may increase the risk of local damage, in cases with, for example, open wounds, radiation dermatitis or bone metastases, avoidance of massage or modifications in intensity are sensible precautions as per contraindications for massage.

Two cohort studies did assess massage delivered over the area later diagnosed with osteosarcoma (implying that massage was delivered on cancerous tissue). Both studies found evidence to suggest an association between massage over cancerous tissue and negative survival outcomes. However, these studies have serious methodological concerns. The risk of bias for both studies was judged as “critical”, primarily due to risk of confounding, including pre-diagnosis pain. While pain is not considered an independent prognostic factor in osteosarcoma, pain is correlated with other prognostic variables (e.g., tumor size/ burden) [125,126]. Relatedly, at time of diagnosis, both studies reported between group differences of relevance for survival outcomes. This includes increased levels of serum biomarkers and higher rates of lung metastases in the massage vs. no-massage groups, respectively [28,29,84]. As temporality of massage exposure and these baseline findings cannot be established, considerable uncertainty exists as to whether survival outcomes indeed are a result of massage received or are a result of baseline differences in important prognostic drivers of osteosarcoma survival. Also, the time frame for when pre-diagnosis massage was received was only specified in a proportion of the total population receiving massage in the two studies, leaving uncertainty as to whether massage was indeed carried out on cancerous tissue in approximately half of participants. Furthermore, due to the retrospective collection of massage data, recall bias related to the area of massage received is a potential issue, and variables such as massage intensity, duration, exact application etc. are unknown, lending uncertainty as to the massage received. Collectively, multiple potential significant biases exist, leaving considerable uncertainty as to whether the observed negative outcomes in osteosarcoma are related to massage. Finally, osteosarcoma is a rare cancer type accounting for less than 0.2% of all cancer cases globally [3] and it is questionable whether results can be extrapolated to other cancer types. Nevertheless, results from these studies do serve as reminders of the importance of basic massage education, interviewing skills related to pain, and referral to health care professionals when appropriate.

Animal studies have suggested that massage directly on different types of tumor tissue in mice can result in cancer metastasis [29,127,128]. However, the extent to which these results can be generalized to human conditions is debatable [129]. In humans, while direct pressure or manipulation of ductal carcinomas can result in epithelial cell shedding to sentinel lymph nodes [130], many other factors play a part in the path from initial shedding of cancer cells, to actual metastasis [30,131]. Metastasis is a complex process initiated by physical dissemination of cancer cells from the primary tumor to distant tissues, and the adaptation of these cells to foreign tissue microenvironment. Formation of macroscopic metastases with clinical significance is not directly coupled with physical dissemination, as evidenced by the presence of a myriad of micro metastases that have successfully disseminated but never progress to macroscopic metastatic tumors. The process of colonization is likely to encompass many cell-biological programs that are considerably more complex and diverse than the preceding steps of metastatic dissemination [132,133]. Currently, evidence is lacking to explain how massage could lead to metastasis in these established pathways.

### Strengths and limitations

Methodological strengths include a pre-registered protocol, and transparent disclosure of protocol deviations. Also, the comprehensive search strategy including forward/backward citation searches, and the independent assessment and data extraction of studies by two authors add credibility to the findings.

Methodological limitations which may limit the generalizability of this study are the exclusion of people receiving only endocrine treatment, and the exclusion of interventions consisting of acupressure, reflexology, self-massage techniques, and pneumatic compression. The poor quality of adverse event reporting and very low certainty of evidence ratings are not methodological limitations but should be considered in the interpretation of the results of this study.

## Conclusion

In summary, the results of this work indicate that light to moderate intensity massage can be performed without increased risk of harms in people living with- and/or receiving treatment for cancer. Massage practitioners should familiarize themselves with the contraindications for massage, many of which may be more frequent in oncology patients. This study is unable to assess the safety of deep massage in oncology. As such, special care should be taken (e.g., screening for contraindications), especially before engaging in deeper tissue massage. Overall, the reporting of adverse events was poor. Future studies should adhere to harms reporting guidelines to better understand potential adverse effects of massage in cancer populations. Due to uncertainty as to whether massage over tumor in osteosarcoma constitutes an increased risk of negative survival outcomes, massage directly on tumor is discouraged, though definitive evidence is lacking.

### Declaration of generative AI

Non-English studies were translated using Microsoft Copilot, Chat GPT and DeepL to ensure the best translation. The content and analysis presented were independently generated and did not involve the use of any AI model.

## Supporting information

S1 File PRISMA checklist. (DOCX)

S2 File Search strategies. (PDF)

S3 File Specification of target trial. (DOCX)

S4 File Reasons for exclusion at full text. (XLSX)

S5 File Characteristics of studies reporting on AEs, and cohort studies. (DOCX)

S6 File Quality of AE report individual. (XLSX)

S7 File Risk of bias included RCT studies (PEDro). (XLSX)

S8 File Risk of bias ROBINS-I. (XLSX)

S9 File ROBINS-I per individual study. (DOCX)

S10 File Certainty of evidence. (XLSX)

S11 File Sensitivity analyses including studies with zero adverse events. (PDF)

S12 File Sensitivity analyses of adverse event reporting score ≥5. (PDF)

S13 File Funnel plots. (PDF)

S14 File Raw data for meta-analysis. (XLSX)

S15 File Raw data for descriptive analyses. (XLSX)
