# Supplementary material for: Harms of massage in persons with cancer or receiving treatment for cancer: A systematic review and meta-analysis
Source: PLoS One. 2026 Aug 5;21(8):e0351878. doi: 10.1371/journal.pone.0351878 (PMC13440881; doi:10.1371/journal.pone.0351878)
Supplement: Supplementary file 1 [file pone.0351878.s002.pdf]

**AMED - The Allied and Complementary Medicine Database (EBSCOhost),** 01-11-2022, **Update:** 02-10-2024

**S1**
TX (cancer*) OR TX (carcinom*) OR TX (leucaemia*) OR TX (leucemia*) OR TX (leukaemia*) OR TX (leukemia*) OR TX (malignan*) OR TX (metasta*) OR TX (myeloma*) OR TX (neoplas*) OR TX (oncology) OR TX (tumor*) OR TX (tumour*)

**S2**
TX (acupressure) OR TX (cupping) OR TX (lymph W1 drainage W1 massage) OR TX (manual W1 lymph W1 drainage) OR TX (manual W1 lymphatic W1 drainage) OR TX (massage) OR TX (musculoskeletal W1 manipulation*) OR TX (osteopathic W1 manipulat*) OR TX (osteopathic W1 treatment) OR TX (osteopathic W1 visceral W1 manipulation*) OR TX (osteopathic W1 visceral W1 treatment) OR TX (reflexological) OR TX (reflexology) OR TX (rolfing) OR TX (shiatsu) OR TX (soft W1 tissue W1 therapy) OR TX (tui W1 na) OR TX (tuina) OR TX (zone W1 therapy)

**S3**
TX (adverse W1 effect*) OR TX (adverse W1 health W1 care W1 event*) OR TX (adverse W1 side W1 effect*) OR TX (after W1 effect*) OR TX (adverse W1 event*) OR TX (complication*) OR TX (contraindication*) OR TX (metastasis) OR TX (neoplasm* W1 recurrence*) OR TX (precaution*) OR TX (safety) OR TX (side W1 effect*) OR TX (tumor N2 recurrence*) OR TX (tumour N2 recurrence*)

**S4**
S1 AND S2 AND S3 = **97**

**Update:**
**S4**
S1 AND S2 AND S3 = 105

**S5**
S1 AND S2 AND S3, Limiters - Publication Date: 20220901-20241231 = **2**

**CINAHL Plus with Full Text (EBSCOhost),** 28-10-2022, **Update:** 23-09-2024

**S1**
MH "Neoplasms+" OR MH "Oncology" OR cancer* OR carcinom* OR leucaemia* OR leucemia* OR leukaemia* OR leukemia* OR malignan* OR metasta* OR myeloma* OR neoplas* OR oncology OR tumor* OR tumour*

**S2**
MH "Acupressure+" OR MH "Cupping Therapy" OR MH "Manual Lymphatic Drainage" OR MH "Manipulation, Orthopedic" OR MH "Massage+" OR MH "Reflexology" OR MH "Rolfing" OR acupressure OR cupping OR (lymph W1 drainage W1 massage) OR (manual W1 lymph W1 drainage) OR (manual W1 lymphatic W1 drainage) OR massage OR (musculoskeletal W1 manipulation*) OR (osteopathic W1 manipulat*) OR (osteopathic W1 treatment) OR (osteopathic W1 visceral W1 manipulation) OR (osteopathic W1 visceral W1 treatment) OR reflexological OR reflexology OR rolfing OR shiatsu OR (soft W1 tissue W1 therapy) OR (tui W1 na) OR tuina OR (zone W1 therapy)

**S3**
MH "Adverse Health Care Event" OR MH "Neoplasm Metastasis+" OR MH "Neoplasm Recurrence, Local" OR MH "Safety+" OR MH "Treatment Complications, Delayed" OR (adverse W1 effect*) OR (adverse W1 health W1 care W1 event*) OR (adverse W1 side W1 effect*) OR (after W1 effect*) OR (adverse W1 event*) OR complication* OR contraindication* OR metastasis OR (neoplasm* W1 recurrence*) OR precaution* OR safe* OR (side W1 effect*) OR (tumor N2 recurrence*) OR (tumour N2 recurrence*)

**S4**
S1 AND S2 AND S3 = **948**

**Update:**
**S4**
S1 AND S2 AND S3 = 966

**S5**
ZD (202210* OR 202211* OR 202212* OR 2023* OR 2024* OR "in process")

**S6**
S4 AND S5 = **116**

**ClinicalTrials.gov (NLM)**, 03-11-2022, **Update 24-09-2024**

**Condition or disease**
cancer* OR carcinom* OR leucaemia* OR leucemia* OR leukaemia* OR leukemia* OR malignan* OR metasta* OR myeloma* OR neoplas* OR oncology OR tumor* OR tumour*

**Other terms**
massage OR "musculoskeletal manipulation*" OR "soft tissue therapy"

**130 Studies found for:** massage OR "musculoskeletal manipulation*" OR "soft tissue therapy" | cancer* OR carcinom* OR leucaemia* OR leucemia* OR leukaemia* OR leukemia* OR malignan* OR metasta* OR myeloma* OR neoplas* OR oncology OR tumor* OR tumour*

**Update:**

**Condition or disease**
cancer* OR carcinom* OR leucaemia* OR leucemia* OR leukaemia* OR leukemia* OR malignan* OR metasta* OR myeloma* OR neoplas* OR oncology OR tumor* OR tumour*

**Other terms**
massage OR "musculoskeletal manipulation*" OR "soft tissue therapy"

**Search Results**
**40 studies**
Showing results for: cancer* OR carcinom* OR leucaemia* OR leucemia* OR leukaemia* OR leukemia* OR malignan* OR metasta* OR myeloma* OR neoplas* OR oncology OR tumor* OR tumour* | Other terms: massage OR "musculoskeletal manipulation*" OR "soft tissue therapy" | First posted from 10/01/2022 to 09/30/2024

**Cochrane Library (Wiley)**, 28-10-2022, **Update 24-09-2024**

**#1**
[mh "Neoplasms"] OR (cancer*):ab,ti,kw OR (carcinom*):ab,ti,kw OR (leucaemia*):ab,ti,kw OR (leucemia*):ab,ti,kw OR (leukaemia*):ab,ti,kw OR (leukemia*):ab,ti,kw OR (malignan*):ab,ti,kw OR (metasta*):ab,ti,kw OR (myeloma*):ab,ti,kw OR (neoplas*):ab,ti,kw OR (oncology):ab,ti,kw OR (tumor*):ab,ti,kw OR (tumour*):ab,ti,kw

**#2**
[mh "Acupressure"] OR [mh "Cupping Therapy"] OR [mh ^"Musculoskeletal Manipulations"] OR [mh "Manual Lymphatic Drainage"] OR [mh "Manipulation, Orthopedic"] OR [mh "Massage"] OR [mh "Therapy, Soft Tissue"] OR (acupressure):ab,ti,kw OR (cupping):ab,ti,kw OR (lymph NEXT drainage NEXT massage):ab,ti,kw OR (manual NEXT lymph NEXT drainage):ab,ti,kw OR (manual NEXT lymphatic NEXT drainage):ab,ti,kw OR (massage):ab,ti,kw OR (musculoskeletal NEXT manipulation*):ab,ti,kw OR (osteopathic NEXT manipulat*):ab,ti,kw OR (osteopathic NEXT treatment):ab,ti,kw OR (osteopathic NEXT visceral NEXT manipulation):ab,ti,kw OR (osteopathic NEXT visceral NEXT treatment):ab,ti,kw OR (reflexological):ab,ti,kw OR (reflexology):ab,ti,kw OR (rolfing):ab,ti,kw OR (shiatsu):ab,ti,kw OR (soft NEXT tissue NEXT therapy):ab,ti,kw OR (tui NEXT na):ab,ti,kw OR (tuina):ab,ti,kw OR (zone NEXT therapy):ab,ti,kw

**#3**
[mh "Contraindications"] OR [mh "Neoplasm Metastasis"] OR [mh "Neoplasm Recurrence, Local"] OR [mh "Safety"] OR (adverse NEXT effect*):ab,ti,kw OR (adverse NEXT health NEXT care NEXT event*):ab,ti,kw OR (adverse NEXT side NEXT effect*):ab,ti,kw OR (after-effect*):ab,ti,kw OR (adverse NEXT event*):ab,ti,kw OR (complication*):ab,ti,kw OR (contraindication*):ab,ti,kw OR (metastasis):ab,ti,kw OR (neoplasm* recurrence*):ab,ti,kw OR (precaution*):ab,ti,kw OR (safe*):ab,ti,kw OR (side NEXT effect*):ab,ti,kw OR (tumor NEXT recurrence*):ab,ti,kw OR (tumour NEXT recurrence*):ab,ti,kw

**#4**
#1 AND #2 AND #3 = **184** -> Search Limits = CENTRAL Trials

**Update:**
**#4**
#1 AND #2 AND #3 = **46** with Cochrane Library publication date from Sep 2023 to Dec 2024, in Trials

**EMBASE (Ovid)**, 28-10-2022, **Update:** 24-09-2024

**1**
exp Neoplasm/ OR exp Leukemia/ OR cancer.ti,ab,kw. OR leucaemia.ti,ab,kw. OR leucemia.ti,ab,kw. OR leukaemia.ti,ab,kw. OR leukemia.ti,ab,kw. OR malignan*.ti,ab,kw. OR myeloma*.ti,ab,kw. OR metast*.ti,ab,kw. OR neoplas*.ti,ab,kw. OR oncology.ti,ab,kw. OR tumor*.ti,ab,kw. OR tumour*.ti,ab,kw.

**2**
exp Acupressure/ OR exp Cupping Therapy/ OR Musculoskeletal Manipulation/ OR Manual Lymphatic Drainage/ OR exp Massage/ OR Orthopedic Manipulation/ OR Reflexology/ OR Rolfing/ OR Shiatsu/ OR Soft Tissue Therapy/ OR acupressure.ti,ab,kw. OR cupping.ti,ab,kw. OR (lymph ADJ drainage ADJ massage).ti,ab,kw. OR (manual ADJ lymph ADJ drainage).ti,ab,kw. OR (manual ADJ lymphatic ADJ drainage).ti,ab,kw. OR massage.ti,ab,kw. OR (musculoskeletal ADJ manipulation*).ti,ab,kw. OR (osteopathic ADJ manipulat*).ti,ab,kw. OR (osteopathic ADJ treatment).ti,ab,kw. OR (osteopathic ADJ visceral ADJ manipulation).ti,ab,kw. OR (osteopathic ADJ visceral ADJ treatment).ti,ab,kw. OR reflexological.ti,ab,kw. OR reflexology.ti,ab,kw. OR rolfing.ti,ab,kw. OR shiatsu.ti,ab,kw. OR (soft ADJ tissue ADJ therapy).ti,ab,kw. OR (tui ADJ na).ti,ab,kw. OR tuina.ti,ab,kw. OR (zone ADJ therapy).ti,ab,kw.

**3**
Complication/ OR exp Contraindication/ OR exp Metastasis/ OR Safety/ OR Tumor Recurrence/ OR Adverse Event/ OR si.fs. OR (adverse ADJ effect*).ti,ab,kw. OR (adverse ADJ health ADJ care ADJ event*).ti,ab,kw. OR (adverse ADJ side ADJ effect*).ti,ab,kw. OR (after ADJ effect*).ti,ab,kw. OR (adverse ADJ event*).ti,ab,kw. OR complication*.ti,ab,kw. OR contraindication*.ti,ab,kw. OR metastasis.ti,ab,kw. OR (neoplasm* ADJ recurrence*).ti,ab,kw. OR precaution*.ti,ab,kw. OR safe*.ti,ab,kw. OR (side ADJ effect).ti,ab,kw. OR (tumor ADJ recurrence*).ti,ab,kw. OR (tumour ADJ recurrence*).ti,ab,kw.

**4**
1 AND 2 AND 3 = **1.392**

**Update:**
**4**
1 AND 2 AND 3 = 1.614

**5**
limit 4 to dc=20221001-20231231 = **171**

**ISRCTN Registry (UK)**, 04-11-2022, **Update:** 26-09-2024

**47 results for** (cancer* OR carcinom* OR leucaemia* OR leucemia* OR leukaemia* OR leukemia* OR malignan* OR metast* OR myeloma* OR neoplas* OR oncology OR tumor* OR tumour*) AND (acupressure OR cupping OR "lymph drainage massage" OR "Manipulation, Orthopedic" OR "manual lymph drainage" OR "manual lymphatic drainage" OR massage OR "musculoskeletal manipulation*" OR "osteopathic anipulat*" OR "osteopathic treatment" OR "osteopathic visceral manipulation" OR "osteopathic visceral treatment" OR reflexological OR reflexology OR rolfing OR shiatsu OR "soft tissue therapy" OR "Therapy, Soft Tissue" OR "tui na" OR tuina OR "zone therapy")

**Update:**

**2 results for** (cancer* OR carcinom* OR leucaemia* OR leucemia* OR leukaemia* OR leukemia* OR malignan* OR metast* OR myeloma* OR neoplas* OR oncology OR tumor* OR tumour*) AND (acupressure OR cupping OR "lymph drainage massage" OR "Manipulation, Orthopedic" OR "manual lymph drainage" OR "manual lymphatic drainage" OR massage OR "musculoskeletal manipulation*" OR "osteopathic anipulat*" OR "osteopathic treatment" OR "osteopathic visceral manipulation" OR "osteopathic visceral treatment" OR reflexological OR reflexology OR rolfing OR shiatsu OR "soft tissue therapy" OR "Therapy, Soft Tissue" OR "tui na" OR tuina OR "zone therapy") within Date applied: from: 01/10/2022 Remove filter Date applied: to: 31/12/2024

**PEDro - The Physiotherapy Evidence Database**, 04-11-2022, **Update:** 24-09-2024

| | |
|---|---|
| Abstract & Title: Massage ***#3***<br>Subdiscipline: oncology<br>When Searching: Match all search terms (AND) | **Update:**<br>Abstract & Title: Massage ***#3***<br>Subdiscipline: oncology<br>When Searching: Match all search terms (AND)<br>New records added since: 01/10/2022 |
| **#3**<br>01 "adverse effect" = 0<br>02 "adverse effects" = 2<br>03 "adverse health care event" = 0<br>04 "adverse health care events" = 0<br>05 "adverse side effect" = 0<br>06 "adverse side effects" = 0<br>07 "after-effect" = 0<br>08 "after-effects" = 0<br>09 "adverse event" = 0<br>10 "adverse events" = 3<br>11 complication = 2<br>12 complications = 6<br>13 contraindication = 0<br>14 contraindications = 0<br>15 metastasis = 0<br>16 "neoplasm recurrence" = 0<br>17 "neoplasm recurrences" = 0<br>18 precaution = 0<br>19 precautions = 1<br>20 safe = 5<br>21 safely = 2<br>22 safety = 3<br>23 "side effect" = 2<br>24 "side effects" = 7<br>25 "tumor recurrence" = 0<br>26 "tumor recurrences" = 0<br>27 "tumour recurrence" = 0<br>28 "tumour recurrences" = 0 | **#3**<br>01 "adverse effect" = 0<br>02 "adverse effects" = 0<br>03 "adverse health care event" = 0<br>04 "adverse health care events" = 0<br>05 "adverse side effect" = 0<br>06 "adverse side effects" = 0<br>07 "after-effect" = 0<br>08 "after-effects" = 0<br>09 "adverse event" = 0<br>10 "adverse events" = 0<br>11 complication = 0<br>12 complications = 0<br>13 contraindication = 0<br>14 contraindications = 0<br>15 metastasis = 0<br>16 "neoplasm recurrence" = 0<br>17 "neoplasm recurrences" = 0<br>18 precaution = 0<br>19 precautions = 0<br>20 safe = 0<br>21 safely = 0<br>22 safety = 0<br>23 "side effect" = 0<br>24 "side effects" = 0<br>25 "tumor recurrence" = 0<br>26 "tumor recurrences" = 0<br>27 "tumour recurrence" = 0<br>28 "tumour recurrences" = 0 |
| Total: **33** | Total: **0** |

**PubMed (NLM)**, 28-10-2022, **Update:** 25-09-2024

**#1**
Neoplasms[mh] OR cancer*[tiab] OR carcinom*[tiab] OR leucaemia*[tiab] OR leucemia*[tiab] OR leukaemia*[tiab] OR leukemia*[tiab] OR malignan*[tiab] OR metasta*[tiab] OR myeloma*[tiab] OR neoplas*[tiab] OR oncology[tiab] OR tumor*[tiab] OR tumour*[tiab]

**#2**
Acupressure[mh] OR Cupping Therapy[mh] OR Musculoskeletal Manipulations[mh:noexp] OR Manual Lymphatic Drainage[mh] OR Manipulation, Orthopedic[mh] OR Massage[mh] OR Therapy, Soft Tissue[mh] OR acupressure[tiab] OR cupping[tiab] OR "lymph drainage massage"[tiab] OR "manual lymph drainage"[tiab] OR "manual lymphatic drainage"[tiab] OR massage[tiab] OR "musculoskeletal manipulation*"[tiab] OR "osteopathic manipulat*"[tiab] OR "osteopathic treatment"[tiab] OR "osteopathic visceral manipulation"[tiab] OR "osteopathic visceral treatment"[tiab] OR reflexological[tiab] OR reflexology[tiab] OR rolfing[tiab] OR shiatsu[tiab] OR "soft tissue therapy"[tiab] OR "tui na"[tiab] OR tuina[tiab] OR "zone therapy"[tiab]

**#3**
Contraindications[mh] OR Neoplasm Metastasis[mh] OR Neoplasm Recurrence, Local[mh] OR Safety[mh] OR adverse effects[sh] OR "adverse effect*"[tiab] OR "adverse health care event*"[tiab] OR "adverse side effect*"[tiab] OR "after-effect*"[tiab] OR "adverse event*"[tiab] OR complication*[tiab] OR contraindication*[tiab] OR metastasis[tiab] OR "neoplasm recurrence*"[tiab] OR precaution*[tiab] OR safe*[tiab] OR "side effect*"[tiab] OR "tumor recurrence*"[tiab] OR "tumour recurrence*"[tiab]

**#4**
#1 AND #2 AND #3 = **757**

**Update:**
**#4**
#1 AND #2 AND #3 = 855

**#5**
#4 AND (("2022/10/01"[CRDT] : "3000"[CRDT] OR "2022/10/01"[EDAT] : "3000"[EDAT] OR "2022/10/01"[MHDA] : "3000"[MHDA])) = **109**

**Web of Science - SCI Expanded/SSCI (Clarivate)**, 28-10-2022, **Update:** 24-09-2024

**#1**
TS=(cancer* OR carcinom* OR leucaemia* OR leucemia* OR leukaemia* OR leukemia* OR malignan* OR metast* OR myeloma* OR neoplas* OR oncology OR tumor* OR tumour*)

**#2**
TS=(acupressure OR cupping OR "lymph drainage massage" OR "Manipulation, Orthopedic" OR "manual lymph drainage" OR "manual lymphatic drainage" OR massage OR "musculoskeletal manipulation*" OR "osteopathic manipulat*" OR "osteopathic treatment" OR "osteopathic visceral manipulation" OR "osteopathic visceral treatment" OR reflexological OR reflexology OR rolfing OR shiatsu OR "soft tissue therapy" OR "Therapy, Soft Tissue" OR "tui na" OR tuina OR "zone therapy")

**#3**
TS=("adverse effect*" OR "adverse health care event*" OR "adverse side effect*" OR "after-effect*" OR "adverse event*" OR complication* OR contraindication* OR metastasis OR "neoplasm recurrence*" OR "Neoplasm Recurrence, Local" OR precaution* OR safe* OR "side effect*" OR (tumor NEAR/2 recurrence*) OR (tumour NEAR/2 recurrence*))

**#4**
#1 AND #2 AND #3 = **1.448**

**Update:**
**#4**
#1 AND #2 AND #3 = 1.485

**#5**
#4 AND LD=(2022-10-01/2024-12-31) = **213**
