# Supplementary material for: Harms of massage in persons with cancer or receiving treatment for cancer: A systematic review and meta-analysis
Source: PLoS One. 2026 Aug 5;21(8):e0351878. doi: 10.1371/journal.pone.0351878 (PMC13440881; doi:10.1371/journal.pone.0351878)
Supplement: Supplementary file 2 [file pone.0351878.s011.pdf]

Supplementary file 11 - Sensitivity Analyses

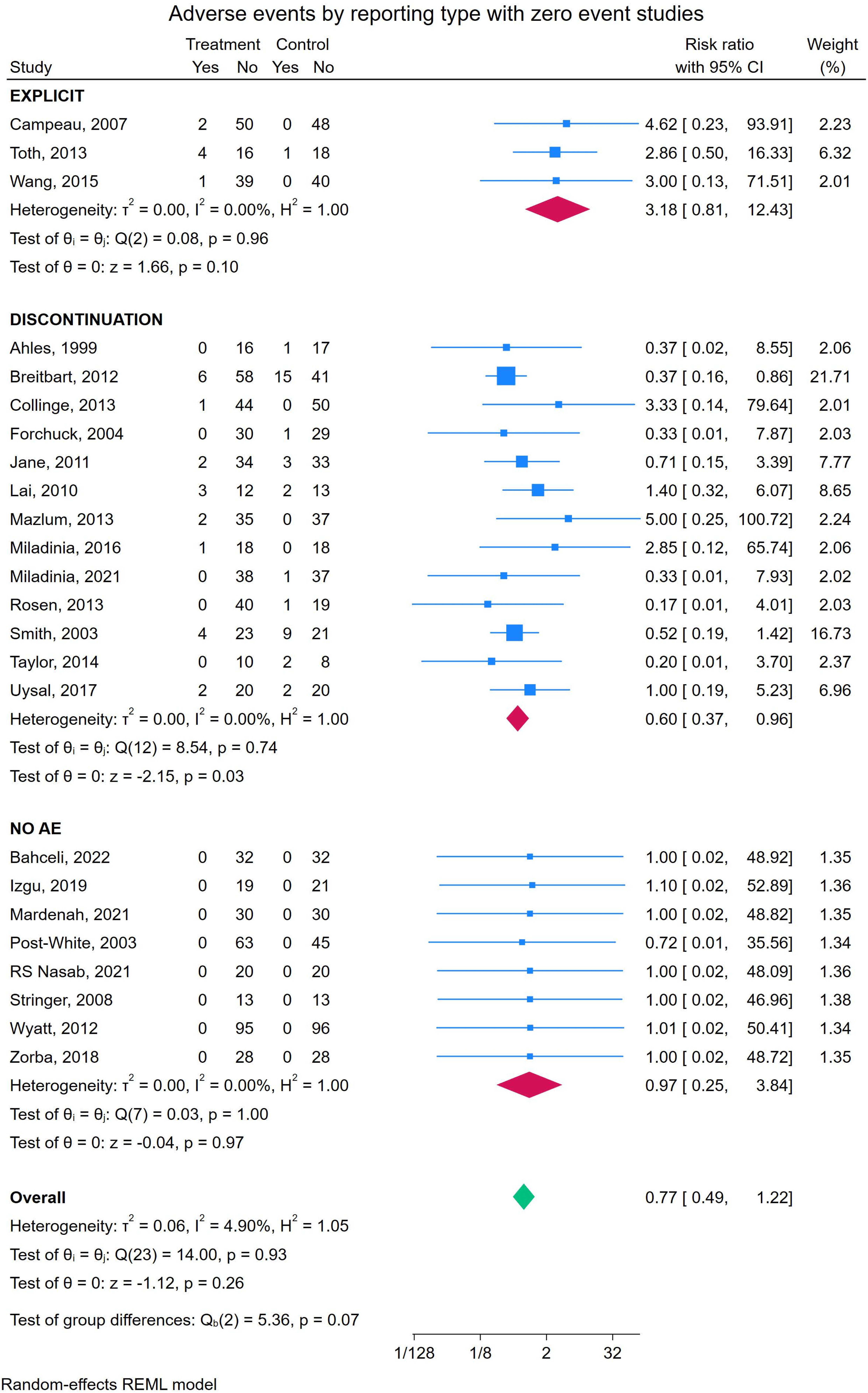
Adverse events by reporting type with zero event studies
Study
Treatment Yes No
Control Yes No
Risk ratio with 95% CI
Weight (%)
EXPLICIT
Campeau, 2007 2 50 0 48 4.62 [0.23, 93.91] 2.23
Toth, 2013 4 16 1 18 2.86 [0.50, 16.33] 6.32
Wang, 2015 1 39 0 40 3.00 [0.13, 71.51] 2.01
Heterogeneity: τ^2 = 0.00, I^2 = 0.00%, H^2 = 1.00 3.18 [0.81, 12.43]
Test of $\theta_i = \theta_j$: Q(2) = 0.08, p = 0.96
Test of θ = 0: z = 1.66, p = 0.10
DISCONTINUATION
Ahles, 1999 0 16 1 17 0.37 [0.02, 8.55] 2.06
Breitbart, 2012 6 58 15 41 0.37 [0.16, 0.86] 21.71
Collinge, 2013 1 44 0 50 3.33 [0.14, 79.64] 2.01
Forchuck, 2004 0 30 1 29 0.33 [0.01, 7.87] 2.03
Jane, 2011 2 34 3 33 0.71 [0.15, 3.39] 7.77
Lai, 2010 3 12 2 13 1.40 [0.32, 6.07] 8.65
Mazlum, 2013 2 35 0 37 5.00 [0.25, 100.72] 2.24
Miladinia, 2016 1 18 0 18 2.85 [0.12, 65.74] 2.06
Miladinia, 2021 0 38 1 37 0.33 [0.01, 7.93] 2.02
Rosen, 2013 0 40 1 19 0.17 [0.01, 4.01] 2.03
Smith, 2003 4 23 9 21 0.52 [0.19, 1.42] 16.73
Taylor, 2014 0 10 2 8 0.20 [0.01, 3.70] 2.37
Uysal, 2017 2 20 2 20 1.00 [0.19, 5.23] 6.96
Heterogeneity: τ^2 = 0.00, I^2 = 0.00%, H^2 = 1.00 0.60 [0.37, 0.96]
Test of $\theta_i = \theta_j$: Q(12) = 8.54, p = 0.74
Test of θ = 0: z = -2.15, p = 0.03
NO AE
Bahceli, 2022 0 32 0 32 1.00 [0.02, 48.92] 1.35
Izgu, 2019 0 19 0 21 1.10 [0.02, 52.89] 1.36
Mardenah, 2021 0 30 0 30 1.00 [0.02, 48.82] 1.35
Post-White, 2003 0 63 0 45 0.72 [0.01, 35.56] 1.34
RS Nasab, 2021 0 20 0 20 1.00 [0.02, 48.09] 1.36
Stringer, 2008 0 13 0 13 1.00 [0.02, 46.96] 1.38
Wyatt, 2012 0 95 0 96 1.01 [0.02, 50.41] 1.34
Zorba, 2018 0 28 0 28 1.00 [0.02, 48.72] 1.35
Heterogeneity: τ^2 = 0.00, I^2 = 0.00%, H^2 = 1.00 0.97 [0.25, 3.84]
Test of $\theta_i = \theta_j$: Q(7) = 0.03, p = 1.00
Test of θ = 0: z = -0.04, p = 0.97
Overall 0.77 [0.49, 1.22]
Heterogeneity: τ^2 = 0.06, I^2 = 4.90%, H^2 = 1.05
Test of $\theta_i = \theta_j$: Q(23) = 14.00, p = 0.93
Test of θ = 0: z = -1.12, p = 0.26
Test of group differences: $Q_b(2)$ = 5.36, p = 0.07
1/128
1/8
2
32
Random-effects REML model

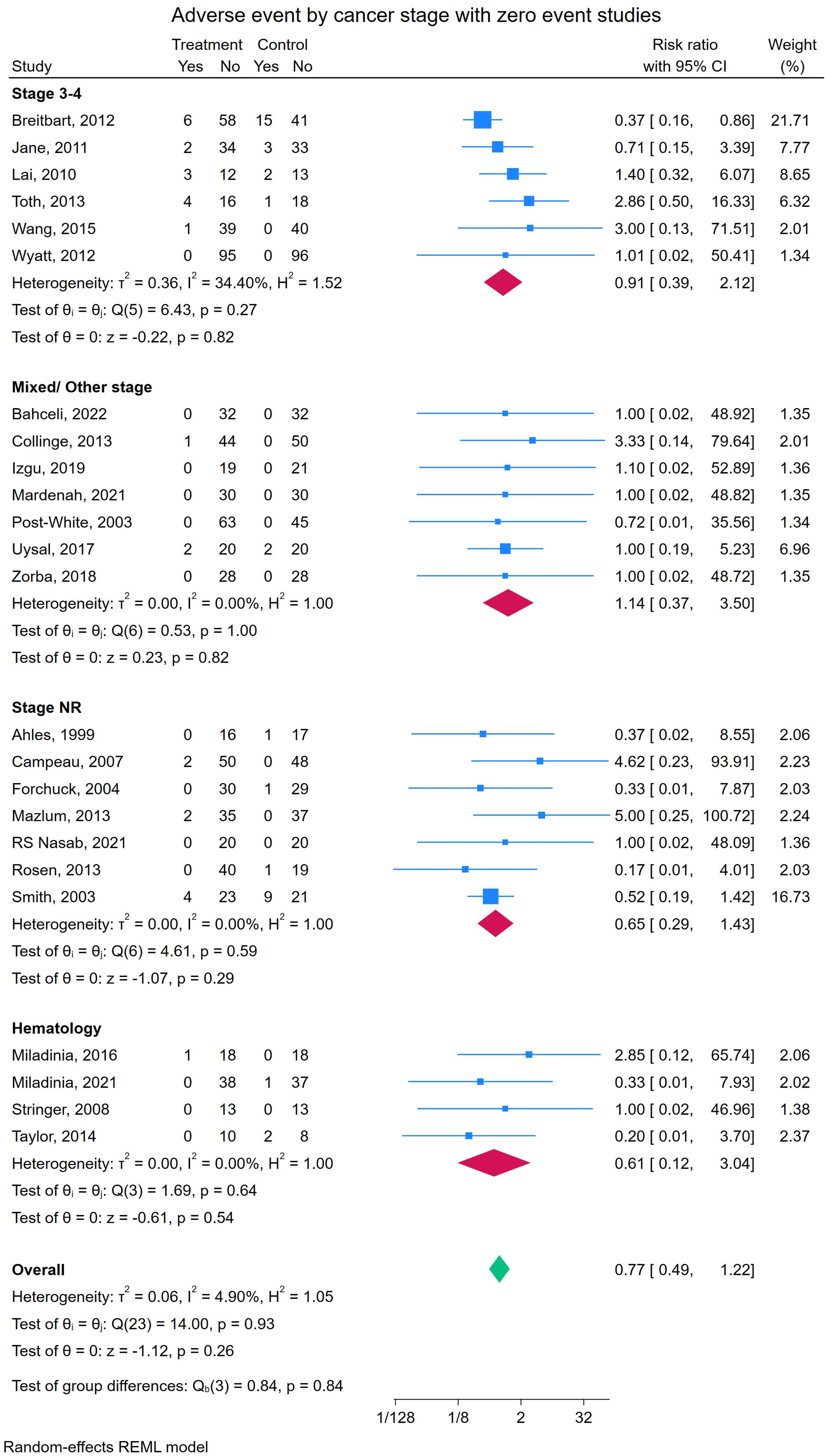
Adverse event by cancer stage with zero event studies
Study | Treatment Yes | Treatment No | Control Yes | Control No | Risk ratio with 95% CI | Weight (%)
Stage 3-4
Breitbart, 2012 6 58 15 41 0.37 [0.16, 0.86] 21.71
Jane, 2011 2 34 3 33 0.71 [0.15, 3.39] 7.77
Lai, 2010 3 12 2 13 1.40 [0.32, 6.07] 8.65
Toth, 2013 4 16 1 18 2.86 [0.50, 16.33] 6.32
Wang, 2015 1 39 0 40 3.00 [0.13, 71.51] 2.01
Wyatt, 2012 0 95 0 96 1.01 [0.02, 50.41] 1.34
Heterogeneity: τ² = 0.36, I² = 34.40%, H² = 1.52 0.91 [0.39, 2.12]
Test of θi = θj: Q(5) = 6.43, p = 0.27
Test of θ = 0: z = -0.22, p = 0.82
Mixed/ Other stage
Bahceli, 2022 0 32 0 32 1.00 [0.02, 48.92] 1.35
Collinge, 2013 1 44 0 50 3.33 [0.14, 79.64] 2.01
Izgu, 2019 0 19 0 21 1.10 [0.02, 52.89] 1.36
Mardenah, 2021 0 30 0 30 1.00 [0.02, 48.82] 1.35
Post-White, 2003 0 63 0 45 0.72 [0.01, 35.56] 1.34
Uysal, 2017 2 20 2 20 1.00 [0.19, 5.23] 6.96
Zorba, 2018 0 28 0 28 1.00 [0.02, 48.72] 1.35
Heterogeneity: τ² = 0.00, I² = 0.00%, H² = 1.00 1.14 [0.37, 3.50]
Test of θi = θj: Q(6) = 0.53, p = 1.00
Test of θ = 0: z = 0.23, p = 0.82
Stage NR
Ahles, 1999 0 16 1 17 0.37 [0.02, 8.55] 2.06
Campeau, 2007 2 50 0 48 4.62 [0.23, 93.91] 2.23
Forchuck, 2004 0 30 1 29 0.33 [0.01, 7.87] 2.03
Mazlum, 2013 2 35 0 37 5.00 [0.25, 100.72] 2.24
RS Nasab, 2021 0 20 0 20 1.00 [0.02, 48.09] 1.36
Rosen, 2013 0 40 1 19 0.17 [0.01, 4.01] 2.03
Smith, 2003 4 23 9 21 0.52 [0.19, 1.42] 16.73
Heterogeneity: τ² = 0.00, I² = 0.00%, H² = 1.00 0.65 [0.29, 1.43]
Test of θi = θj: Q(6) = 4.61, p = 0.59
Test of θ = 0: z = -1.07, p = 0.29
Hematology
Miladinia, 2016 1 18 0 18 2.85 [0.12, 65.74] 2.06
Miladinia, 2021 0 38 1 37 0.33 [0.01, 7.93] 2.02
Stringer, 2008 0 13 0 13 1.00 [0.02, 46.96] 1.38
Taylor, 2014 0 10 2 8 0.20 [0.01, 3.70] 2.37
Heterogeneity: τ² = 0.00, I² = 0.00%, H² = 1.00 0.61 [0.12, 3.04]
Test of θi = θj: Q(3) = 1.69, p = 0.64
Test of θ = 0: z = -0.61, p = 0.54
Overall 0.77 [0.49, 1.22]
Heterogeneity: τ² = 0.06, I² = 4.90%, H² = 1.05
Test of θi = θj: Q(23) = 14.00, p = 0.93
Test of θ = 0: z = -1.12, p = 0.26
Test of group differences: Qb(3) = 0.84, p = 0.84
1/128
1/8
2
32
Random-effects REML model

Adverse events by cancer treatment with zero event studies

Study	Treatment Yes	Treatment No	Control Yes	Control No	Risk ratio with 95% CI	Weight (%)
**Cancer treatment**						
Ahles, 1999	0	16	1	17	0.37 [0.02, 8.55]	2.06
Bahceli, 2022	0	32	0	32	1.00 [0.02, 48.92]	1.35
Campeau, 2007	2	50	0	48	4.62 [0.23, 93.91]	2.23
Collinge, 2013	1	44	0	50	3.33 [0.14, 79.64]	2.01
Forchuck, 2004	0	30	1	29	0.33 [0.01, 7.87]	2.03
Izgu, 2019	0	19	0	21	1.10 [0.02, 52.89]	1.36
Jane, 2011	2	34	3	33	0.71 [0.15, 3.39]	7.77
Mardenah, 2021	0	30	0	30	1.00 [0.02, 48.82]	1.35
Mazlum, 2013	2	35	0	37	5.00 [0.25, 100.72]	2.24
Miladinia, 2016	1	18	0	18	2.85 [0.12, 65.74]	2.06
Miladinia, 2021	0	38	1	37	0.33 [0.01, 7.93]	2.02
Post-White, 2003	0	63	0	45	0.72 [0.01, 35.56]	1.34
RS Nasab, 2021	0	20	0	20	1.00 [0.02, 48.09]	1.36
Rosen, 2013	0	40	1	19	0.17 [0.01, 4.01]	2.03
Smith, 2003	4	23	9	21	0.52 [0.19, 1.42]	16.73
Stringer, 2008	0	13	0	13	1.00 [0.02, 46.96]	1.38
Taylor, 2014	0	10	2	8	0.20 [0.01, 3.70]	2.37
Uysal, 2017	2	20	2	20	1.00 [0.19, 5.23]	6.96
Wyatt, 2012	0	95	0	96	1.01 [0.02, 50.41]	1.34
Zorba, 2018	0	28	0	28	1.00 [0.02, 48.72]	1.35
Heterogeneity: $\tau^2 = 0.00$, $I^2 = 0.00\%$, $H^2 = 1.00$					0.75 [0.43, 1.31]	
Test of $\theta_i = \theta_j$: $Q(19) = 7.58$, $p = 0.99$						
Test of $\theta = 0$: $z = -0.99$, $p = 0.32$						
**Cancer treatment unknown**						
Breitbart, 2012	6	58	15	41	0.37 [0.16, 0.86]	21.71
Lai, 2010	3	12	2	13	1.40 [0.32, 6.07]	8.65
Toth, 2013	4	16	1	18	2.86 [0.50, 16.33]	6.32
Wang, 2015	1	39	0	40	3.00 [0.13, 71.51]	2.01
Heterogeneity: $\tau^2 = 0.69$, $I^2 = 53.19\%$, $H^2 = 2.14$					1.06 [0.34, 3.33]	
Test of $\theta_i = \theta_j$: $Q(3) = 6.40$, $p = 0.09$						
Test of $\theta = 0$: $z = 0.10$, $p = 0.92$						
**Overall**					0.77 [0.49, 1.22]	
Heterogeneity: $\tau^2 = 0.06$, $I^2 = 4.90\%$, $H^2 = 1.05$						
Test of $\theta_i = \theta_j$: $Q(23) = 14.00$, $p = 0.93$						
Test of $\theta = 0$: $z = -1.12$, $p = 0.26$						
Test of group differences: $Q_b(1) = 0.27$, $p = 0.60$						

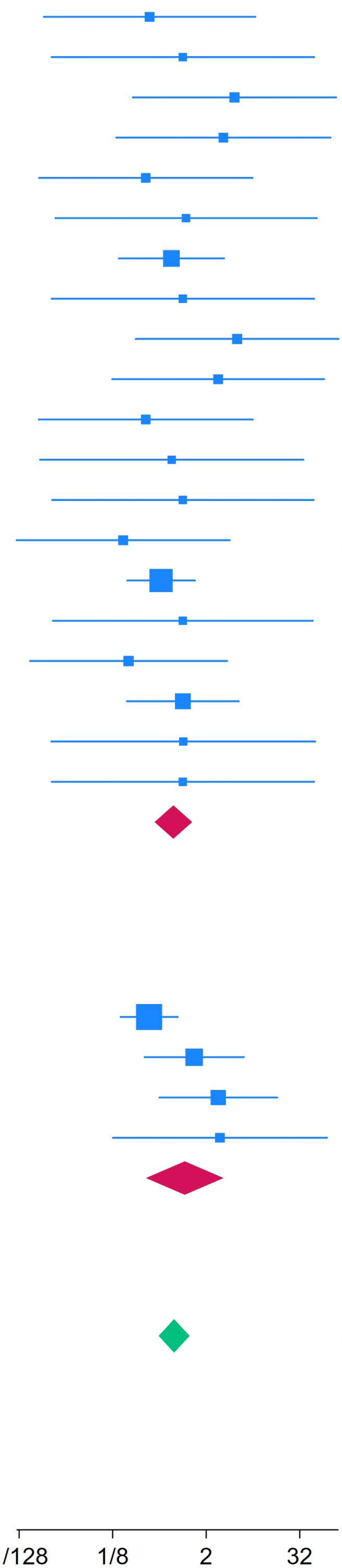

Random-effects REML model

Adverse event by ROB with zero event studies

Study	Treatment Yes	Treatment No	Control Yes	Control No	Risk ratio with 95% CI	Weight (%)
**High (ROB-I)**						
Zorba, 2018	0	28	0	28	1.00 [0.02, 48.72]	1.35
Heterogeneity: τ^2 = 0.00, I^2 = .%, H^2 = .					1.00 [0.02, 48.72]	
Test of $\theta_i = \theta_j$: Q(0) = 0.00, p = .						
Test of θ = 0: z = 0.00, p = 1.00						
**Poor**						
Breitbart, 2012	6	58	15	41	0.37 [0.16, 0.86]	21.71
Lai, 2010	3	12	2	13	1.40 [0.32, 6.07]	8.65
Heterogeneity: τ^2 = 0.52, I^2 = 58.25%, H^2 = 2.40					0.62 [0.17, 2.24]	
Test of $\theta_i = \theta_j$: Q(1) = 2.40, p = 0.12						
Test of θ = 0: z = -0.72, p = 0.47						
**Fair**						
Ahles, 1999	0	16	1	17	0.37 [0.02, 8.55]	2.06
Campeau, 2007	2	50	0	48	4.62 [0.23, 93.91]	2.23
Collinge, 2013	1	44	0	50	3.33 [0.14, 79.64]	2.01
Forchuck, 2004	0	30	1	29	0.33 [0.01, 7.87]	2.03
Mazlum, 2013	2	35	0	37	5.00 [0.25, 100.72]	2.24
Smith, 2003	4	23	9	21	0.52 [0.19, 1.42]	16.73
Taylor, 2014	0	10	2	8	0.20 [0.01, 3.70]	2.37
Toth, 2013	4	16	1	18	2.86 [0.50, 16.33]	6.32
Uysal, 2017	2	20	2	20	1.00 [0.19, 5.23]	6.96
Post-White, 2003	0	63	0	45	0.72 [0.01, 35.56]	1.34
RS Nasab, 2021	0	20	0	20	1.00 [0.02, 48.09]	1.36
Heterogeneity: τ^2 = 0.06, I^2 = 4.20%, H^2 = 1.04					0.93 [0.47, 1.82]	
Test of $\theta_i = \theta_j$: Q(10) = 7.58, p = 0.67						
Test of θ = 0: z = -0.21, p = 0.83						
**Moderate (ROB-I)**						
Wang, 2015	1	39	0	40	3.00 [0.13, 71.51]	2.01
Heterogeneity: τ^2 = 0.00, I^2 = .%, H^2 = .					3.00 [0.13, 71.51]	
Test of $\theta_i = \theta_j$: Q(0) = 0.00, p = .						
Test of θ = 0: z = 0.68, p = 0.50						
**Good**						
Jane, 2011	2	34	3	33	0.71 [0.15, 3.39]	7.77
Miladinia, 2016	1	18	0	18	2.85 [0.12, 65.74]	2.06
Miladinia, 2021	0	38	1	37	0.33 [0.01, 7.93]	2.02
Rosen, 2013	0	40	1	19	0.17 [0.01, 4.01]	2.03
Bahceli, 2022	0	32	0	32	1.00 [0.02, 48.92]	1.35
Izgu, 2019	0	19	0	21	1.10 [0.02, 52.89]	1.36
Mardenah, 2021	0	30	0	30	1.00 [0.02, 48.82]	1.35
Stringer, 2008	0	13	0	13	1.00 [0.02, 46.96]	1.38
Heterogeneity: τ^2 = 0.00, I^2 = 0.00%, H^2 = 1.00					0.73 [0.26, 2.00]	
Test of $\theta_i = \theta_j$: Q(7) = 1.89, p = 0.97						
Test of θ = 0: z = -0.62, p = 0.54						
**Excellent**						
Wyatt, 2012	0	95	0	96	1.01 [0.02, 50.41]	1.34
Heterogeneity: τ^2 = 0.00, I^2 = .%, H^2 = .					1.01 [0.02, 50.41]	
Test of $\theta_i = \theta_j$: Q(0) = -0.00, p = .						
Test of θ = 0: z = 0.01, p = 1.00						
**Overall**					0.77 [0.49, 1.22]	
Heterogeneity: τ^2 = 0.06, I^2 = 4.90%, H^2 = 1.05						
Test of $\theta_i = \theta_j$: Q(23) = 14.00, p = 0.93						
Test of θ = 0: z = -1.12, p = 0.26						
Test of group differences: $Q_b(5)$ = 1.01, p = 0.96						

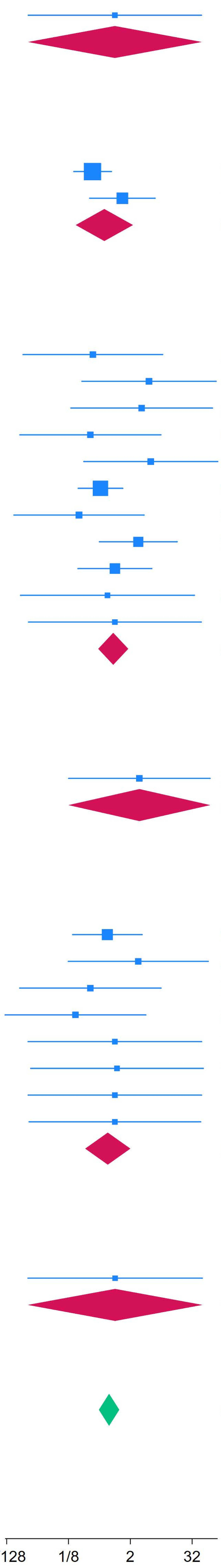

Random-effects REML model
