# Supplementary material for: Harms of massage in persons with cancer or receiving treatment for cancer: A systematic review and meta-analysis
Source: PLoS One. 2026 Aug 5;21(8):e0351878. doi: 10.1371/journal.pone.0351878 (PMC13440881; doi:10.1371/journal.pone.0351878)
Supplement: Supplementary file 3 [file pone.0351878.s012.pdf]

Supplementary file 12:

Sensitivity analyses of adverse event reporting score ≥5

A) For primary studies
B) Including studies with 0 adverse events

A

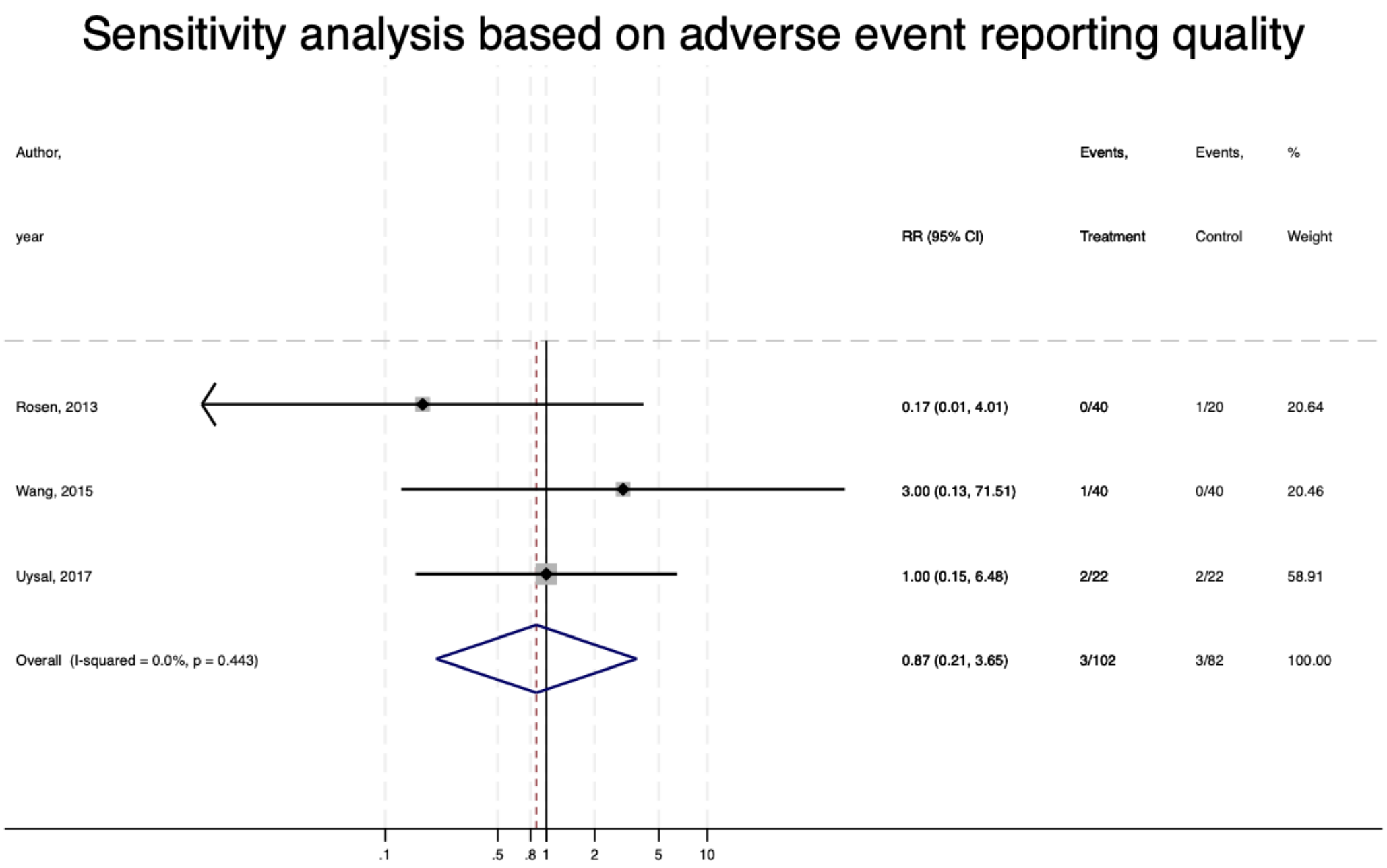
Sensitivity analysis based on adverse event reporting quality
Author, year
RR (95% CI)
Events, Treatment
Events, Control
% Weight
Rosen, 2013
0.17 (0.01, 4.01)
0/40
1/20
20.64
Wang, 2015
3.00 (0.13, 71.51)
1/40
0/40
20.46
Uysal, 2017
1.00 (0.15, 6.48)
2/22
2/22
58.91
Overall (I-squared = 0.0%, p = 0.443)
0.87 (0.21, 3.65)
3/102
3/82
100.00
.1
.5
.8
1
2
5
10

B

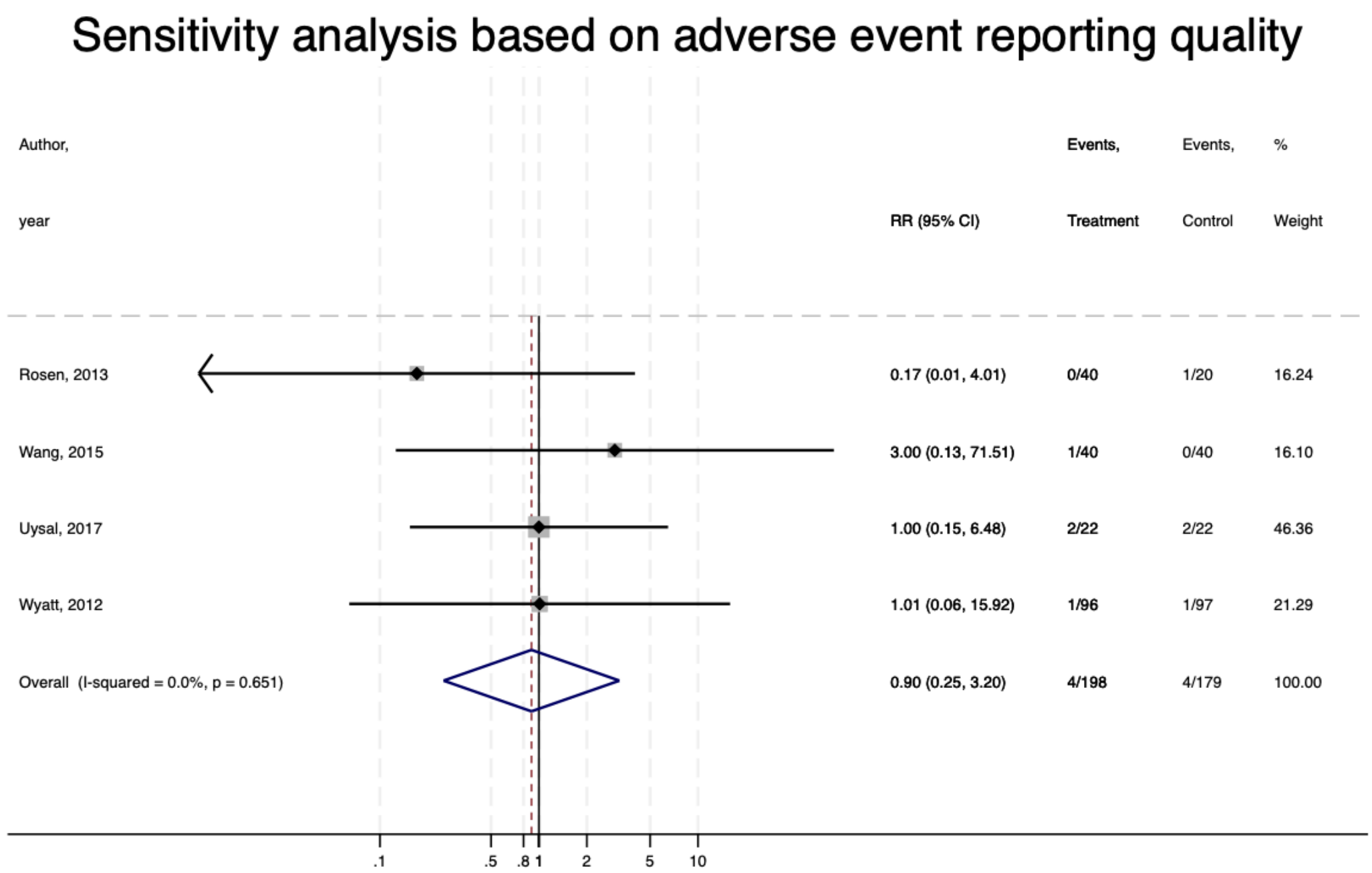
Sensitivity analysis based on adverse event reporting quality
Author, year
RR (95% CI)
Events, Treatment
Events, Control
% Weight
Rosen, 2013
0.17 (0.01, 4.01)
0/40
1/20
16.24
Wang, 2015
3.00 (0.13, 71.51)
1/40
0/40
16.10
Uysal, 2017
1.00 (0.15, 6.48)
2/22
2/22
46.36
Wyatt, 2012
1.01 (0.06, 15.92)
1/96
1/97
21.29
Overall (I-squared = 0.0%, p = 0.651)
0.90 (0.25, 3.20)
4/198
4/179
100.00
.1
.5
.8
1
2
5
10
