# Supplementary material for: Harms of massage in persons with cancer or receiving treatment for cancer: A systematic review and meta-analysis
Source: PLoS One. 2026 Aug 5;21(8):e0351878. doi: 10.1371/journal.pone.0351878 (PMC13440881; doi:10.1371/journal.pone.0351878)
Supplement: Supplementary file 4 [file pone.0351878.s013.pdf]

Supplementary file 13 - Funnel Plots

A All adverse events
B Discontinuations
C Receiving cancer treatment

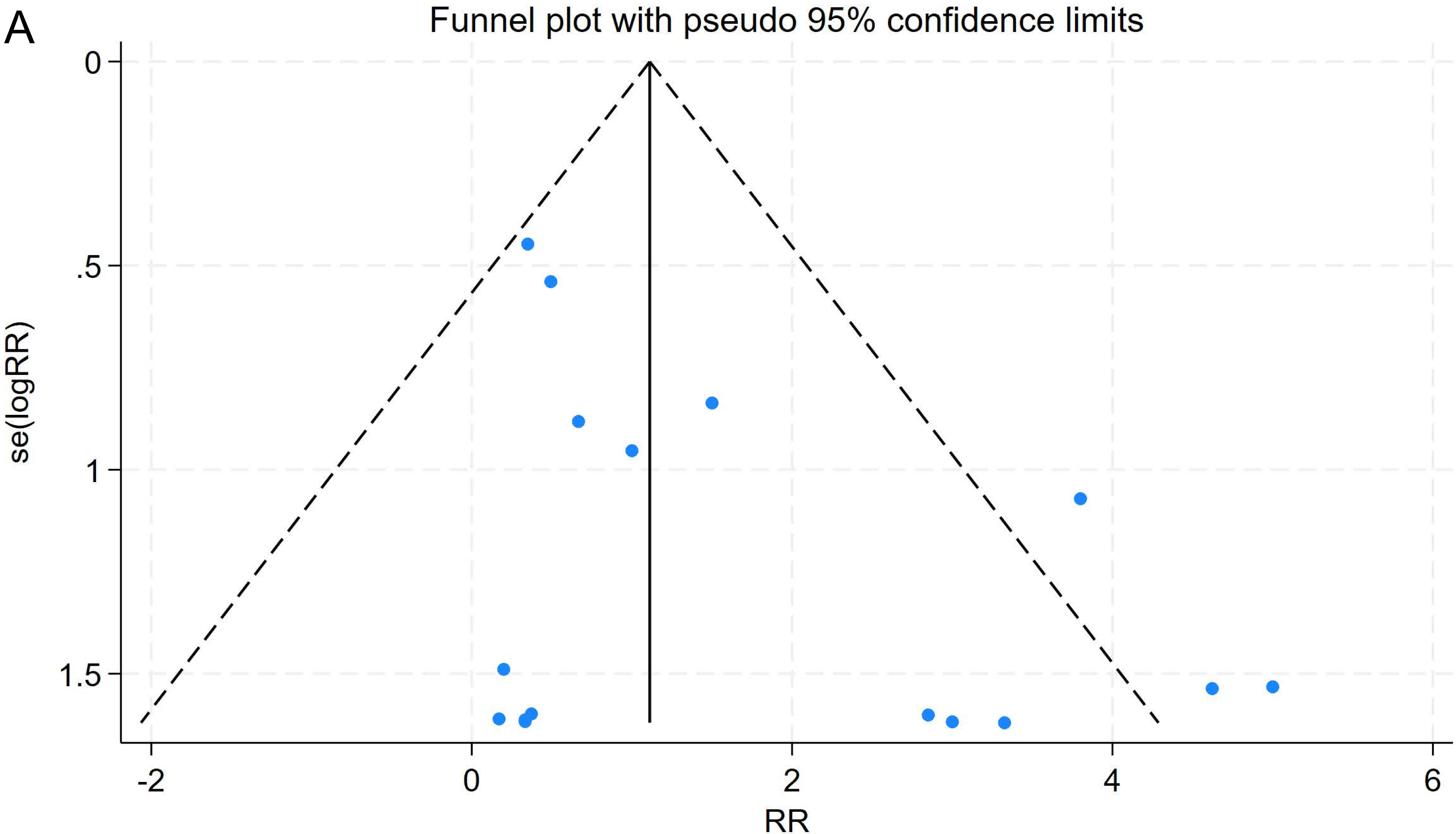
A
Funnel plot with pseudo 95% confidence limits
0
.5
1
1.5
se(logRR)
-2
0
2
4
6
RR

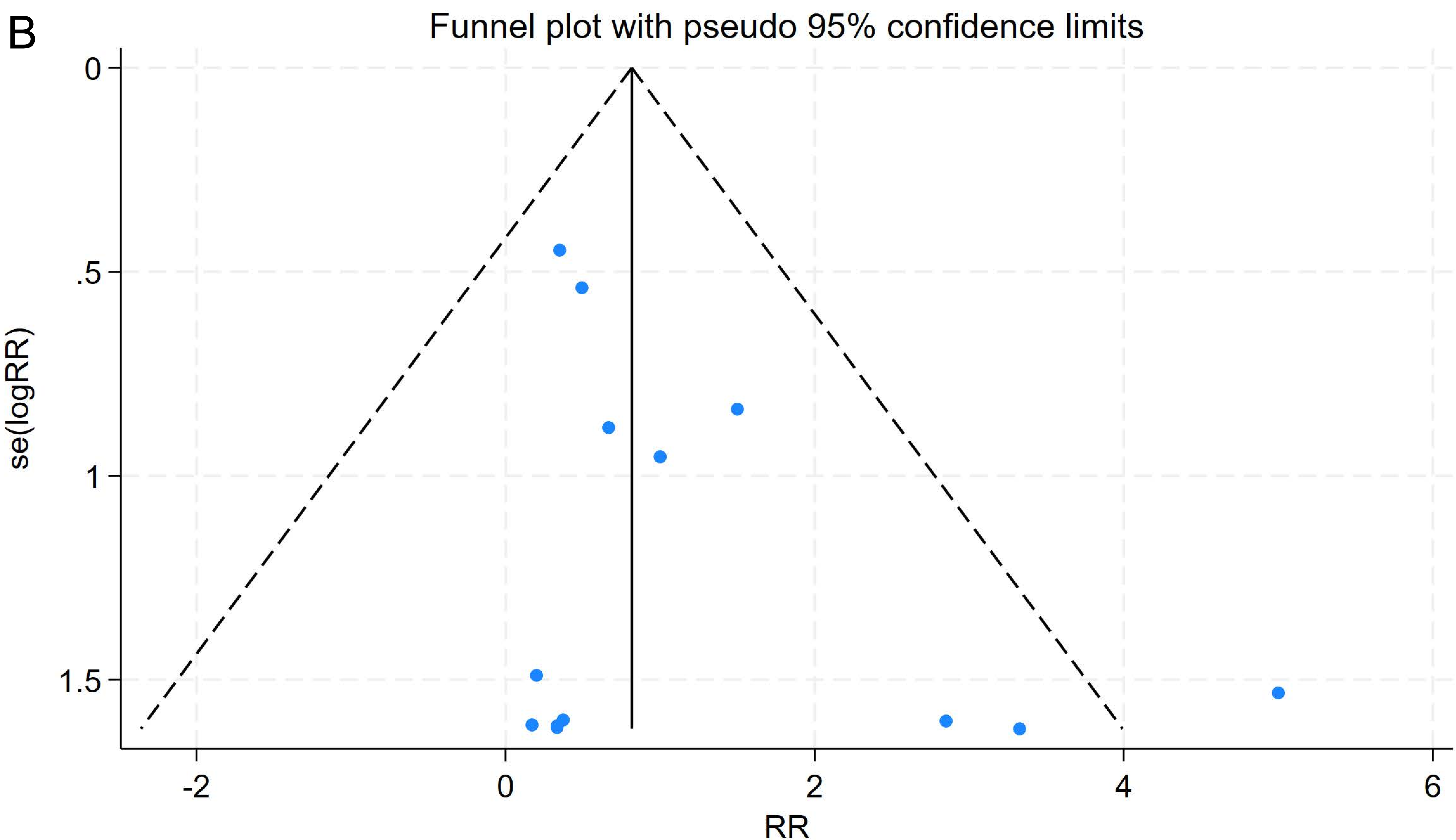
B
Funnel plot with pseudo 95% confidence limits
se(logRR)
0
.5
1
1.5
-2
0
2
4
6
RR

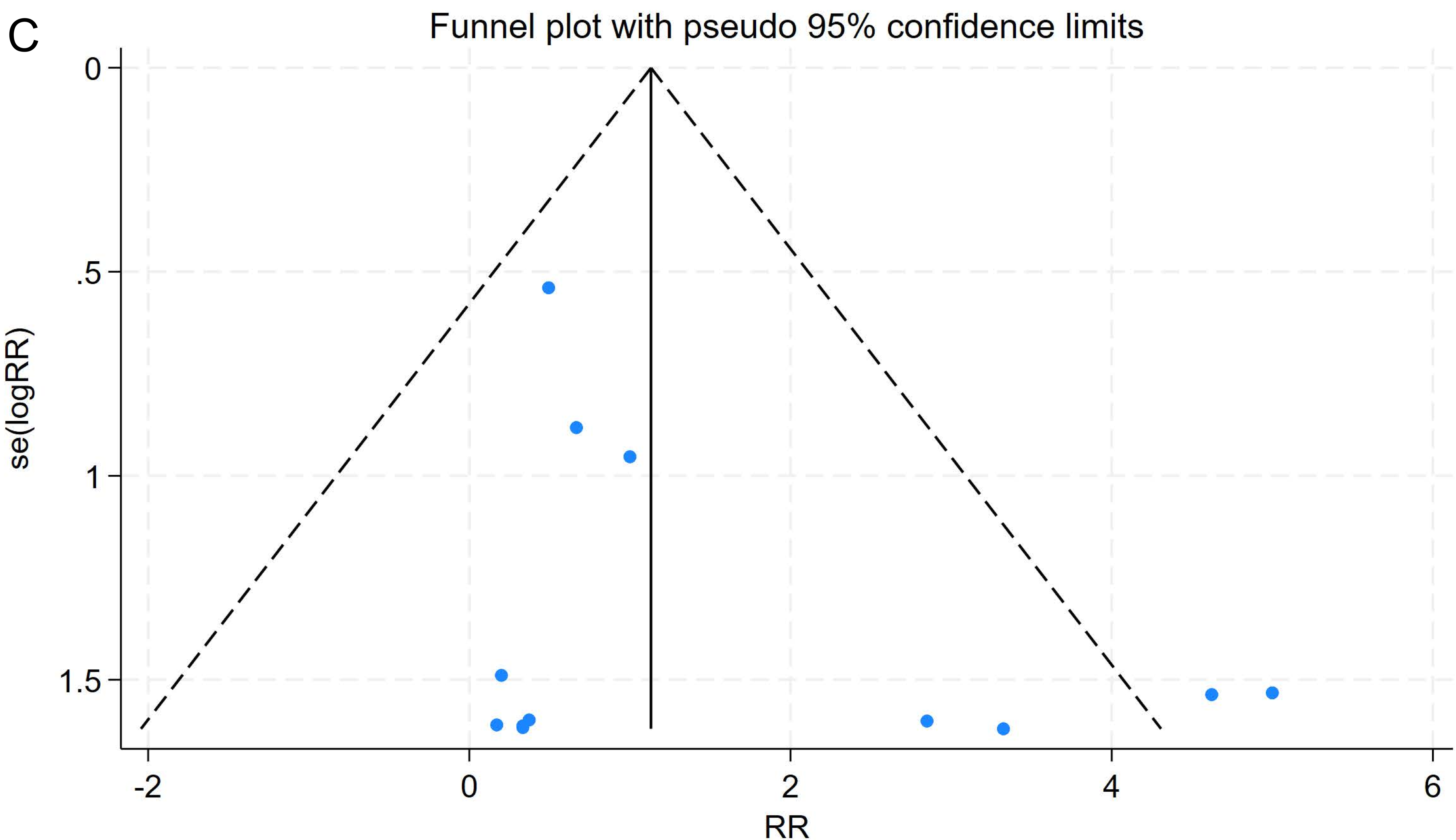
C
Funnel plot with pseudo 95% confidence limits
0
.5
1
1.5
se(logRR)
-2
0
2
4
6
RR
