# Supplementary material for: Harms of massage in persons with cancer or receiving treatment for cancer: A systematic review and meta-analysis
Source: PLoS One. 2026 Aug 5;21(8):e0351878. doi: 10.1371/journal.pone.0351878 (PMC13440881; doi:10.1371/journal.pone.0351878)
Supplement: Supplementary file 6 [file pone.0351878.s003.docx]

# Supplementary information 3. Specification of target randomised controlled trials and confounders in risk of bias assessment of non-randomized trials

The Risk Of Bias In Non-randomized Studies – of Interventions (ROBINS-I) assessment tool

(version for cohort-type studies)

**Version 19 September 2016**

This work is licensed under a [Creative Commons Attribution-NonCommercial-NoDerivatives 4.0 International License](http://creativecommons.org/licenses/by-nc-nd/4.0/).

# ROBINS-I tool (Stage I): At protocol stage

## Specify the review question

| Participants | Patients with evidence of cancer (with or without treatment) or receiving neoadjuvant or adjuvant treatment for cancer (radiotherapy, chemotherapy, immunotherapy) or were within six weeks of cancer-related surgery. |
| --- | --- |
| Experimental intervention | Massage plus standard of care |
| Comparator | Standard of care |
| Outcomes | Adverse events, lost to follow-up, discontinuation or withdrawal due to adverse event, outcomes related to cancer proliferation or metastases |

## List the confounding domains relevant to all or most studies

Socioeconomic status and geographical area of residence
