# Supplementary material for: Harms of massage in persons with cancer or receiving treatment for cancer: A systematic review and meta-analysis
Source: PLoS One. 2026 Aug 5;21(8):e0351878. doi: 10.1371/journal.pone.0351878 (PMC13440881; doi:10.1371/journal.pone.0351878)
Supplement: Supplementary file 7 [file pone.0351878.s005.docx]

| **Study details** | | **Participant characteristics** | | | **Massage intervention** | | | | **Adverse events** | |
| --- | --- | --- | --- | --- | --- | --- | --- | --- | --- | --- |
| Author, year  Setting  N | Study aim | Criteria specific to cancer & treatment/  Sex/  Age | Cancer Type/  Stage | Treatment during intervention | Type /  Region | Intensity/  Delivery | Time, frequency & duration | Comparator | Type/  AE leading to discontinuation or withdrawl | Severity or seriousness/  Last FU |
| Randomized controlled trials | | | | | | | | | | |
| Ahles, 1999 [51]  Hospital (inpatient)  United States  N = 34 | Examine the feasibility of massage in a bone marrow transplant setting and to test the efficacy of massage therapy in reducing symptoms of physical and psychological distress. | Patients scheduled for autologous bone marrow transplantation.  Sex  Male: 8  Female: 26  Age (yrs)  M: 41.3±9.4^a^  UC: 42.3±9.5^a^ | Type  BC: 22  AML: 2  NHL: 6  HL: 2  Other: 2  Stage  NR | Chemo: 34  SC trans: 34 | Type  Swedish effleurage and petrissage  Region  Shoulders, neck, face, and scalp | Intensity  Gentle  Steady pressure to acupressure points  Delivery  Trained, healing-arts specialist  > 10 yrs experience | 20 min, 9x’ s within 3 wks | UC | Type  AE: NR  Discontinuation  UC: 1 medical complications | Severity or seriousness  NR  Last FU  Immediately PI |
| Bahceli, 2022 [52]  Hospital (outpatient)  Turkey  N = 64 | Investigate the effect of Slow-stroke back massage on the level of fatigue. | Patients with BC receiving adjuvant  AC/ CAF.  Excluded: open wounds and edema of the upper limbs and back; bleeding or clotting issues  Sex  Male: 0  Female: 64  Age (yrs)  M: 49.6±9.1 ^a^  UC: 47.8±10.3^a^ | Type  BC: 64  Stage  ll: 55  lll: 9 | Chemo: all | Type  Slow-stroke  Region  Back | Intensity  Gentle  Delivery  Nurse practitioner certified in massage | 20 min, 3x ‘s in 3 wk interval | UC | Type  AE: NR  Discontinuation  “No participants discontinued due to unexpected AE” | Severity or seriousness  NR  Last FU  Within 1 day PI |
| Breitbart, 2012 [53]  Hospital (outpatient)  United States  N = 120 | Compare Individual meaning-centered psychotherapy with a therapeutic massage intervention. | Patients with stage lll or lV solid tumor cancer or NHL  Sex  Male: 48  Female: 72  Age (yrs)  All: 54.4±11.6^a^  range [25-82] | Type  BC: 31  Colon: 19  Pancreatic: 11  Ovarian: 10  Lung: 4  Other: 45  Stage  lll: 40  lV: 80 | NR | Type  Adaptation of Swedish massage for frail patients.  Region  Whole body or region | Intensity  Gentle  Delivery  Massage therapist | 60 min, 7x’s over 7 wks | P: Individual meaning-centered psychotherapy | Type  AE: NR  Discontinuation  M: 6  P: 15  both groups “due to worsening of illness or death” | Severity or seriousness  NR  Last FU  2 mo post-intervention |
| Campeau, 2007 [54]  Hospital (outpatient)  Canada  N = 100 | Assess the impact of massage therapy on intermediate-term anxiety levels in patients with cancer. | Patients receiving radiotherapy  Excluded: low platelet count (< 100,000/iL)  Sex  Male: 32  Female: 68  Age (yrs)  M: 60 [38-81]^b^  AT: 58 [35-85]^b^ | Type  BC: 36  H&N: 26  Gyn: 12  Prostate: 10  Other: 16  Stage  NR | RT: all | Type  Chair massage- general effleurage.  Adjusted to avoid massage /pressure in treated area.  Region  Back, arms, hands, neck, scalp | Intensity  NR  Delivery  Massage therapist | 15 min, daily for 10 consecutive days | AT: Met with massage therapist | Type  M:  Presyncope: 2  One episode due to pelvic pain, likely exacerbated by massage position.  One episode med.-induced  hypotension.  Discontinuation  NR | Severity or seriousness  NR  Last FU  Immediately PI |
| Collinge, 2013 [55]  Cancer support and advocacy centers (outpatient)  United States  N = 97 | Determine impact of caregiver-administered massages on symptoms and side effects. | Cancer patients experiencing side effects of any type or stage of cancer or its treatments  Sex  Male: 23  Female: 74  Age (yrs)  All: 54.7±11.6^a^ | Type  BC: 44  Lung: 10  GI: 10  Blood: 9  Gyn: 8  Brain: 4  Prostate: 3  Thyroid: 3  Kidney: 2  Other: 4  Stage  l: 9  ll: 24  lll: 16  lV: 35  Remission: 5  Unknown: 8 | Mixed: Chemo, RT & post-surgery- Not clear how many | Type  Manual techniques for comfort and relaxation. Acupressure for pain, anxiety and nausea.  Region  All or some; head, neck, shoulders, back, feet, and hands | Intensity  NR  Delivery  Caregiver (instructed through DVD and written material) | 18 ± 12 min.  3.6 ± 4.1 x’s/ wk for 4 wks | AT: Caregiver reading to patient | Type  No AE  Discontinuation  M:  Hospitalized: 1 | Severity or seriousness  NR  Last FU  4 wks PI |
| Forchuck, 2004 [56]  Canada  N = 60 | Determine effect of  massage delivered by significant other following lymph  node dissection on  comfort and fewer functional constraints. | Patients with BC scheduled for ALND  Sex  Male: 0  Female: 60  Age (yrs)  All: mean 56  (range 21-78) | Type  Breast: 60  Stage  NR | Post-surgery: all | Type  (Swedish) as per techniques described.  Region  Neck, hand, shoulder | Intensity  Gentle- can increase gradually as comfortable  Delivery  Significant others (taught by nurse) | 10 min suggested, as often as needed for 1 wk  (mean 2.69) | UC | Type  No AE  Discontinuation  UC:  Death: 1 | Severity or seriousness  NR  Last FU  4 mo post-intervention |
| Izgu, 2019 [57]  Hospital (outpaient)  Turkey  N = 40 | Examine effect of classical massage on CIPN | Patients receiving adjuvant paclitaxel for BC  Exclude: lesions on hands & feet, bleeding or coagulation issues  Sex  Male: 0  Female: 40  Age (yrs)  M: 44.5 ± 10.7^a^  UC: 47.0±9.6^a^ | Type  BC: 40  Stage  ll: 16  lll: 24 | Mixed: post-surgery and chemo: all | Type  (Swedish) Effleurage, petrissage and friction with Vaseline  Region  Hands and fee | Intensity  Superficial friction.  Other NR  Delivery  Massage therapist | 20 min feet  10 min hands, 1x /wk for 12 wks (prior to infusion) | UC | Type  AE: NR  Discontinuation:  None due to adverse events. | Severity or seriousness  NA  Last FU  1 mo post-intervention |
| Jane, 2011 [58]  Hospital (inpatient)  Taiwan  N = 72 | Compare the efficacy of massage to social attention control on pain intensity, mood status, muscle relaxation, and sleep quality. | Cancer patients with bone metastases with at least moderate pain  Exclude: experiencing allodynia; or any physical condition for which massage was contraindicated, fx thrombocytopenia, active spinal cord compression syndrome, or deep vein thrombosis  Sex  Male: 30  Female: 42  Age (yrs)  All: 49.9±10.6^a^ | Type  Lung: 24  BC: 17  GI: 10  GU: 8  Other: 13  Stage Metastatic: all | Receiving treatment (type not specified): 39 | Type  (Swedish) Effleurage, petrissage, compression, nerve stroke  Region  Full body  Avoidance of sites with superficial tumors, infection or hyperesthesia | Intensity  Light to moderate. Light/gentle over bone metastases.  Delivery  Advanced cancer nurses with short course in massage | 40 min ± 4, for 3 consecutive days | AT: Social attention | Type  No AE  Discontinuation  M:  Physical distress: 1  Disease progression: 1  AT:  Physical distress: 2  Feeling stressed: 1 | Severity or seriousness  NA  Last FU  1-day post-intervention |
| Kutner, 2008 [59]  Hospice  United States  N = 380  (Merged with Ghesquiere, 2019 [81]) | Evaluate efficacy of massage for decreasing pain and symptom distress and improving quality of life. | Patients with advanced cancer with at least moderate pain  Exclude: anticoagulant therapy, known platelet count <10,000 or unstable spine.  Sex  Male: 148  Female: 232  Age (yrs)  M: 65.2±14.4^a^  ST: 64.2±14.4^a^ | Type  Lung: 96  BC: 63  Pancreas: 35  Colorectal: 29  Prostate: 21  Other: 136  Stage  lll & lV: 380 | NR | Type  (Swedish) Efflerurage (65% of time) and petrissage (35%)) and myofascial trigger point.  Region  Neck & upper back (app. 80% of the time).  Sites of inflammation/infection, hyperesthesia, injury, surgery, ports, catheters, deep vein thrombosis and tumors were avoided. | Intensity  Light /gentle.  (modified in persons with skin fragility, postural limitations, edema, osteoporosis or bone metastasis)  Delivery  Massage therapist (one person per site) | 30 min, 6 x’s during 2 wks | ST: Simple touch:  both hands on the participant for 3 minutes at each of the locations bilaterally | Type  M:  Respiratory infection: 1  GI bleed: 1  Death: 26  ST:  Heart failure: 1  Seizure: 1  Fracture: 1  Pain control: 3  Death: 33  Discontinuation  One AE in each group resulted in discontinuation. Not specified which. | Severity or seriousness  M: 2 serious  ST: 6 serious  (Classification of seriousness not clear)  Last FU  1.5 wks post-intervntion |
| Lai, 2010 [60]  Hospital (inpatient)  Hong Kong  N = 32 | Verify the effect of aroma massage on constipation in advanced  cancer patients. | Patients with advanced cancer with constipation  Sex  Male: 24  Female: 8  Exclude: Colostomy, tumor or open wound at proposed area of massage, acute surgical conditions (fx peritonitis, intestinal obstruction)  Age (yrs): All  31-50: 6  51-70: 13  71-90: 13 | Type  Lung: 18  NP: 5  BC: 2  Corpus: 1  Prostate: 1  Pancreas: 1  UP: 4  Stage Advanced: all | NR | Type  Abdominal massage  Region  Abdomen | Intensity  NR  Delivery  Author plus 4 nurses (trained by the author) | 15-20 min, daily for 5 consecutive days | Two arms:  - UC  - AR (aroma + massage) | Type  AE: NR  Discontinuation  M:  Increased use of laxatives: 2  Shortness of breath: 1 Increased fatigue: 1  UC:  Increased use of laxatives: 1  AR:  Increased use of laxatives: 2 | Severity or seriousness  NR  Last FU  Immediately PI |
| Mardaneh, 2021 [61]  Hospital (outpatient)  Iran  N = 60 | Evaluate the effects of Thai massage on comfort and symptoms. | Female cancer patients receiving chemo  Sex  Male: 0  Female: 60  Age (yrs)  M: 43.5±8.0^a^  UC: 48.53±30.0^a^ | Type  Lymphoma: 7  BC: 22  Pancreas: 2  Uterus: 10  Ovary: 10  Stomach: 2  Colon: 5  Leukemia: 2  Stage  “No end stage” | Chemo: all | Type  Thai massage using  effleurage and petrissage  Region  Back, legs, arms, abdomen, chest, and face | Intensity  Firm but gentle  Delivery  Female massage therapist | 30-45 min,  2x /wk  10 sessions (at least 1 day apart from chemo, usually given once every 2 wks.) | UC | Type  AE: NR  Discontinuation  No participants excluded. *A priori* “excluded” if participants developed severe cancer‑related complications, or metastatic cancer or severe physical or mental problems | Severity or seriousness  NR  Last FU  Immediately PI |
| Mazlum, 2013 [62]  Hospital (inpatient) Iran  N = 70 | Assess the effect of massage on chemo-induced nausea and vomiting in pediatric cancer | Hospitalized pediatric (4-18 yrs) cancer patients receiving chemo  Exclude: GI & nervous system cancer, Wilm’s tumor, any other mass or surgery in the abdominal area, sore or injury in the massage area, metastasis, radiation, port-A cath  Sex  NR  Age (yrs)  NR | Type NR  Stage  NR | Chemo: all | Type  Swedish with effleurage, petrissage, friction and tapping  Region  NR | Intensity  Mild to moderate  Delivery  Therapist skilled in massage therapy | 20 min, 3x’s  24 hr & 30 min before chemo, and 24 hrs after chemo | AT: Therapist stayed with the children in the timeslots for massage. | Type  AE: NR  Discontinuation  M:  Fever and neutropenia before chemo: 2 | Severity or seriousness  NR  Last FU  24 hrs post-intervention |
| Miladinia, 2016 [63]  Hospital  Iran  N = 35 | Evalutate the effect of Slow-stroke back massage on anxiety in pediatrics with leukemia. | Pediatric patients with chronic leukemia  Sex  Male: 19  Female: 16  Age (yrs)  M: 11.0+2.1^a^  AT: 12.6+1.9^a^ | Type  Chronic Leukemia: 35  Stage  NR | Chemo: all | Type  Slow stroke  Region  Back (Base of skull to sacrum) | Intensity  Surface strokes  Delivery  NR | 5 min, 3x/wk for 4 wks | AT: Talking with nurse | Type  AE: NR  Discontinuation  M:  Deterioration in condition: 1 | Severity or seriousness  NR  Last FU  2 wks post-intervention |
| Miladinia, 2021 [64]  Hospital  Iran  N = 104 | Compare massage and music to standard care to reduce CRF and CRP in adults with (AML and ALL | Patients receiving chemo for AML or ALL with pain and fatigue  Exclude: platelet count < 15,000  Sex  Male: 49  Female: 55  Age (yrs)  M: 38.3+11.0^a^  AT: 39.2+10.9^a^  MT^:^ 36.6+11.6^a^ | Type  AML & ALL: 104  Stage  NR | Chemo: all | Type  Slow stroke  Region  Back (Base of skull to sacrum) | Intensity  Light  Delivery  Nurse (trained by massage specialist) | 15 min, 3x/wk for 4 wks | Two arms:  -AT: Talking with nurse  -Music therapy (MT) | Type  AE: NR  Discontinuation  AT:  Deterioration in condition: 1 | Severity or seriousness  NR  Last FU  2 wks post-intervention |
| Moradi, 2020 [65]  Hospital (outpatient)  Iran  N = 96 | Examine effect of aromatherapy by inhalation and massage on radiotherapy-induced fatigue | Cancer patients receiving radiotherapy. No ulcer, redness.  Sex  Male: 46  Female: 50  Age (yrs)  M: 43.9+12.5^a^  UC: 46.6+11.9^a^  AR: 42.9+12.4^a^ | Type  NR  Stage  NR | RT: all | Type  (Swedish) Effleurage massage with aroma  Region  Back-shu points  (paravertebral muscles) | Intensity  Light  Delivery  Massage therapist | 30 min, 3 x’s  (every other day post-radiotherapy) | Two arms:  -UC  -AR (aroma inhalation) | Type  AE: NR  Discontinuation  M:  Hospitalization and death: 2  UC:  Hospitalization: 2  AR:  Lack of cooperation or death: 5 | Severity or seriousness  NR  Last FU  Immediately PI |
| RS Nasab, 2021 [66]  Hospital  Iran  N = 59 | Investigate the effectiveness of abdominal massage with and without Salvia officinalis on nausea and vomiting in patients with cancer undergoing chemo. | Patients with cancer receiving chemo  Exclude: colostomy, acute abdominal surgery complications (fx intestinal obstruction,  Peritonitis) local tumor or open wound in massage area  Sex  Male: 50%  Female: 50%  Age (yrs)  M: 54.5+12.6^a^  UC: 52.4+12.4^a^  AR: 55.3+13.8^a^ | Type  BC: 15  Blood: 6  Respiratory: 8  GI: 13  GU: 7  Other: 10  Stage  NR | Chemo: all | Type  Abdominal swedish-type including effleurage, vibration and kneeding  Region  Abdomen | Intensity  NR  Delivery  Patient’s companion | 15 min, 2x’s/day for 3 days | Two arms:  -UC  - AR + Swedish massage | Type  No AE  Discontinuation  None due to adverse events | Severity or seriousness  NA  Last FU  1 wk post-intervention |
| Rosen, 2013 [67]  Hospital  United States  N = 60 | Assess feasibility and effectiveness of the massage in  reducing perioperative pain and anxiety. | Patients with cancer undergoing surgical placement of a vascular access device.  Exclude: Clotting or coagulation issue, open wounds in massage area.  Sex  Male: 28  Female: 32  Age (yrs)  M: 52.3[28-74]^b^  AT: 56.5[23-84]^b^ | Type  BC: 18  Colon:15  Pancreatic: 5  Lymphoma: 5  Prostate: 2  Ovarian: 2  Leukemia: 1  Other: 12  Stage  NR | Post-surgery: all | Type  (Swedish) Effleurage, compression and cranial balance and cradle hold  Region  *Pre-surgery:*  upper-extremity, hand and feet Gentle rocking whole body.  *Post-surgery*: Cranial and face, neck, shoulder, arms hand and feet. | Intensity  Gentle  Delivery  Massage therapist | 20 min, 2x’s (pre- & post-surgery) | AT (Structured attention) | Type  No AE  Discontinuation  AT:  Pain: 1 | Severity or seriousness  NR  Last FU  1 day post-intervention |
| Smith, 2003 [68]  Hospital (inpatient)  United States  N = 88 | Investigate the effect of touch therapies on selected health outcomes during bone marrow transplant. | Cancer patients receiving bone marrow transplantation  Sex  Male: 22  Female: 66  Age (yrs)  All: 46±106^a^  range [19-67] | Type  Lymphoma: 15  BC: 49  Other: 21  Stage  NR | SC trans: all | Type  Swedish effleurage and petrissage  Region  NR (modified to avoid IV sites and wounds) | Intensity  Light ( with low platelets)  Delivery  Registered nurse, massage therapist | 30 min, every 3^rd^ day until engraftment | Two arms:  -AT (visit)  -Therapeutic touch (TT) | Type  AE: NR  Discontinuation  Death  M: 4  AT: 9  TT: 6 | Severity or seriousness  NR  Last FU  2 wks PI |
| Stringer, 2008 [69]  Hospital (inpatient)  United Kingdom  N = 39 | Establish safety of massage with and without essential oils and assess physiological and psychological benefit of massage in isolated hemotology patients. | Isolated hematology oncology patients with a platelet count ≥15,000  Exclude: thrombosis or phlebitis  Sex  Male: 16  Female: 23  Age (yrs)  M: 57 [22-70]^b^  R: 50 [22-71]^b^  AR: 47 [19-75]^b^ | Type  Hemotology: all  Stage  NR | Chemo: 20  Chemo + SC trans: 18  Other: 1 | Type  (Swedish) Effleurage with unscented oil  Region  Patient choice | Intensity  Light  Delivery  Hematology nurse with diploma in AR | 20 min, 1x | Two arms:  -R, Rest  -AR + effleurage | Type  No AE  Discontinuation  None due to adverse event | Severity or seriousness  NA  Last FU  1 day post-intervention |
| Taylor, 2014 [82]  Hospital (inpatient)  United States  N = 16 | Examine effects of massage intervention on quality of life and treatment-related symptoms in patients with AML | Patients with AML  Sex  Male: 9  Female: 7  Age (yrs)  M: 52±17.5^a^  UC: 46.4±14.3^a^ | Type  AML: all  Stage  NR | Chemo: all | Type  Swedish (primarily effleurage & petrissage)  Region  Head, neck, shoulders, legs, arms, back, abdomen, feet, hands, (avoid central & IV sites) | Intensity  Gentle. No deep tissue.  Delivery  Massage therapist | 50 min, 3 x’s/wk for 7 wks | UC | Type  AE: NR  Discontinuation  UC:  “Became too ill”: 2 | Severity or seriousness  NR  Last FU  Immediately PI |
| Toth, 2013 [70]  Hospital (treatment in patient’s home)  United States  N = 39 | Assess benefit of home delivered massage to manage pain, anxiety, alertness, quality of life and sleep for patients with metastatic cancer. | Patients with metastatic cancer  Sex  Male: 7  Female: 32  Age (yrs)  All: 55±11^a^ | Type  BC: 22  Colon: 5  Pancreatic: 3  Ovarian: 3  Stage  Metastatic: all | NR | Type  Swedish and non-swedish gliding/effleurage, gentle kneading/petrissage, compression, gentle stretching, rocking, light myofascial release, and use of acupressure points as well as craniosacral holds  Region  Any region (avoiding area of known met.) | Intensity  No deep. Average pressure 2/5. (restricted to gentle if platelets <50,000 on a given day).  Delivery  Massage therapist | 15-45 min, 3x’s during 1 wk | Two arms:  -UC  -No touch: hands app 12 inches over body | Type  M:  Hospitalized due to acute renal failure - thought to be secondary to CT contrast): 1  Hospitalized due to dyspnea & pain (due to new pulmonary met.): 1  Shortness of breath (after 2nd massage): 1  Increased pain (after 2nd massage): 1  UC:  Death: 1  Discontinuation  Not reported | Severity or seriousness  NR  Last FU  3 wks post-intervention |
| Uysal, 2017 [71]  Hospital  Turkey  N = 60 | Determine the effect of 2, foot massage methods on symptom control | Patients with colorectal cancer who received chemoradiotherapy  Exclude: fever, sores and thrombosis in area of massage  Sex  Male: 30  Female: 30  Age (yrs)  M: 60.6±8.1^a^  UC: 60.4±8.6^a^  R: 60.4±8.6^a^ | Type  Colorectal: all  Stage  ll: 18  lll: 42 | Chemo-RT: all | Type  (Swedish) Effleurage, petrissage, friction, vibration  Region  Feet | Intensity  NR  Delivery  Researcher | 10 min / foot, 2 x’s / wk for 5 wks | Two arms:  -UC  - R reflexology | Type  AE: NR  Discontinuation:  M:  Severe diarrhea: 1  Radiation dermatitis: 1  UC:  Reduced neutrophile: 1  Radiation dermatitis: 1  R:  Reduced thrombocytes: 1 | Severity or seriousness  NR  Last FU  Immediately PI |
| Wyatt, 2012 [72]  Oncology clinics (treatments performed at home)  United States  N = 286 | Evaluate the safety and efficacy of reflexology vs massage | Receiving treatment for advanced stage breast cancer  Sex  Male: 0  Female: 286  Age (yrs)  M: 54.8±11.2^a^  UC: 57.3±11.8^a^  R: 55.3±9.4^a^ | Type  BC: all  Stage  lll & lV, recurrence or metastasis: all | Chemo: all | Type  Lay foot massage- same areas as R  Region  Feet | Intensity  Superficial  Delivery  Lay women taught foot massage | 30 min, 1 x /wk for 4 wks | Two arm:  -UC  -R  (reflexology) | Type  No AE  Discontinuation  None due to adverse events | Severity or seriousness  NA  Last FU  3 wks post-intervention |
| Randomized cross-over trial | | | | | | | | | | |
| Kim, 2024 [80]  Hospital  South Korea  N = 59 | Assess effectiveness of an electric massage chair in preventing chemo-induced nausea and vomiting | Receiving high emetic risk chemo (HEC) og moderate emetic risk chemo (MEC) for solid tumor.  Exclude: disseminated or extensive bone metastasis or taking anticoagulants  Sex  Male: 36  Female: 23  Age (yrs)  All: 55[IQR 52 to 68] | Type  GI: 3  Colorectal: 39  BC: 15  Other: 2  Stage  NR | Chemo: all | Type  Chair massage  Region  Neck, back, calf, foot | Intensity  Gentle  Delivery  Massage chair (Bodyfriend Inc.) | 20 min, 2 x /wk for 1 wk | UC | Type  Only reported for whole group- 80 AE’s reported, all related  to chemo.  No injuries related to massage chair or interrupted therapy.  Discontinuation  No instance of attrition due to toxicities- all completed. | Severity or seriousness  15 AE’s grade 3 or 4. (Classification of grade not clear**)**  Last FU  10 days post-intervention |
| Post-White, 2003 [73]  Chemo clinics (outpatient)  United States  N = 230 | Determine effectiveness of massage and healing in reducing symptoms of anxiety, mood disturbance, pain,  nausea, and fatigue and increasing relaxation and satisfaction with care. | Cancer patients receiving chemo with rating ≥ 3 of pain, nausea, or fatigue (0 to 10).  Sex  Male: 32  Female: 198  Age (yrs)  All: 54.7±11.2^a^ | Type  Breast: 114  Gyn: 25  GI: 29  Hematology: 15  Lung: 8  GU: 6  Other: 7  Stage  l: 25  ll: 55  lll: 34  lV: 10  Unstaged: 15 | Chemo: all | Type  Swedish with effleurage, petrissage, friction/rub  Region  Whole body | Intensity  Gentle (adapted to individual tolerance)  Delivery  Nurses certified as massage therapist | 45 min, 1 x /wk for 4 wks | -Presence  -Healing touch | Type  No AE  Discontinuation  None due to adverse events after intervention start | Severity or seriousness  NA  Last FU  Immediately PI |
| Quasi-experimental trial | | | | | | | | | | |
| DeOliveira, 2018 [74]  Hospital  Brazil  N = 106  (Merged with DeOliveira 2017 & 2014 [75,76]) | Evaluate effects of a treatment program with MLD vs supervised exercise. | Women undergoing radical mastectomy  Sex  Male: 0  Female: 106  Age (yrs)  M: <55 yrs: 24  55+ yrs: 29  AE: <55 yrs: 22  55+ yrs: 31 | Type  BC: 106  Stage  l:1 ll: 26  lll & lV: 75 | Post-surgery: all | Type  MLD  Region  Lymph nodes contralateral axillary region and ipsilateral inguinal region and limb ipsilateral to surgery. | Intensity  Gentle  Delivery  Experienced physiotherapist | 40 min, 2 x /wk for 30 days | Active exercise | Type  AE: NR  Discontinuation  M:  Death: 19  Active Exercise:  Death: 17 | Severity or seriousness  NA  Last FU  30 mo post-intervention |
| Park, 2015 [77]  Hospital (inpatient) South Korea  N = 48 | Analyze effects of foot bath vs foot massage in CIPN | Stomach or colon cancer receiving chemo with grade 2 or 3 CIPN  Sex  Male: 29  Female: 19  Age (yrs)  M: 60.8 ±9.0^a^  FB: 57.0 ±11.5^a^ | Type  Stomach: 39  Colorectal: 9  Stage  lll: 21 lV: 27 | Chemo: all | Type  Foot massage  Region  Feet | Intensity  Gentle  Delivery  Researcher & caregiver after instruction from massage therapist | 30 min, 8 x’s over 2 wks | FB, Foot bath | Type  AE: NR  Discontinuation  FB:  Fever & leukocytopenia: 3  Dizziness: 1 | Severity or seriousness  NR  Last FU  Immediately PI |
| Wang, 2015 [78]  Hospital (inpatient)  Taiwan  N = 80 | Test effect of abdominal massage in reducing malignant ascites symptoms, in end-stage cancer patients. | Hospitalized patients with end-stage cancer and malignant ascites  Exclude: generalized infection, spontaneous bacterial peritonitis, platelet count <100,000; abdominal pain > 4 /10  Sex  Male: 53  Female: 27  Age (yrs)  NR | Type  Liver: 30  Colorectal: 24  Gastric: 11  Pancreatic: 9  BC: 2  Esophageal: 1  Lung: 1  UP: 2  Stage  lV: all | No treatment | Type  Abdominal using straight and point rubbing and kneading  Region  Abdomen | Intensity  Gentle (abdomen  was depressed to a depth of less than 0.5 cm)  Delivery  Researcher | 15 min, 2 x / day for 3 days | Social attention | Type  M:  Temporary skin erythema: 1  Discontinuation  All participants completed the massage intervention. | Severity or seriousness  NR  Last FU  Immediately PI |
| Zorba, 2018 [79]  Hospital (outpatient)  Turkey  N = 75 | Evaluate feasibility and preliminary effects of massage and inhalation aromatherapies on chemo-induced acute nausea/vomiting | Patients receiving highly emetogenic chemo for non-metastatic BC  Exclude: open wounds or edema in lower extremities  Sex  Male: 0  Female: 75  Age (yrs)  All: 45 ±10 ^a^ | Type  BC: all  Stage  ll: 67  llla: 8 | Chemo: all | Type  Swedish with effleurage and petrissage  Region  Feet | Intensity  NR  Delivery  Primary investigator | 20 min, 1x / 21 days for 9 wks (before 2^nd^, 3^rd^ & 4^th^ chemo) | UC  Inhalation essential oils | Type  No AE  Discontinuation  3 in each group discontinued after first intervention- no reason given. | Severity or seriousness  NA  Last FU  Same day PI |
| Retrospective cohort | | | | | | | | | | |
| Author, year  Setting  N | Study aim | Criteria specific to cancer & treatment/  Sex/  Age | Cancer Type/  Stage | Treatment parallel to massage | Type /  Region | Intensity/  Delivery | Time, frequency & duration | Comparator | Outcome | FU period |
| Hsiao, 2015 [83]  Cancer registry from medical center.  Taiwan  N = 1,106 | Assess risk of recurrence in women who developed BCRL and received MLD versus no MLD | Stage 0–3 breast cancer, received surgery with ALND and achieved remission (without clinical evidence of tumor recurrence by day 180 post surgery)  Sex  Female: 1,106  Age (yrs)  MLD: 51.9 ±11.7^a^  C: 52.1 ±11.1^a^ | Type  BC: all  Stage  0–l:34(M), 386(C)  ll–lll:76(M), 610(C) | Chemo:  98 (M)  815 (C)  RT:  60 (M)  428 (C) | Type  MLD  (n = 110)  Region  Initiated at center of neck and trunk. Arm affected limb. | Intensity  Gentle  Delivery  Specialized physiotherapist | 30 min, 2x /wk  Total number varied depending on condition of patient. | No MLD  (n = 996) | Outcome  MLD:  Recurrence: 12 (10.9)  Death: 4 (3.6%)  No MLD:  Recurrence: 154 (15.5)  Death: 55 (5.5)  HR 0.71 (95% CI 0.39-1.29) for recurrence in MLD vs C | Duration of monitoring  3.3 ±1.6 yrs |
| Karda, 2023 [28]  Tertiary hospital,  Indonesia  N = 84 | Evaluate effect of massage manipulation therapy prior to diagnosis and osteosarcoma progressivity | Diagnosed osteosarcoma Jan 2017-Jan 2021 (retrospective)) through Oct 2022 (prospective). Received neoadjuvant chemo; complete clinical, laboratory, and  histopathological data; surgery own institution.  Sex  Male:35 (M), 12 (C)  Female:23 (M), 14 (C)  Age (yrs)  M: 15.5 [5-47]^b^  C: 16 [5-65]^b^ | Type  Stage  NR | No (received massage prior to diagnosis) | Type  Systemic mechanical compression  Region  Over subsequent tumor site  (n = 58) | Intensity  “Certain intensity”  Delivery  Traditional healers | At least one massage prior to diagnosis.  Prior to diagnosis (time frame) not specified  Number of massage treatments:  <3x’s, n =17  ≥3x’s, n =13 | No massage or massage not over tumor area (n = 26) | Outcome  RR 3.06, (95% CI 1.11-6.85) for higher LDH & AP at diagnosis  in M vs C  Time to met (mo):  M (4 [2-11]^b^ vs  C (12 [8-32]^b^  (p<0.001)  No statistical difference between patients with <3 and ≥3 x’s massage, nor in the proportion of patients with metastases at baseline & post-treatment. | Monitored regularly until 1-year FU |
| Prospective cohort | | | | | | | | | | |
| Wang, 2014 [29]  Hospital,  Taiwan  N = 200  (Merged with Wu, 2010 [84]) | Evaluate survival outcomes in patients who had participated in massage prior to diagnosis and those who had not. | Diagnosed with osteosarcoma 1995-2012  Excluded: prior surgery and chemo at other facility.  Sex  Male:72(M), 66(M)  Female:32(M), 30(C)  Age (yrs)  M: 18.3 ±1.1^a^  C: 21.7 ±1.6^a^ | Type  Osteosarcoma (axial and low-grade excluded)  Stage  NR | No (received massage prior to diagnosis) | Type  Massage /manipulative therapy  (n=104)  Region  Included tumor area | Intensity  NR  Delivery  Bonesetter: 36  Chinese med clinic: 32,  Physiotherapist: 2  (Based on Wu, 2010, n=170) | Mean 2.6x’s over mean period of 2.8 wks  Prior to diagnosis (time frame) not specified  (Based on Wu, 2010, n =170) | No massage or massage not over tumor area  (n = 96) | Outcome  5-yr survival rate:  52.9% (M) vs 97.9% (C) (p<0.001)  Recurrence rate:  28.8% (M) vs 6.3% (C) (p<0.001)  Lung mets rate:  38.5% (M) vs 8.3% (C) (p<0.001) | Mean follow-up of 50.2 months (range 7 to 160)  (Based on Wu, 2010, n =170) |

**Legend**: N is based on reported baseline characteristics. All outcome measures reported as n, unless otherwise stated. (Swedish) categorized as such by review authors based on techniques described vs. text specifying this type of massage. *Includes withdrawal, discontinuation, or loss to FU ^a^ Mean±standard deviation. ^b^ Median [range]. Abbreviations: AC, adriamycin-cyclophosphamide; ALL, acute lymphoblastic leukemia; ALND, Axillary lymphnode dissection; AML, acute myelogenous lymphoma; AP, alkaline phosphatease; AR, aromatherapy; AT, attention control; BC, breast cancer; CIPN, chemotherapy-induced peripheral neuropathy; C, control group; CAF, cyclophosphamide-adriamycin-5 fluorouracil; Chemo, chemotherapy; GI, gastrointestinal; GU, genitourinary; Gyn, gynecologic; HL, Hodgkin’s lymphoma; H&N, head and neck; LDH, lactat dehydrogenase; M, massage group; NHL, non-Hodgkin’s lymphoma; NP, Nasopharyngeal: NR, not reported; ROM, range of motion; RT, radiotherapy; SC trans, stemcell transplant; TP, trigger point; UP, Unknown primary; wks, weeks; yrs, years
