# Supplementary material for: Harms of massage in persons with cancer or receiving treatment for cancer: A systematic review and meta-analysis
Source: PLoS One. 2026 Aug 5;21(8):e0351878. doi: 10.1371/journal.pone.0351878 (PMC13440881; doi:10.1371/journal.pone.0351878)
Supplement: Supplementary file 8 [file pone.0351878.s009.docx]

# ROBINS-I tool (Stage II): Çelebioğlu, 2015

## Specify a target randomized trial specific to the study

| Design | Individually randomized |
| --- | --- |
| Participants | Patients with evidence of cancer (with or without treatment) or receiving neoadjuvant or adjuvant treatment for cancer (radiotherapy, chemotherapy, immunotherapy) or were within six weeks of cancer-related surgery. |
| Experimental intervention | Massage plus standard care |
| Comparator | Standard care |

## Is your aim for this study…?

| x | to assess the effect of *assignment to* intervention |
| --- | --- |
|  | to assess the effect of *starting and adhering to* intervention |

## Specify the outcome

Specify which outcome is being assessed for risk of bias (typically from among those earmarked for the Summary of Findings table). Specify whether this is a proposed benefit or harm of intervention.

Discontinuation due to adverse event.

## Specify the numerical result being assessed

In case of multiple alternative analyses being presented, specify the numeric result (e.g. RR = 1.52 (95% CI 0.83 to 2.77) and/or a reference (e.g. to a table, figure or paragraph) that uniquely defines the result being assessed.

Number of discontinuations, text page 800

## Preliminary consideration of confounders

## Complete a row for each important confounding domain (i) listed in the review protocol; and (ii) relevant to the setting of this particular study, or which the study authors identified as potentially important.

#### “Important” confounding domains are those for which, in the context of this study, adjustment is expected to lead to a clinically important change in the estimated effect of the intervention. “Validity” refers to whether the confounding variable or variables fully measure the domain, while “reliability” refers to the precision of the measurement (more measurement error means less reliability).

**(i) Confounding domains listed in the review protocol or relevant to the setting of particular study or which study authors identified as important**

| Confounding domain | Measured variable(s) | Is there evidence that controlling for this variable was unnecessary?* | Is the confounding domain measured validly and reliably by this variable (or these variables)? |
| --- | --- | --- | --- |
| Sociodemographic | Income stratum | Not controlled for. Similar between groups. | Probably- self-reported. |
| Geographical | Place of residence | Not controlled for. Similar between groups. | Probably- self-reported. |
|  |  |  |  |
|  |  |  |  |
|  |  |  |  |
|  |  |  |  |
|  |  |  |  |
|  |  |  |  |

* In the context of a particular study, variables can be demonstrated not to be confounders and so not included in the analysis: (a) if they are not predictive of the outcome; (b) if they are not predictive of intervention; or (c) because adjustment makes no or minimal difference to the estimated effect of the primary parameter. Note that “no statistically significant association” is not the same as “not predictive”.

| **Domain** | **Assessments by outcome** | **Comment** |
| --- | --- | --- |
| **Bias due to confounding** | **Discontinuation due to adverse events.**  *Serious risk* | Lacks information regarding receipt of IT or BMA in groups. |
| **Bias in the selection of participants into the study** | **Discontinuation due to adverse events.**  *Low risk* | The selection of participants into the study or into the analysis was not based on participant characteristics observed after the start of intervention. |
| **Bias in classification of interventions** | **Discontinuation due to adverse events.**  *Low risk* | The intervention group was clearly defined, and participants were assigned by the researcher. |
| **Bias due to deviations form intended intervention** | **Discontinuation due to adverse events.**  *Low risk* | No deviations reported. Only one massage. |
| **Bias due to missing data** | **Discontinuation due to adverse events.**  *Low risk* | Outcome data were available for all, or nearly all, participants. |
| **Bias in measurement of outcomes** | **Discontinuation due to adverse events.**  *Low risk* | We identified no information regarding the measurement of this outcome. However, the assessment of the outcome is unlikely to have been influenced by knowledge of intervention received |
| **Bias in selection of the reported results** | **Discontinuation due to adverse events.**  *Moderate risk* | The reported outcome data are unlikely to have been selected- however nor preregistration of protocol found. |
| **Overall** | **Discontinuation due to adverse events.**  *Serious risk* |  |

# ROBINS-I tool (Stage II): DeOliveira, 2018

## Specify a target randomized trial specific to the study

| Design | Individually randomized |
| --- | --- |
| Participants | Patients with evidence of cancer (with or without treatment) or receiving neoadjuvant or adjuvant treatment for cancer (radiotherapy, chemotherapy, immunotherapy) or were within six weeks of cancer-related surgery. |
| Experimental intervention | Massage plus standard care |
| Comparator | Standard care |

## Is your aim for this study…?

| x | to assess the effect of *assignment to* intervention |
| --- | --- |
|  | to assess the effect of *starting and adhering to* intervention |

## Specify the outcome

Specify which outcome is being assessed for risk of bias (typically from among those earmarked for the Summary of Findings table). Specify whether this is a proposed benefit or harm of intervention.

Adverse events and discontinuation due to adverse event.

## Specify the numerical result being assessed

In case of multiple alternative analyses being presented, specify the numeric result (e.g. RR = 1.52 (95% CI 0.83 to 2.77) and/or a reference (e.g. to a table, figure or paragraph) that uniquely defines the result being assessed.

Events per study arm, Table 2 (wound complications), Table 5 (lymphedema), Figure 1 (loss to follow-up death)

## Preliminary consideration of confounders

## Complete a row for each important confounding domain (i) listed in the review protocol; and (ii) relevant to the setting of this particular study, or which the study authors identified as potentially important.

#### “Important” confounding domains are those for which, in the context of this study, adjustment is expected to lead to a clinically important change in the estimated effect of the intervention. “Validity” refers to whether the confounding variable or variables fully measure the domain, while “reliability” refers to the precision of the measurement (more measurement error means less reliability).

**(i) Confounding domains listed in the review protocol or relevant to the setting of particular study or which study authors identified as important**

| Confounding domain | Measured variable(s) | Is there evidence that controlling for this variable was unnecessary?* | Is the confounding domain measured validly and reliably by this variable (or these variables)? |
| --- | --- | --- | --- |
| Sociodemographic | None reported | Unknown | Yes / No / No information |
| Geographical | None reported | Unknown |  |
|  |  |  |  |
|  |  |  |  |
|  |  |  |  |
|  |  |  |  |
|  |  |  |  |
|  |  |  |  |

* In the context of a particular study, variables can be demonstrated not to be confounders and so not included in the analysis: (a) if they are not predictive of the outcome; (b) if they are not predictive of intervention; or (c) because adjustment makes no or minimal difference to the estimated effect of the primary parameter. Note that “no statistically significant association” is not the same as “not predictive”.

| **Domain** | **Assessments by outcome** | **Comment** |
| --- | --- | --- |
| **Bias due to confounding** | **All outcomes**  *Serious risk* | No information regarding socioeconomic or geographical information is provided. No details regarding how participants were allocated beyond being matched for stage, age and BMI. |
| **Bias in the selection of participants into the study** | **All outcomes**  *Low risk* | Criteria defined before intervention. |
| **Bias in classification of interventions** | **All outcomes**  *Low risk* | The intervention group was clearly defined. |
| **Bias due to deviations form intended intervention** | **All outcomes**  *Low risk* | No deviations described. |
| **Bias due to missing data** | **All outcomes**  *Moderate risk* | 5 in each group lost to follow up during intervention. At 30 mo FU this is higher. Three papers were merged with varying n adding uncertainty regarding missing data. |
| **Bias in measurement of outcomes** | **All outcomes**  *Serious risk* | For objective outcomes lack of blinding not so detrimental, however subjective assessment of wound and lymphedema would be serious |
| **Bias in selection of the reported results** | **All outcomes**  *Low risk* | The reported outcome data are unlikely to have been selected. |
| **Overall** | **All outcomes**  *Serious risk* |  |

# ROBINS-I tool (Stage II): Park, 2015

## Specify a target randomized trial specific to the study

| Design | Individually randomized |
| --- | --- |
| Participants | Patients with evidence of cancer (with or without treatment) or receiving neoadjuvant or adjuvant treatment for cancer (radiotherapy, chemotherapy, immunotherapy) or were within six weeks of cancer-related surgery. |
| Experimental intervention | Massage plus standard care |
| Comparator | Standard care |

## Is your aim for this study…?

|  | X to assess the effect of *assignment to* intervention |
| --- | --- |
|  | to assess the effect of *starting and adhering to* intervention |

## Specify the outcome

Specify which outcome is being assessed for risk of bias (typically from among those earmarked for the Summary of Findings table). Specify whether this is a proposed benefit or harm of intervention.

Discontinuation due to adverse event

## Specify the numerical result being assessed

In case of multiple alternative analyses being presented, specify the numeric result (e.g. RR = 1.52 (95% CI 0.83 to 2.77) and/or a reference (e.g. to a table, figure or paragraph) that uniquely defines the result being assessed.

Events per arm (text page 241)

## Preliminary consideration of confounders

## Complete a row for each important confounding domain (i) listed in the review protocol; and (ii) relevant to the setting of this particular study, or which the study authors identified as potentially important.

#### “Important” confounding domains are those for which, in the context of this study, adjustment is expected to lead to a clinically important change in the estimated effect of the intervention. “Validity” refers to whether the confounding variable or variables fully measure the domain, while “reliability” refers to the precision of the measurement (more measurement error means less reliability).

**(i) Confounding domains listed in the review protocol or relevant to the setting of particular study or which study authors identified as important**

| Confounding domain | Measured variable(s) | Is there evidence that controlling for this variable was unnecessary?* | Is the confounding domain measured validly and reliably by this variable (or these variables)? |
| --- | --- | --- | --- |
| Sociodemographic | None reported | Unknown | Yes / No / No information |
| Geographical | None reported | Unknown |  |
|  |  |  |  |
|  |  |  |  |
|  |  |  |  |
|  |  |  |  |
|  |  |  |  |
|  |  |  |  |

* In the context of a particular study, variables can be demonstrated not to be confounders and so not included in the analysis: (a) if they are not predictive of the outcome; (b) if they are not predictive of intervention; or (c) because adjustment makes no or minimal difference to the estimated effect of the primary parameter. Note that “no statistically significant association” is not the same as “not predictive”.

| **Domain** | **Assessments by outcome** | **Comment** |
| --- | --- | --- |
| **Bias due to confounding** | **Discontinuation due to adverse event**  *Serious risk* | No sociodemographic or geographical data reported. Order of registration could potentially be influenced by variables of importance for receivement of intervention and reason for discontinuation. |
| **Bias in the selection of participants into the study** | **Discontinuation due to adverse event**  *Low risk* | The selection of participants into the study or into the analysis was not based on participant characteristics observed after the start of intervention. |
| **Bias in classification of interventions** | **Discontinuation due to adverse event**  *Low risk* | The intervention group was clearly defined. |
| **Bias due to deviations form intended intervention** | **Discontinuation due to adverse event**  *Low risk* | Low attrition rate. High compliance rate. |
| **Bias due to missing data** | **Discontinuation due to adverse event**  *Low risk* | Missing data for four participants in both group (3 same reason). Still provides sample of 20 in each group- which is the target sample size. |
| **Bias in measurement of outcomes** | **Discontinuation due to adverse event**  *Serious risk* | Self-reported data and participants knew their intervention allocation. |
| **Bias in selection of the reported results** | **Discontinuation due to adverse event**  *Moderate risk* | The reported outcome data are unlikely to have been selected- however no preregistered trial protocol. |
| **Overall** | **Discontinuation due to adverse event**  *Serious risk* |  |

# ROBINS-I tool (Stage II): Wang, 2015

## Specify a target randomized trial specific to the study

| Design | Individually randomized |
| --- | --- |
| Participants | Patients with evidence of cancer (with or without treatment) or receiving neoadjuvant or adjuvant treatment for cancer (radiotherapy, chemotherapy, immunotherapy) or were within six weeks of cancer-related surgery. |
| Experimental intervention | Massage plus standard care |
| Comparator | Standard care |

## Is your aim for this study…?

|  | X to assess the effect of *assignment to* intervention |
| --- | --- |
|  | to assess the effect of *starting and adhering to* intervention |

## Specify the outcome

Specify which outcome is being assessed for risk of bias (typically from among those earmarked for the Summary of Findings table). Specify whether this is a proposed benefit or harm of intervention.

Adverse events and discontinuation due to adverse events.

## Specify the numerical result being assessed

In case of multiple alternative analyses being presented, specify the numeric result (e.g. RR = 1.52 (95% CI 0.83 to 2.77) and/or a reference (e.g. to a table, figure or paragraph) that uniquely defines the result being assessed.

Events per study arm (page 50)

## Preliminary consideration of confounders

## Complete a row for each important confounding domain (i) listed in the review protocol; and (ii) relevant to the setting of this particular study, or which the study authors identified as potentially important.

#### “Important” confounding domains are those for which, in the context of this study, adjustment is expected to lead to a clinically important change in the estimated effect of the intervention. “Validity” refers to whether the confounding variable or variables fully measure the domain, while “reliability” refers to the precision of the measurement (more measurement error means less reliability).

**(i) Confounding domains listed in the review protocol or relevant to the setting of particular study or which study authors identified as important**

| Confounding domain | Measured variable(s) | Is there evidence that controlling for this variable was unnecessary?* | Is the confounding domain measured validly and reliably by this variable (or these variables)? |
| --- | --- | --- | --- |
| Sociodemographic | Education level | Likely not predictive of intervention | Yes / No / No information |
| Geographical | None reported | Unknown |  |
|  |  |  |  |
|  |  |  |  |
|  |  |  |  |
|  |  |  |  |
|  |  |  |  |
|  |  |  |  |

* In the context of a particular study, variables can be demonstrated not to be confounders and so not included in the analysis: (a) if they are not predictive of the outcome; (b) if they are not predictive of intervention; or (c) because adjustment makes no or minimal difference to the estimated effect of the primary parameter. Note that “no statistically significant association” is not the same as “not predictive”.

| **Domain** | **Assessments by outcome** | **Comment** |
| --- | --- | --- |
| **Bias due to confounding** | **All outcomes**  *Moderate risk* | No geographical data reported and only one sociodemographic. Random allocation was used for all participants except for those in rooms with multiple beds. GEE to control for tumor site. Unknown regarding geographical data. |
| **Bias in the selection of participants into the study** | **All outcomes**  *Low risk* | The selection of participants into the study or into the analysis was not based on participant characteristics observed after the start of intervention. |
| **Bias in classification of interventions** | **All outcomes**  *Low risk* | The intervention group was clearly defined, and participants were assigned by the researcher. |
| **Bias due to deviations form intended intervention** | **All outcomes**  *Low risk* | No deviations reported for intended intervention. |
| **Bias due to missing data** | **All outcomes**  *Low risk* | Outcome data were available for all, or nearly all, participants. |
| **Bias in measurement of outcomes** | **All outcomes**  *Moderate risk* | The interventionist assessed the skin each day for erythema, pain, bruises, purpura, and other massage-related adverse events (such as infection, deep vein thrombosis, or ileus) |
| **Bias in selection of the reported results** | **All outcomes**  *Moderate risk* | The reported outcome data are unlikely to have been selected- no preregistered protocol found. |
| **Overall** | **All outcomes**  *Moderate risk* |  |

# ROBINS-I tool (Stage II): Zorba, 2018

## Specify a target randomized trial specific to the study

| Design | Individually randomized |
| --- | --- |
| Participants | Patients with evidence of cancer (with or without treatment) or receiving neoadjuvant or adjuvant treatment for cancer (radiotherapy, chemotherapy, immunotherapy) or were within six weeks of cancer-related surgery. |
| Experimental intervention | Massage plus standard care |
| Comparator | Standard care |

## Is your aim for this study…?

| x | to assess the effect of *assignment to* intervention |
| --- | --- |
|  | to assess the effect of *starting and adhering to* intervention |

## Specify the outcome

Specify which outcome is being assessed for risk of bias (typically from among those earmarked for the Summary of Findings table). Specify whether this is a proposed benefit or harm of intervention.

Adverse events

## Specify the numerical result being assessed

In case of multiple alternative analyses being presented, specify the numeric result (e.g. RR = 1.52 (95% CI 0.83 to 2.77) and/or a reference (e.g. to a table, figure or paragraph) that uniquely defines the result being assessed.

Events per study arm (page 363)

## Preliminary consideration of confounders

## Complete a row for each important confounding domain (i) listed in the review protocol; and (ii) relevant to the setting of this particular study, or which the study authors identified as potentially important.

#### “Important” confounding domains are those for which, in the context of this study, adjustment is expected to lead to a clinically important change in the estimated effect of the intervention. “Validity” refers to whether the confounding variable or variables fully measure the domain, while “reliability” refers to the precision of the measurement (more measurement error means less reliability).

**(i) Confounding domains listed in the review protocol or relevant to the setting of particular study or which study authors identified as important**

| Confounding domain | Measured variable(s) | Is there evidence that controlling for this variable was unnecessary?* | Is the confounding domain measured validly and reliably by this variable (or these variables)? |
| --- | --- | --- | --- |
| Sociodemographic | Education level, occupation | Likely not predictive of intervention | Yes / No / No information |
| Geographical | None reported | Unknown |  |
|  |  |  |  |
|  |  |  |  |
|  |  |  |  |
|  |  |  |  |
|  |  |  |  |
|  |  |  |  |

* In the context of a particular study, variables can be demonstrated not to be confounders and so not included in the analysis: (a) if they are not predictive of the outcome; (b) if they are not predictive of intervention; or (c) because adjustment makes no or minimal difference to the estimated effect of the primary parameter. Note that “no statistically significant association” is not the same as “not predictive”.

| **Domain** | **Assessments by outcome** | **Comment** |
| --- | --- | --- |
| **Bias due to confounding** | **All outcomes**  *Serious risk* | No reporting of geographical data and only one sociodemographic. (No adjustment) |
| **Bias in the selection of participants into the study** | **All outcomes**  *Low risk* | Participants who would have eligible for target trial would be included here. |
| **Bias in classification of interventions** | **All outcomes**  *Low risk* | The intervention group was clearly defined, and participants were assigned by the researcher. |
| **Bias due to deviations form intended intervention** | **All outcomes**  *Low risk* | Low attrition. Compliance as per study design stated. |
| **Bias due to missing data** | **All outcomes**  *Low risk* | Outcome data were available for all, or nearly all, participants. |
| **Bias in measurement of outcomes** | **All outcomes**  *Serious risk* | PI completed observation form for AE- had knowledge of allocation. |
| **Bias in selection of the reported results** | **All outcomes**  *Moderate risk* | The reported outcome data are unlikely to have been selected- however there was no  Pre- registered protocol |
| **Overall** | **All outcomes**  *Serious risk* |  |

# ROBINS-I tool (Stage II): Hsiao, 2015

## Specify a target randomized trial specific to the study

| Design | Individually randomized |
| --- | --- |
| Participants | Patients with evidence of cancer (with or without treatment) or receiving neoadjuvant or adjuvant treatment for cancer (radiotherapy, chemotherapy, immunotherapy) or were within six weeks of cancer-related surgery. |
| Experimental intervention | Massage plus standard care |
| Comparator | Standard care |

## Is your aim for this study…?

| x | to assess the effect of *assignment to* intervention |
| --- | --- |
|  | to assess the effect of *starting and adhering to* intervention |

## Specify the outcome

Specify which outcome is being assessed for risk of bias (typically from among those earmarked for the Summary of Findings table). Specify whether this is a proposed benefit or harm of intervention.

Specific adverse events related to cancer proliferation or metastases

## Specify the numerical result being assessed

In case of multiple alternative analyses being presented, specify the numeric result (e.g. RR = 1.52 (95% CI 0.83 to 2.77) and/or a reference (e.g. to a table, figure or paragraph) that uniquely defines the result being assessed.

Events per study arm (Tables 2 & 3, Figure 1)

## Preliminary consideration of confounders

## Complete a row for each important confounding domain (i) listed in the review protocol; and (ii) relevant to the setting of this particular study, or which the study authors identified as potentially important.

#### “Important” confounding domains are those for which, in the context of this study, adjustment is expected to lead to a clinically important change in the estimated effect of the intervention. “Validity” refers to whether the confounding variable or variables fully measure the domain, while “reliability” refers to the precision of the measurement (more measurement error means less reliability).

**(i) Confounding domains listed in the review protocol or relevant to the setting of particular study or which study authors identified as important**

| Confounding domain | Measured variable(s) | Is there evidence that controlling for this variable was unnecessary?* | Is the confounding domain measured validly and reliably by this variable (or these variables)? |
| --- | --- | --- | --- |
|  |  |  | Yes / No / No information |
|  |  |  |  |
|  |  |  |  |
|  |  |  |  |
|  |  |  |  |
|  |  |  |  |
|  |  |  |  |
|  |  |  |  |

* In the context of a particular study, variables can be demonstrated not to be confounders and so not included in the analysis: (a) if they are not predictive of the outcome; (b) if they are not predictive of intervention; or (c) because adjustment makes no or minimal difference to the estimated effect of the primary parameter. Note that “no statistically significant association” is not the same as “not predictive”.

| **Domain** | **Assessments by outcome** | **Comment** |
| --- | --- | --- |
| **Bias due to confounding** | **Adverse events related to cancer proliferation or metastases**  *Serious risk* | No geographic data and limited sociodemographic. |
| **Bias in the selection of participants into the study** | **Adverse events related to cancer proliferation or metastases**  *Low risk* | Participants who would have eligible for target trial would be included here. |
| **Bias in classification of interventions** | **Adverse events related to cancer proliferation or metastases**  *Low risk* | The intervention group was clearly defined. |
| **Bias due to deviations form intended intervention** | **Adverse events related to cancer proliferation or metastases**  *NI* | No information regarding actual amount of treatments received. |
| **Bias due to missing data** | **Adverse events related to cancer proliferation or metastases**  *Low risk* | Outcome data were available for all, or nearly all, participants. |
| **Bias in measurement of outcomes** | **Adverse events related to cancer proliferation or metastases**  *Low risk* | Data from medical records- objectively measure bloods and evaluations. |
| **Bias in selection of the reported results** | **Adverse events related to cancer proliferation or metastases**  Moderate risk | No preregistered protocol. |
| **Overall** | **Adverse events related to cancer proliferation or metastases**  *Serious risk* |  |

# ROBINS-I tool (Stage II): Karda, 2023

## Specify a target randomized trial specific to the study

| Design | Individually randomized |
| --- | --- |
| Participants | Patients with evidence of cancer (with or without treatment) or receiving neoadjuvant or adjuvant treatment for cancer (radiotherapy, chemotherapy, immunotherapy) or were within six weeks of cancer-related surgery. |
| Experimental intervention | Massage plus standard care |
| Comparator | Standard care |

## Is your aim for this study…?

| x | to assess the effect of *assignment to* intervention |
| --- | --- |
|  | to assess the effect of *starting and adhering to* intervention |

## Specify the outcome

Specify which outcome is being assessed for risk of bias (typically from among those earmarked for the Summary of Findings table). Specify whether this is a proposed benefit or harm of intervention.

Specific adverse events related to cancer proliferation or metastases

## Specify the numerical result being assessed

In case of multiple alternative analyses being presented, specify the numeric result (e.g. RR = 1.52 (95% CI 0.83 to 2.77) and/or a reference (e.g. to a table, figure or paragraph) that uniquely defines the result being assessed.

Events per study arm (Tables 1, 2 & 3)

## Preliminary consideration of confounders

## Complete a row for each important confounding domain (i) listed in the review protocol; and (ii) relevant to the setting of this particular study, or which the study authors identified as potentially important.

#### “Important” confounding domains are those for which, in the context of this study, adjustment is expected to lead to a clinically important change in the estimated effect of the intervention. “Validity” refers to whether the confounding variable or variables fully measure the domain, while “reliability” refers to the precision of the measurement (more measurement error means less reliability).

**(i) Confounding domains listed in the review protocol or relevant to the setting of particular study or which study authors identified as important**

| Confounding domain | Measured variable(s) | Is there evidence that controlling for this variable was unnecessary?* | Is the confounding domain measured validly and reliably by this variable (or these variables)? |
| --- | --- | --- | --- |
|  |  |  | Yes / No / No information |
|  |  |  |  |
|  |  |  |  |
|  |  |  |  |
|  |  |  |  |
|  |  |  |  |
|  |  |  |  |
|  |  |  |  |

* In the context of a particular study, variables can be demonstrated not to be confounders and so not included in the analysis: (a) if they are not predictive of the outcome; (b) if they are not predictive of intervention; or (c) because adjustment makes no or minimal difference to the estimated effect of the primary parameter. Note that “no statistically significant association” is not the same as “not predictive”.

| **Domain** | **Assessments by outcome** | **Comment** |
| --- | --- | --- |
| **Bias due to confounding** | **Adverse events related to cancer proliferation or metastases**  *Critical risk* | Only counts available (no adjustment). Pain prior to diagnosis may be related to prognosis. |
| **Bias in the selection of participants into the study** | **Adverse events related to cancer proliferation or metastases**  *Low risk* | The selection of participants into the study was based on diagnosis of osteosarcoma within a given period- all included based on criteria. |
| **Bias in classification of interventions** | **Adverse events related to cancer proliferation or metastases**  *Serious risk* | The intervention group was clearly defined- however massage data were collected retrospectively in medical records during the majority of the study (2017-2021) and then retrospectively by interview for the remainder of the study period. |
| **Bias due to deviations form intended intervention** | **Adverse events related to cancer proliferation or metastases**  *NI* | Massage participation was collected retrospectively…. And details regarding this are not presented. |
| **Bias due to missing data** | **Adverse events related to cancer proliferation or metastases**  *Low risk* | Outcome data were available for all, or nearly all, participants. |
| **Bias in measurement of outcomes** | **Adverse events related to cancer proliferation or metastases**  *Low risk* | Data from medical records- objectively measure bloods and evaluations. |
| **Bias in selection of the reported results** | **Adverse events related to cancer proliferation or metastases**  *Moderate risk* | The reported outcome data are unlikely to have been selected- however no pre-registered protocol. |
| **Overall** | **Adverse events related to cancer proliferation or metastases**  *Critical risk* |  |

# ROBINS-I tool (Stage II): Wang, 2014 (and Wu, 2010)

## Specify a target randomized trial specific to the study

| Design | Individually randomized |
| --- | --- |
| Participants | Patients with evidence of cancer (with or without treatment) or receiving neoadjuvant or adjuvant treatment for cancer (radiotherapy, chemotherapy, immunotherapy) or were within six weeks of cancer-related surgery. |
| Experimental intervention | Massage plus standard care |
| Comparator | Standard care |

## Is your aim for this study…?

| x | to assess the effect of *assignment to* intervention |
| --- | --- |
|  | to assess the effect of *starting and adhering to* intervention |

## Specify the outcome

Specify which outcome is being assessed for risk of bias (typically from among those earmarked for the Summary of Findings table). Specify whether this is a proposed benefit or harm of intervention.

Specific adverse events related to cancer proliferation or metastases

## Specify the numerical result being assessed

In case of multiple alternative analyses being presented, specify the numeric result (e.g. RR = 1.52 (95% CI 0.83 to 2.77) and/or a reference (e.g. to a table, figure or paragraph) that uniquely defines the result being assessed.

Events per study (Table 1).

## Preliminary consideration of confounders

## Complete a row for each important confounding domain (i) listed in the review protocol; and (ii) relevant to the setting of this particular study, or which the study authors identified as potentially important.

#### “Important” confounding domains are those for which, in the context of this study, adjustment is expected to lead to a clinically important change in the estimated effect of the intervention. “Validity” refers to whether the confounding variable or variables fully measure the domain, while “reliability” refers to the precision of the measurement (more measurement error means less reliability).

**(i) Confounding domains listed in the review protocol or relevant to the setting of particular study or which study authors identified as important**

| Confounding domain | Measured variable(s) | Is there evidence that controlling for this variable was unnecessary?* | Is the confounding domain measured validly and reliably by this variable (or these variables)? |
| --- | --- | --- | --- |
|  |  |  | Yes / No / No information |
|  |  |  |  |
|  |  |  |  |
|  |  |  |  |
|  |  |  |  |
|  |  |  |  |
|  |  |  |  |
|  |  |  |  |

* In the context of a particular study, variables can be demonstrated not to be confounders and so not included in the analysis: (a) if they are not predictive of the outcome; (b) if they are not predictive of intervention; or (c) because adjustment makes no or minimal difference to the estimated effect of the primary parameter. Note that “no statistically significant association” is not the same as “not predictive”.

| **Domain** | **Assessments by outcome** | **Comment** |
| --- | --- | --- |
| **Bias due to confounding** | **Adverse events related to cancer proliferation or metastases**  *Critical risk* | No demographic or geographic data. Very limited patient, treatment or disease related data. Only counts available (no adjustment). Pain prior to diagnosis may be related to prognosis. |
| **Bias in the selection of participants into the study** | **Adverse events related to cancer proliferation or metastases**  *Low risk* | The selection of participants into the study was based on diagnosis of sarcoma within a given period- all included based on criteria. |
| **Bias in classification of interventions** | **Adverse events related to cancer proliferation or metastases**  *Moderate risk* | Details regarding massage collected as self-report retrospectively. |
| **Bias due to deviations form intended intervention** | **Adverse events related to cancer proliferation or metastases**  *NI* | Retrospective. |
| **Bias due to missing data** | **Adverse events related to cancer proliferation or metastases**  *Low risk* | Outcome data were available for all, or nearly all, participants. |
| **Bias in measurement of outcomes** | **Adverse events related to cancer proliferation or metastases**  *Low risk* | Data from medical records- objectively measure bloods and evaluations. |
| **Bias in selection of the reported results** | **Adverse events related to cancer proliferation or metastases**  *Moderate risk* | The reported outcome data are unlikely to have been selected- however no preregistered protocol was identified. |
| **Overall** | **Adverse events related to cancer proliferation or metastases**  *Critical risk* | Disagreement among NK & KB. Await MR decision. |
