# Supplementary material for: Harms of massage in persons with cancer or receiving treatment for cancer: A systematic review and meta-analysis
Source: PLoS One. 2026 Aug 5;21(8):e0351878. doi: 10.1371/journal.pone.0351878 (PMC13440881; doi:10.1371/journal.pone.0351878)
Supplement: Supplementary file 9 [file pone.0351878.s017.docx]

We are very grateful to the editor and reviewers for taking the time to review this manuscript and for constructive comments and suggestions which we believe has improved this work.

| **Reviewer comment** | **Response** |
| --- | --- |
| *Editor* |  |
| **Statistical software specification **(Section 13d, Synthesis methods) While the random-effects model (DerSimonian and Laird method) and the measure of statistical heterogeneity (I² statistic) are appropriately described, the name of the statistical software package used for meta-analysis (e.g., R with metafor package, RevMan, Stata) is not specified. Please add this information to enhance methodological transparency and reproducibility. | Thanks for pointing this out. We have added this information on line 173. |
| **Numerical reporting of primary meta-analysis results **(Section 20b) The overall result of the meta-analysis (Risk Ratio and 95% Confidence Interval) is currently referenced only to Figure 2. For clarity and accessibility, please explicitly report the numerical values of the pooled RR, 95% CI, and I² statistic for the primary analysis within the main text of the Results section. | RR, 95% CI’s and I^2^ statistic have been added in the main text of the results section (line 290). |
| **Statistical reporting of subgroup analyses **(Section 20c) Subgroup analyses are presented qualitatively. If formal statistical tests for subgroup differences were conducted, please report the corresponding p-values for interaction or the I² statistic between subgroups to allow readers to assess whether observed differences across subgroups are statistically meaningful. | Formal statistics for subgroups were performed. The results can be found in Figure 2 and Table 5 with summary statistics including RR and 95% CI’s, I^2^ statistics and P-values for each subgroup. We have not added these statistics to the main text to avoid redundancy. To increase readability, we have added that no “statistical” evidence was observed (line 289) and moved (Figure 2 and Table 5) to the end of sentence (line 292), to better highlight that this figure and table also encompasses the sub-group analyses. |
| ******Operational definition of "deep massage" **(Methods/Eligibility criteria) The manuscript excludes studies assessing "deep massage," yet an operational definition of this term is not provided. Please clarify how "deep massage" was defined for the purposes of study eligibility: Was it based on applied pressure (e.g., in Newtons), depth of tissue penetration, therapist description, or another criterion? This clarification is essential for interpreting the scope and generalizability of the findings. | As per eligibility criteria described on page 5, we did not exclude studies based on “deep massage”. Three studies were included that reported applying deep massage, but none of these studies reported on adverse events and were thus not eligible for further analysis as described on lines 247-249.  Further, we have added extended the discussion of intensity to include how depth/ intensity was reported and a call for future work (lines 353-356) |
| Sensitivity analysis based on adverse event reporting quality: Given the notably low median score for adverse event reporting quality (1.5 out of 16 items), we recommend conducting an additional sensitivity analysis: repeat the meta-analysis including only studies with an adverse event reporting score ≥5. This would help assess the robustness of the pooled estimates to the quality of harms reporting in the primary studies. | This sensitivity analysis has been conducted- results remained stable (S12.) This has been added to the methods section lines 170-171 and results section lines 292-294. |
| **Summary table of key outcomes **(Results) To improve readability and clinical utility, we suggest adding a concise summary table in the Results section. For each primary outcome (e.g., overall adverse events, serious adverse events, local adverse events), the table should include: number of contributing studies, total participants, pooled effect estimate (RR, 95% CI), measure of heterogeneity (I²), and GRADE certainty of evidence. | As per suggestion, we have added a summary table of key outcomes (Table 5). We, however, consider this a redundancy of data from figure 2 which in our view provides a better overview of the analyses. We will leave it up to the reviewers and editor as to the inclusion of both (or one) of these data presentations in the manuscript. |
| **Discussion of generalizability to specific populations **(Discussion) Please add a brief paragraph in the Discussion addressing the generalizability of findings to specific patient subgroups, such as pediatric populations, older adults, or patients with extensive bone metastases. Given the restrictive inclusion criteria of many primary studies (e.g., exclusion of patients with thrombocytopenia, open wounds, or severe symptoms), this discussion would help clinicians appropriately contextualize the results. | We have added a statement regarding generalizability to pediatric and frail populations (lines 347-348).  Considerations regarding bone mets and other cancerous tissues is discussed in line 369 – 379. |
| Formatting and language corrections: Table 4 clarity: In the "Last follow-up" column of Table 4, time points such as "Immediately PI" are ambiguous. Please specify the unit of time (e.g., "Immediately post-intervention (within 24 hours)" or "Immediately post-intervention (same day)") to improve interpretability. | We have amended Table four as per suggestion. |
| *Reviewer 1* |  |
| 3. Have the authors made all data underlying the findings in their manuscript fully available?  Reviewer #1: No | Raw data for meta-analyses **and now** descriptives can be found as supplementary files S14 and S15. This has been noted in the PRISMA checklist. |
| Methods Inclusion criteria - Interventions and comparators Line 102: Please specify the types of massage that were eligible for inclusion, as ‘other types of massage’ is mentioned here without further explanation. In one study manual lymph drainage was included as a type of massage; however, lymph drainage seems to differ from conventional massage in several ways. Please provide more information on the types of massage eligible for inclusion. | We agree that this section was unclear. We have reworded this section and hope that it now reads more clearly (lines 101-110).  Regarding manual lymph drainage- while this is a gentle massage form, it includes techniques that exceed light lotioning (criteria) and is therefore included. |
| Methods  Please add the outcomes of interest to this section. Please provide more information on the types of adverse outcomes addressed. | This was specified in the previous “Data Items” draft, but we have tried to increase clarity by formatting (italics), and by adding (shown here in bold): “outcomes **of interest** (**any** adverse events, lost to follow-up, discontinuation or withdrawal due to adverse event, outcomes related to cancer proliferation or metastases). lines 119-126 |
| Critical appraisal – the use of the PEDro scale should be justified more clearly as some of the items do not represent a bias in clinical trials. Please comment on why the Cochrane RoB Tool 1 or 2 was not used? | We chose the PEDro scale as it is specifically designed for physical therapy interventions, which includes manual therapies like massage (and have added a statement (line 143) reflecting this. As such, we considered it more relevant for massage, rather than the broader Cochrane ROB tool. We acknowledge that RoB 2.0 is a comprehensive tool for assessing risk of bias. However, findings from a meta-epidemiological study* comparing these tools support that either instrument can be used to quantify risk of bias, but that they can’t be used interchangeably.  **Moseley AM, et al. Agreement between the Cochrane risk of bias tool and Physiotherapy Evidence Database (PEDro) scale: a meta-epidemiological study of randomized controlled trials of physical therapy interventions. PLoS One 2019;14(9):e0222770* |
| Analysis In your analysis, all types of cancer were included in one analysis. Is there evidence that there are no differences in the risk of adverse events according to the type of cancer? | Of the 16 studies eligible for primary meta-analysis, 10 studies included mixed cancer populations, 1 breast cancer and 1 colorectal cancer specific- rendering inability to draw cancer type conclusions. Three studies were leukemia specific. These studies ARE analyzed separately as “hematology” in the sub-analysis of cancer stage- with no data to support a difference in AE risk between oncology and hematology populations. We have added a file for reviewer purpose (not publication) to provide an overview of cancer populations in meta-analysis studies. (see file “Cancer type for reviewer”) |
| Results Please add the countries of origin of the included studies to the characteristics of studies. | We agree that geographical information is relevant, which is why it is included in the individual descriptions of study characteristics (S5). We have chosen not to add a geographical summary to Table 1. Instead, we have added a summary text highlighting the regions where the predominance of studies were performed (lines 239-241). In addition, we have also added geographical considerations in the discussion section regarding generalizability (lines 336-341). |
| Table S5 should be included in the main manuscript, not the supplemental material. A description of the included studies seems essential for readers. | We agree that a description of the included studies is important. However, considering the length of the individual study characteristics table and that summary tables of study characteristics, massage characteristics, and adverse events are provided in the manuscript we believe that readability is improved by providing this information for readers as a supplementary file. |
| The results of the critical appraisal of study quality should also be included in the main manuscript (S7 and a summary of S8). | In accordance with comments from the editor we have added a summary table of key outcomes which includes results of study quality /ROB (Table 5). In addition, figure 2 includes a sub-analysis of ROB (where rating of individual studies can be seen).  Previously, S7 presented ROB of all included studies (in two excel arks) For clarity we have now created two separate ROB files; S7 (Risk of bias PEDro) and S8 (Risk of bias ROBINS-I). We hope that these amendments now more clearly report ROB results. |
| Please add the results of the meta-analysis and the GRADE rating to the description in the text (lines 278 ff.). | We have added results of the meta-analysis and GRADE rating to the text (line 290). |
| *Reviewer 2* |  |
| Massage Technique Specifics: The authors have analyzed data based on massage intensity (light, moderate, deep) and type (e.g., Swedish, slow-stroke back). However, they did not explore the potential safety implications of specific manual maneuvers (e.g., swinging, friction, compression, vibration, tapping, or joint mobilization techniques commonly used in Chinese Tuina). Different maneuvers produce significantly different biomechanical effects – for example, tapping techniques may increase bleeding risk in thrombocytopenic patients, while deep kneading could be dangerous for those with bone metastases. Nevertheless, the included primary studies did not report such detailed classifications of techniques, so the authors cannot synthesize this. I suggest adding a paragraph in the Discussion (Limitations) acknowledging the lack of detail on specific techniques in the current literature and calling for future research to clearly report the types of maneuvers used and their association with adverse events. This would strengthen the manual therapy depth of the study. | Based on reviewer comments we reassessed the data to see if we better could present specific massage techniques used in the included studies. This has resulted in a revision of Table 2 which now includes a row specifying abdominal massage. In addition we have added ”techniques” after Swedish with a footnote specifying which techniques were considered swedish. Also, in Table S5 (charactersitics of individual studies) we have added the specific massage techniques reported vs fx. only writing ”swedish”. While this still does not allow for synthesis of specific massage techniques we believe that it now better describes massage techniques used. We have also added a statement (lines 354-356) highlighting lack of detail and calling for clear reporting in future studies. |
| Distinction Between Absolute and Relative Contraindications: In the Introduction, the authors note that clinical massage textbooks cite cancer as a contraindication. It would be helpful to explicitly distinguish between absolute and relative contraindications, as the main findings suggest that most light-to-moderate intensity massage is safe in screened patients**.** Furthermore, the authors do not address differences between massage systems (e.g., Chinese Tuina vs. Western massage). In China, most Tuina textbooks classify tumors as an absolute contraindication, which has severely limited clinical research on Tuina for cancer patients in China. Consequently, almost all included studies originate from Western countries, and the generalizability of the conclusions to Tuina practice in China requires caution. I recommend that the authors briefly mention this geographical and cultural difference in the Discussion to help readers understand the limitations of the evidence base. | Beyond the introduction we have refrained from referring to absolute and relative contraindications but have instead referred to general contraindications (meaning contraindications to the general population). However, as this can be confused with “general contraindications” referring to specific systemic conditions where massage should be avoided (e.g. fever, infectious disease) we have deleted this term throughout the manuscript and have just used the term contraindication (encompassing both absolute and relative contraindications.)  In general, we have avoided use of these categories, but have highlighted conditions where massage likely should be avoided or modified:  Discussion  (lines 342-347)  Further, in line with concerns that potential adverse effects of cancer treatment are contraindications to massage, characteristics of the population of included studies should be noted. Specifically, related to exclusion criteria, more than half of the studies reporting on adverse events excluded participants if they presented with adverse events related to cancer or cancer treatment such as low platelet count, fever, or wounds/ lesions in the target massage area (S5). This is important to consider in the generalizability of massage to the larger population of cancer survivors.  (lines 357-362)  Light intensity massage is used in “oncology massage” as it is practiced and promoted by many schools [12,25,26]. Nonetheless, cancer survivors may seek massage where deep intensities are warranted for better effect (e.g. scar tissue or chronic soft tissue injury) [123–125]. In these cases, it is prudent to ensure that no contraindications to massage (e.g. thrombocytopenia or wounds/lesions) exist before engaging in deep tissue massage (as it seems reasonable to assume that any adverse effect induced by massage would be exacerbated by deeper intensity).  (lines 374-379)  As such, though we cannot present evidence to support or refute concerns that massage over superficial cancerous tissue may increase the risk of local damage, in cases with, for example, open wounds, radiation dermatitis or bone metastases, avoidance of massage or modifications in intensity are sensible precautions as per contraindications for massage.  Conclusion  (lines 427- 435):  Massage practitioners should familiarize themselves with the contraindications for massage, many of which may be more frequent in oncology patients. This study is unable to assess the safety of deep massage in oncology. As such, special care should be taken (e.g. screening for contraindications), especially before engaging in deeper tissue massage.  Due to uncertainty as to whether massage over tumor in osteosarcoma constitutes an increased risk of negative survival outcomes, massage directly on tumor is discouraged, though definitive evidence is lacking.  Regarding generalizability related to geographical and cultural differences: this is now adressed in the discussion (lines 336- 341). |
| Specific Risks for Weakened Skin: On page 10, lines 67-68, after mentioning "weakened skin from surgery and radiotherapy," please consider adding specific associated risks (e.g., infection, breakdown of radiation dermatitis). | We have rephrased this sentence to address the comment and added a reference (31) related to radiation-induced skin injury (lines 67-68). |
| Adverse Event Definition and Grading: The study uses the NCI CTCAE definition of adverse events. However, it is not explicitly stated whether AEs were collected according to CTCAE grades (1-5). The authors should clarify this. The results note that only one study reported on seriousness (grade 3-5), and none reported on severity grading – this should be stated more prominently. | We have added a statement regarding adverse event grading and use of CTCAE (lines 127-129).  Thank you for your attention to detail regarding seriousness and severity. In the process of addressing this comment we found a mistake in our categorization of seriousness /severity in Kim, 2024. As this study reported on adverse events and graded their *severity,* we have ammended the text (lines 263-265), table 3 and supplementary file 6 to reflect this.  This section (lines 263-265) has also been reworded to highlight the absence of these outcomes in adverse event reporting. |
| Highlighting Key Finding on Deep Massage: In Table 2 ("Massage intensity"), three studies involving "deep" massage did not report adverse events. This is a critical piece of information that should be highlighted more prominently in the main text (it is only briefly mentioned on lines 237-239). Consider emphasizing this finding in the Results or Discussion. | The three studies involving deep massage did not report **on** adverse events (scored zero points in the CONSORT quality of adverse event reporting checklist) and were therefore not eligible for further analysis. Beyond Table 2, this is also stated in the results section (lines 247-248). As such, any assessment of adverse events from these studies is inconclusive, which is highlighted in the discussion (lines 350-353). These findings are further discussed in the following paragraph in the context of conditions warranting deep massage and securing that no contraindications exist (lines 357-368). |
| Suggested New Table Column: Consider adding a column to Table 2 (or a new supplemental table) indicating whether massage was applied directly over known tumor sites. This would help distinguish the safety risks associated with local massage on cancerous tissue versus massage on distant areas. | Table 2 has been amended to reflect massage over cancerous tissue in both intervention studies and observational studies. These findings are discussed in the discusssion section (lines 369-402) |
