# Supplementary material for: Harms of massage in persons with cancer or receiving treatment for cancer: A systematic review and meta-analysis
Source: PLoS One. 2026 Aug 5;21(8):e0351878. doi: 10.1371/journal.pone.0351878 (PMC13440881; doi:10.1371/journal.pone.0351878)
Supplement: Peer review history

# Peer review: Decision Letter 0

**DOI:** 10.1371/journal.pone.0351878.r001

**Authors:** Aynollah Naderi

6 Apr 2026

PONE-D-25-52742

Harms of massage in persons with cancer or receiving treatment for cancer: a systematic review and meta-analysis

PLOS One

Dear Dr. Kjerulf,

Thank you for submitting your manuscript to PLOS ONE. After careful consideration, we feel that it has merit but does not fully meet PLOS ONE’s publication criteria as it currently stands. Therefore, we invite you to submit a revised version of the manuscript that addresses the points raised during the review process.

Please submit your revised manuscript by May 21 2026 11:59PM. If you will need more time than this to complete your revisions, please reply to this message or contact the journal office at plosone@plos.org. When you're ready to submit your revision, log on to https://www.editorialmanager.com/pone/ and select the 'Submissions Needing Revision' folder to locate your manuscript file.

Please include the following items when submitting your revised manuscript:

- A letter that responds to each point raised by the academic editor and reviewer(s). You should upload this letter as a separate file labeled 'Response to Reviewers'.
- A marked-up copy of your manuscript that highlights changes made to the original version. You should upload this as a separate file labeled 'Revised Manuscript with Track Changes'.
- An unmarked version of your revised paper without tracked changes. You should upload this as a separate file labeled 'Manuscript'.

If you would like to make changes to your financial disclosure, please include your updated statement in your cover letter. Guidelines for resubmitting your figure files are available below the reviewer comments at the end of this letter.

If applicable, we recommend that you deposit your laboratory protocols in protocols.io to enhance the reproducibility of your results. Protocols.io assigns your protocol its own identifier (DOI) so that it can be cited independently in the future. For instructions see: https://journals.plos.org/plosone/s/submission-guidelines#loc-laboratory-protocols. Additionally, PLOS ONE offers an option for publishing peer-reviewed Lab Protocol articles, which describe protocols hosted on protocols.io. Read more information on sharing protocols at https://plos.org/protocols?utm_medium=editorial-email&utm_source=authorletters&utm_campaign=protocols.

As the corresponding author, your ORCID iD is verified in the submission system and will appear in the published article. PLOS supports the use of ORCID, and we encourage all coauthors to register for an ORCID iD and use it as well. Please encourage your coauthors to verify their ORCID iD within the submission system before final acceptance, as unverified ORCID iDs will not appear in the published article. *Only*  the individual author can complete the verification step; PLOS staff *cannot*  verify ORCID iDs on behalf of authors.

We look forward to receiving your revised manuscript.

Kind regards,

Aynollah Naderi

Academic Editor

PLOS One

Journal Requirements:

When submitting your revision, we need you to address these additional requirements.

1. Please ensure that your manuscript meets PLOS ONE's style requirements, including those for file naming. The PLOS ONE style templates can be found at

https://journals.plos.org/plosone/s/file?id=wjVg/PLOSOne_formatting_sample_main_body.pdf and https://journals.plos.org/plosone/s/file?id=ba62/PLOSOne_formatting_sample_title_authors_affiliations.pdf

If the reviewer comments include a recommendation to cite specific previously published works, please review and evaluate these publications to determine whether they are relevant and should be cited. There is no requirement to cite these works unless the editor has indicated otherwise.

Please review your reference list to ensure that it is complete and correct. If you have cited papers that have been retracted, please include the rationale for doing so in the manuscript text, or remove these references and replace them with relevant current references. Any changes to the reference list should be mentioned in the rebuttal letter that accompanies your revised manuscript. If you need to cite a retracted article, indicate the article’s retracted status in the References list and also include a citation and full reference for the retraction notice.

Additional Editor Comments:

Specific Comments to the Authors

**Statistical software specification **(Section 13d, Synthesis methods)

While the random-effects model (DerSimonian and Laird method) and the measure of statistical heterogeneity (I² statistic) are appropriately described, the name of the statistical software package used for meta-analysis (e.g., R with metafor package, RevMan, Stata) is not specified. Please add this information to enhance methodological transparency and reproducibility.

**Numerical reporting of primary meta-analysis results **(Section 20b)

The overall result of the meta-analysis (Risk Ratio and 95% Confidence Interval) is currently referenced only to Figure 2. For clarity and accessibility, please explicitly report the numerical values of the pooled RR, 95% CI, and I² statistic for the primary analysis within the main text of the Results section.

**Statistical reporting of subgroup analyses **(Section 20c)

Subgroup analyses are presented qualitatively. If formal statistical tests for subgroup differences were conducted, please report the corresponding p-values for interaction or the I² statistic between subgroups to allow readers to assess whether observed differences across subgroups are statistically meaningful.

**Operational definition of "deep massage" **(Methods/Eligibility criteria)

The manuscript excludes studies assessing "deep massage," yet an operational definition of this term is not provided. Please clarify how "deep massage" was defined for the purposes of study eligibility: Was it based on applied pressure (e.g., in Newtons), depth of tissue penetration, therapist description, or another criterion? This clarification is essential for interpreting the scope and generalizability of the findings.

Sensitivity analysis based on adverse event reporting quality:

Given the notably low median score for adverse event reporting quality (1.5 out of 16 items), we recommend conducting an additional sensitivity analysis: repeat the meta-analysis including only studies with an adverse event reporting score ≥5. This would help assess the robustness of the pooled estimates to the quality of harms reporting in the primary studies.

**Summary table of key outcomes **(Results)

To improve readability and clinical utility, we suggest adding a concise summary table in the Results section. For each primary outcome (e.g., overall adverse events, serious adverse events, local adverse events), the table should include: number of contributing studies, total participants, pooled effect estimate (RR, 95% CI), measure of heterogeneity (I²), and GRADE certainty of evidence.

**Discussion of generalizability to specific populations **(Discussion)

Please add a brief paragraph in the Discussion addressing the generalizability of findings to specific patient subgroups, such as pediatric populations, older adults, or patients with extensive bone metastases. Given the restrictive inclusion criteria of many primary studies (e.g., exclusion of patients with thrombocytopenia, open wounds, or severe symptoms), this discussion would help clinicians appropriately contextualize the results.

Formatting and language corrections:

Table 4 clarity: In the "Last follow-up" column of Table 4, time points such as "Immediately PI" are ambiguous. Please specify the unit of time (e.g., "Immediately post-intervention (within 24 hours)" or "Immediately post-intervention (same day)") to improve interpretability.

[Note: HTML markup is below. Please do not edit.]

Reviewers' comments:

Reviewer's Responses to Questions

**Comments to the Author**

1. Is the manuscript technically sound, and do the data support the conclusions?

The manuscript must describe a technically sound piece of scientific research with data that supports the conclusions. Experiments must have been conducted rigorously, with appropriate controls, replication, and sample sizes. The conclusions must be drawn appropriately based on the data presented.

Reviewer #1: Yes

Reviewer #2: Yes

**********

2. Has the statistical analysis been performed appropriately and rigorously?

Reviewer #1: Yes

Reviewer #2: Yes

**********

3. Have the authors made all data underlying the findings in their manuscript fully available?

The PLOS Data policy requires authors to make all data underlying the findings described in their manuscript fully available without restriction, with rare exception (please refer to the Data Availability Statement in the manuscript PDF file). The data should be provided as part of the manuscript or its supporting information, or deposited to a public repository. For example, in addition to summary statistics, the data points behind means, medians and variance measures should be available. If there are restrictions on publicly sharing data—e.g. participant privacy or use of data from a third party—those must be specified.

Reviewer #1: No

Reviewer #2: Yes

**********

4. Is the manuscript presented in an intelligible fashion and written in standard English?

PLOS ONE does not copyedit accepted manuscripts, so the language in submitted articles must be clear, correct, and unambiguous. Any typographical or grammatical errors should be corrected at revision, so please note any specific errors here.

Reviewer #1: Yes

Reviewer #2: Yes

**********

5. Review Comments to the Author

Please use the space provided to explain your answers to the questions above. You may also include additional comments for the author, including concerns about dual publication, research ethics, or publication ethics. (Please upload your review as an attachment if it exceeds 20,000 characters)

Reviewer #1: Thank you very much for giving me the opportunity to review this interesting manuscript. The paper is well-written and methodologically sound. You will find my comments below, which may help you to further improve the paper.

Methods

Inclusion criteria - Interventions and comparators

Line 102: Please specify the types of massage that were eligible for inclusion, as ‘other types of massage’ is mentioned here without further explanation. In one study manual lymph drainage was included as a type of massage; however, lymph drainage seems to differ from conventional massage in several ways. Please provide more information on the types of massage eligible for inclusion.

Please add the outcomes of interest to this section. Please provide more information on the types of adverse outcomes addressed.

Critical appraisal – the use of the PEDro scale should be justified more clearly as some of the items do not represent a bias in clinical trials. Please comment on why the Cochrane RoB Tool 1 or 2 was not used?

Analysis

In your analysis, all types of cancer were included in one analysis. Is there evidence that there are no differences in the risk of adverse events according to the type of cancer?

Results

Please add the countries of origin of the included studies to the characteristics of studies.

Table S5 should be included in the main manuscript, not the supplemental material. A description of the included studies seems essential for readers.

The results of the critical appraisal of study quality should also be included in the main manuscript (S7 and a summary of S8).

Please add the results of the meta-analysis and the GRADE rating to the description in the text (lines 278 ff.).

Reviewer #2: This study addresses a clinically important topic: massage is widely used among cancer patients, yet robust evidence on its safety is lacking. A systematic assessment of its potential harms is essential for clinical practice and patient safety. The manuscript presents a clear research question that directly addresses a key clinical concern (the safety of massage in cancer patients). The methodology is robust, including PROSPERO registration, PRISMA reporting, comprehensive multi-database searches, independent dual screening and data extraction, risk of bias assessment using the PEDro and ROBINS-I tools, and GRADE评级 for certainty of evidence. The results are presented in detail, and the systematic evaluation of the quality of adverse event reporting provides an important cautionary signal.

Recommendations for Revision:

Massage Technique Specifics: The authors have analyzed data based on massage intensity (light, moderate, deep) and type (e.g., Swedish, slow-stroke back). However, they did not explore the potential safety implications of specific manual maneuvers (e.g., swinging, friction, compression, vibration, tapping, or joint mobilization techniques commonly used in Chinese Tuina). Different maneuvers produce significantly different biomechanical effects – for example, tapping techniques may increase bleeding risk in thrombocytopenic patients, while deep kneading could be dangerous for those with bone metastases. Nevertheless, the included primary studies did not report such detailed classifications of techniques, so the authors cannot synthesize this. I suggest adding a paragraph in the Discussion (Limitations) acknowledging the lack of detail on specific techniques in the current literature and calling for future research to clearly report the types of maneuvers used and their association with adverse events. This would strengthen the manual therapy depth of the study.

Distinction Between Absolute and Relative Contraindications: In the Introduction, the authors note that clinical massage textbooks cite cancer as a contraindication. It would be helpful to explicitly distinguish between absolute and relative contraindications, as the main findings suggest that most light-to-moderate intensity massage is safe in screened patients. Furthermore, the authors do not address differences between massage systems (e.g., Chinese Tuina vs. Western massage). In China, most Tuina textbooks classify tumors as an absolute contraindication, which has severely limited clinical research on Tuina for cancer patients in China. Consequently, almost all included studies originate from Western countries, and the generalizability of the conclusions to Tuina practice in China requires caution. I recommend that the authors briefly mention this geographical and cultural difference in the Discussion to help readers understand the limitations of the evidence base.

Specific Risks for Weakened Skin: On page 10, lines 67-68, after mentioning "weakened skin from surgery and radiotherapy," please consider adding specific associated risks (e.g., infection, breakdown of radiation dermatitis).

Adverse Event Definition and Grading: The study uses the NCI CTCAE definition of adverse events. However, it is not explicitly stated whether AEs were collected according to CTCAE grades (1-5). The authors should clarify this. The results note that only one study reported on seriousness (grade 3-5), and none reported on severity grading – this should be stated more prominently.

Highlighting Key Finding on Deep Massage: In Table 2 ("Massage intensity"), three studies involving "deep" massage did not report adverse events. This is a critical piece of information that should be highlighted more prominently in the main text (it is only briefly mentioned on lines 237-239). Consider emphasizing this finding in the Results or Discussion.

Suggested New Table Column: Consider adding a column to Table 2 (or a new supplemental table) indicating whether massage was applied directly over known tumor sites. This would help distinguish the safety risks associated with local massage on cancerous tissue versus massage on distant areas.

**********

6. PLOS authors have the option to publish the peer review history of their article (what does this mean?). If published, this will include your full peer review and any attached files.

If you choose “no”, your identity will remain anonymous but your review may still be made public.

**Do you want your identity to be public for this peer review?**  For information about this choice, including consent withdrawal, please see our Privacy Policy.

Reviewer #1: No

Reviewer #2: **Yes:** Zhitong Deng

**********

[NOTE: If reviewer comments were submitted as an attachment file, they will be attached to this email and accessible via the submission site. Please log into your account, locate the manuscript record, and check for the action link "View Attachments". If this link does not appear, there are no attachment files.]

To ensure your figures meet our technical requirements, please review our figure guidelines: https://journals.plos.org/plosone/s/figures

You may also use PLOS’s free figure tool, NAAS, to help you prepare publication quality figures: https://journals.plos.org/plosone/s/figures#loc-tools-for-figure-preparation.

NAAS will assess whether your figures meet our technical requirements by comparing each figure against our figure specifications.

# Author comment: Author response to Decision Letter 1

**DOI:** 10.1371/journal.pone.0351878.r002

22 May 2026

Please see file "response to reviewers".

Attachment Submitted filename: Response to reviewers.docx

# Editor report: Decision Letter 1

**DOI:** 10.1371/journal.pone.0351878.r003

**Authors:** Aynollah Naderi

2 Jun 2026

Harms of massage in persons with cancer or receiving treatment for cancer: a systematic review and meta-analysis

PONE-D-25-52742R1

Dear Dr. Nicolas Kjerulf,

We’re pleased to inform you that your manuscript has been judged scientifically suitable for publication and will be formally accepted for publication once it meets all outstanding technical requirements.

Within one week, you’ll receive an e-mail detailing the required amendments. When these have been addressed, you’ll receive a formal acceptance letter and your manuscript will be scheduled for publication.

An invoice will be generated when your article is formally accepted. Please note, if your institution has a publishing partnership with PLOS and your article meets the relevant criteria, all or part of your publication costs will be covered. Please make sure your user information is up-to-date by logging into Editorial Manager at Editorial Manager® and clicking the ‘Update My Information' link at the top of the page. For questions related to billing, please contact billing support.

If your institution or institutions have a press office, please notify them about your upcoming paper to help maximize its impact. If they’ll be preparing press materials, please inform our press team as soon as possible -- no later than 48 hours after receiving the formal acceptance. Your manuscript will remain under strict press embargo until 2 pm Eastern Time on the date of publication. For more information, please contact onepress@plos.org.

Kind regards,

Aynollah Naderi

Academic Editor

PLOS One

Additional Editor Comments (optional):

Reviewers' comments:

# Editor report: Acceptance letter

**DOI:** 10.1371/journal.pone.0351878.r004

**Authors:** Aynollah Naderi

PONE-D-25-52742R1

PLOS One

Dear Dr. Kjerulf,

I'm pleased to inform you that your manuscript has been deemed suitable for publication in PLOS One. Congratulations! Your manuscript is now being handed over to our production team.

At this stage, our production department will prepare your paper for publication. This includes ensuring the following:

* All references, tables, and figures are properly cited

* All relevant supporting information is included in the manuscript submission,

* There are no issues that prevent the paper from being properly typeset

You will receive further instructions from the production team, including instructions on how to review your proof when it is ready. Please keep in mind that we are working through a large volume of accepted articles, so please give us a few days to review your paper and let you know the next and final steps.

Lastly, if your institution or institutions have a press office, please let them know about your upcoming paper now to help maximize its impact. If they'll be preparing press materials, please inform our press team within the next 48 hours. Your manuscript will remain under strict press embargo until 2 pm Eastern Time on the date of publication. For more information, please contact onepress@plos.org.

You will receive an invoice from PLOS for your publication fee after your manuscript has reached the completed accept phase. If you receive an email requesting payment before acceptance or for any other service, this may be a phishing scheme. Learn how to identify phishing emails and protect your accounts at https://explore.plos.org/phishing.

If we can help with anything else, please email us at customercare@plos.org.

Thank you for submitting your work to PLOS ONE and supporting open access.

Kind regards,

PLOS ONE Editorial Office Staff

on behalf of

Dr. Aynollah Naderi

Academic Editor

PLOS One
